# Diversification of flowering plants in space and time

**DOI:** 10.1038/s41467-023-43396-8

**Published:** 2023-11-22

**Authors:** Dimitar Dimitrov, Xiaoting Xu, Xiangyan Su, Nawal Shrestha, Yunpeng Liu, Jonathan D. Kennedy, Lisha Lyu, David Nogués-Bravo, James Rosindell, Yong Yang, Jon Fjeldså, Jianquan Liu, Bernhard Schmid, Jingyun Fang, Carsten Rahbek, Zhiheng Wang

**Affiliations:** 1 Institute of Ecology and Key Laboratory for Earth Surface Processes of the Ministry of Education, College of Urban and Environmental Sciences, Peking University, Beijing, 100871 China; 2 Department of Natural History, University Museum of Bergen, University of Bergen, P.O. Box 7800, 5020 Bergen, Norway; 3 Center for Macroecology, Evolution and Climate, GLOBE Institute, University of Copenhagen, Universitetsparken 15, 2100 Copenhagen, Denmark; 4 Natural History Museum, University of Oslo, PO Box 1172 Blindern, NO-0318 Oslo, Norway; 5 Key Laboratory of Bio-Resource and Eco-Environment of Ministry of Education, College of Life Sciences, Sichuan University, Chengdu, 610065 Sichuan China; 6 Land Consolidation and Rehabilitation Center, Ministry of Natural Resources, Beijing, 100035 China; 7 State Key Laboratory of Herbage Improvement and Grassland Agro-ecosystems, College of Ecology, Lanzhou University, Lanzhou, 730000 Gansu China; 8 Natural History Museum of Denmark, University of Copenhagen, DK-2100 Copenhagen Ø, Denmark; 9 Department of Animal and Plant Sciences, University of Sheffield, Sheffield, UK; 10 School of Urban Planning and Design, Shenzhen Graduate School, Peking University, Shenzhen, 518055 Shenzhen China; 11 Department of Life Sciences, Imperial College London, Silwood Park Campus, Ascot, Berkshire, SL5 7PY UK; 12 Co-Innovation Center for Sustainable Forestry in Southern China, College of Life Sciences, Nanjing Forestry University, 159 Longpan Rd., Nanjing, 210037 China; 13 Department of Geography, University of Zurich, Winterthurerstrasse 190, 8057 Zurich, Switzerland; 14 Danish Institute for Advanced Study, University of Southern Denmark, Odense, Denmark

**Keywords:** Biodiversity, Evolution, Macroecology, Biodiversity, Adaptive radiation

## Abstract

The rapid diversification and high species richness of flowering plants is regarded as ‘Darwin’s second abominable mystery’. Today the global spatiotemporal pattern of plant diversification remains elusive. Using a newly generated genus-level phylogeny and global distribution data for 14,244 flowering plant genera, we describe the diversification dynamics of angiosperms through space and time. Our analyses show that diversification rates increased throughout the early Cretaceous and then slightly decreased or remained mostly stable until the end of the Cretaceous–Paleogene mass extinction event 66 million years ago. After that, diversification rates increased again towards the present. Younger genera with high diversification rates dominate temperate and dryland regions, whereas old genera with low diversification dominate the tropics. This leads to a negative correlation between spatial patterns of diversification and genus diversity. Our findings suggest that global changes since the Cenozoic shaped the patterns of flowering plant diversity and support an emerging consensus that diversification rates are higher outside the tropics.

## Abstract (web summary)

Global spatiotemporal patterns of plant diversification are unclear. Here, the authors use a genus-level phylogeny and global distribution data for 14,244 flowering plant genera, finding a negative correlation between spatial patterns of diversification and genus diversity.

## Introduction

Flowering plants are a major component of the biosphere providing food and habitats for terrestrial animals^1^. They have adapted to and diversified in a wide variety of environments^2,3^. Understanding the evolutionary processes underlying the global spatiotemporal patterns of flowering plant diversity has intrigued ecologists and biogeographers since the time of von Humboldt^4^ and still represents an unresolved issue in biology. Previous studies have illustrated a pervasive latitudinal diversity gradient for flowering plants^5,6^. Among many factors hypothesized to explain the latitudinal diversity gradient, macroevolutionary processes, including variations in net diversification rates and the time available for speciation, have played a key role^7,8^.

Although previous attempts to understand the diversification of flowering plants have explored the influences of variation in global climate, geography, and ecological opportunities on diversification rates^9–11^, the temporal and spatial trends of species diversification at a global scale for flowering plants are yet to be established. Most previous analyses have either been based on family-level phylogenies^9,12–14^_,_but see^15^ or have relied exclusively on the fragmented fossil records^16,17^. The lack of a comprehensive time-calibrated phylogeny with higher taxonomic resolution and better-resolved distributional data have limited our understanding of the diversity patterns of flowering plants and the macroevolutionary mechanisms underlying them.

**Fig. 1:**
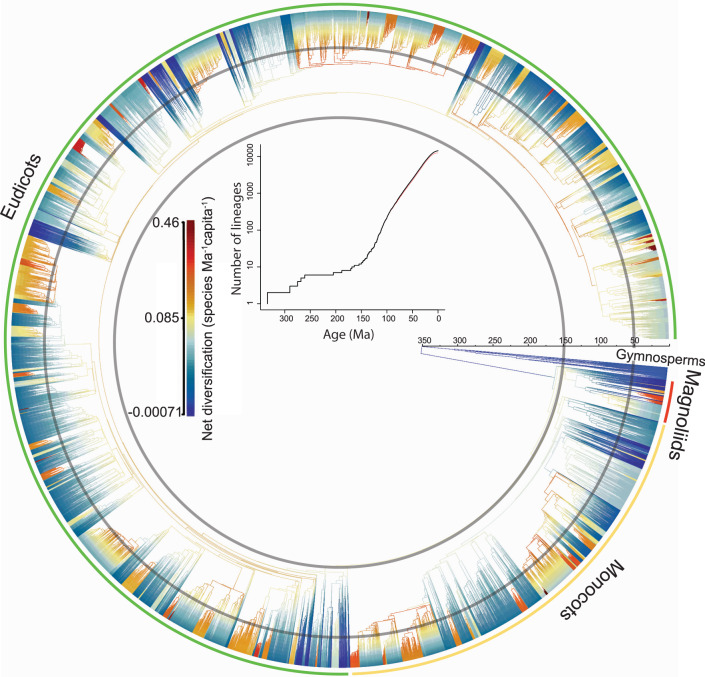
Net diversification rates through time across all flowering plant genera. Warmer and colder colors denote faster and slower rates, respectively. The insert shows the lineage through time plot for all flowering plant genera based on the global (red line) and molecular (black line) phylogenies.

Here, we elucidate the spatiotemporal diversification dynamics of the flowering plants and their relationships with the global patterns of flowering plant diversity by integrating two global datasets: (1) a time-calibrated phylogeny containing 14,244 currently recognized genera (87.5% with DNA sequences based on sequences from 22,277 species) of flowering plants and (2) a dataset of the global distribution of 13,719 genera at a spatial resolution of ca. 329,670 km^2^ (mean area: 329,670 ± 198,191 km^2^) from > 1100 data sources, which are mostly regional species lists and to a lesser extend species occurrence records due to the limited availability of the latter (Supplementary Data 1). The phylogeny of angiosperm genera is constructed using maximum likelihood (ML) with the divergence between orders constrained following the APG IV framework (see Methods), and is dated using 100 fossils^10,13,14^ (Supplementary Data 2) under three dating scenarios for the crown age of flowering plants: (1) 140–150 Ma^16^, (2) 140–210 Ma^18^, and (3) 149–256 Ma^19^ (see Methods). Although the crown age of angiosperms varies across the three dating scenarios, the estimated genus and family ages are, in general, consistent with recent estimations based on fossil and molecular^13^ evidence (Table 1), but see^19^. Our phylogeny provides a global overview of angiosperm genera relationships (Fig. 1), and significantly expands the coverage of angiosperm genera compared to available large scale angiosperm phylogenies^20,21^.

**Table 1:** Median values of divergence time (age), speciation rates, and net diversification rates for flowering plant genera. Minimum and maximum values are shown in brackets

Tree Types	Dating constrains (Ma)	Genus Age (Ma)	Speciation rates	Net diversification rates
Molecular phylogeny	140–150	21.20 (0.005–150)	0.058 (0.022–0.524)	0.056 (−0.003–0.463)
	140–210	22.19 (0.005–210)	0.056 (0.024–0.468)	0.055 (−0.089–0.413)
	149–259	23.16 (0.005–256)	0.054 (0.019–0.424)	0.053 (−0.100–0.383)
Global phylogeny	140–150	19.50 (0.005–150)	0.057 (0.028–0.527)	0.055(0.001–0.4463)
	140–210	19.50 (0.005–210)	0.056 (0.024–0.470)	0.053 (−0.009–0.413)
	149–259	21.38 (0.005–256)	0.054 (0.020–0.424)	0.052 (−0.045–0.383)

Minimum and maximum values are shown in brackets.

## Results and discussion

### Temporal trends of flowering plant diversification

**Fig. 2:**
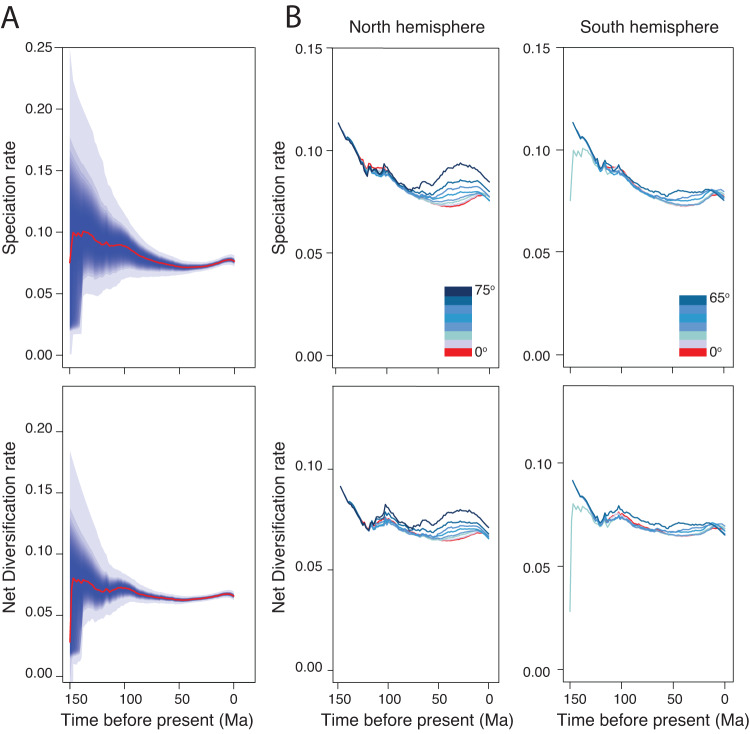
Variation in speciation and net diversification rates through time. Rates of speciation and net diversification through time of flowering plants estimated at a global scale (**A**) and for different latitude belts (**B**). Evolutionary rates are estimated using the global phylogeny with the crown of flowering plants constrained to 140–150 Ma. The shaded areas surrounding the solid lines represent the 95% confidence intervals of the mean rate estimates. In **B**, the evolutionary rates are estimated for latitudinal belts of 10 degrees across the two hemispheres. Darker colors indicate higher latitudes, and the red line indicates the Equator belt (−5 ^o^ to 5 ^o^).

**Fig. 3:**
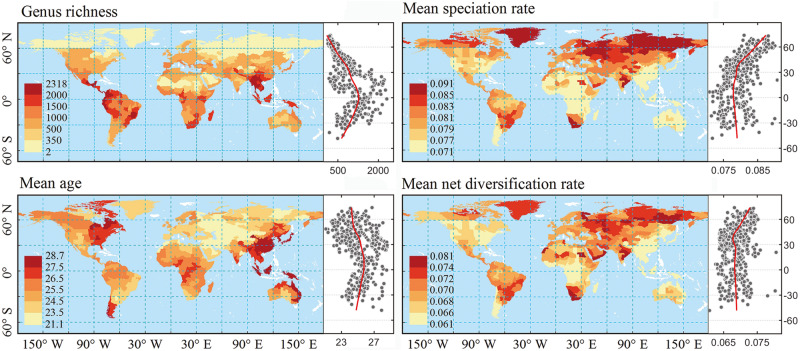
Global patterns and latitudinal gradients of angiosperm generic diversity, mean genus age, mean speciation rates and mean net diversification rates. Generic diversity is a quadratic function of latitude (*r*^*2*^ = 0.42): diversity is the highest in tropical regions and decreases towards the poles. Mean genus age and mean evolutionary rates are estimated using the global phylogeny with angiosperms crown age constrained to 140–150 Ma. Regions with no distributional data are shown in white. Solid red lines on the scatter plots represent lowess regression with span of 0.5. The same results based on only monophyletic genera are shown in Fig. S9.

The analyses of speciation rates and net diversification rates through time demonstrate two diversification bursts in the evolution of flowering plants. The first one occurred between the Late Jurassic (ca. 150 Ma) and the mid-Cretaceous (ca. 100 Ma)^3,16^ and this time period roughly coincides with the time when flowering plants started to increase their abundance in terrestrial floras before rising to dominance towards the end of the Cretaceous^22^. This burst in flowering plant diversification is also corroborated by both fossil^16^ and pollen^23^ records. All our dating scenarios suggest that from the late Cretaceous to the Paleocene-Eocene Thermal Maximum (PETM), speciation rates and net diversification rates slowed down or remained mostly stable (Figs. 2 and S1–S4). The second diversification burst of flowering plants started after the PETM with overall speciation rates and net diversification rates continuously increasing towards the present. However, speciation and net diversification rates significantly vary across lineages (Fig. 1) and are relatively higher in temperate and dryland-adapted genera (Figs. 2, 3, S5, and S6).

Previous studies have interpreted the difference between the stem and crown ages of angiosperm families as evidence for a period of low diversification rates between the initial burst of angiosperm diversity in the first half of Cretaceous and their fast diversification in the Cenozoic^14^. This period of low diversification coincides with the time when the extent of tropical-like habitats was reduced due to global climate cooling^24,25^ followed by a major turnover in plant communities across the globe and a decline in non-flowering plants^16,26^. These environmental changes might have required flowering plants to acquire novel adaptations. Studies have indeed suggested that the low and stable rates of angiosperm diversification between the mid-Cretaceous and the Cenozoic might be the effect of the time necessary for angiosperms to develop morphological and ecological innovations after the early split between major angiosperm lineages^14^. However, the evidence for quick diversification of angiosperms during periods characterized by abrupt environmental changes that we find (see below) suggests that other factors may have been involved. For example, competition with non-flowering seed plants and ferns which remained a major component of terrestrial plant communities until the late Cretaceous when they experienced dramatic increase in extinction rates and decrease in diversification rates^16^.

The speciation of flowering plants rapidly increased during the Cenozoic, especially in temperate and dryland-adapted genera (Figs. 2, S5, and S6). This may be partly due to increased ecological opportunities after the K-Pg mass extinction^27^ and the expansion of temperate and dryland habitats due to generally continuous process of global cooling and aridification from the PETM towards the present^28^, especially in high latitudes^29^. For example, geological evidence shows a drastic decrease in temperature at the high latitudes of Eurasia from the late Eocene to the early Oligocene, leading to the expansion of temperate habitats^30^. As a result, the ancient angiosperm lineages^31^ that were likely adapted to cooler environments experienced quick diversification^10,31^. In addition, the retreat of the Paratethys sea between 20–30 Ma generated a vast new terrestrial area in northern Africa and Southwest and Central Asia^32^ and also intensified aridity since the early Miocene, especially during the last 10 Ma, thereby creating large drylands around the globe^33^. This aridification likely accelerated the speciation rates of many dryland adapted genera^34^ (Figs. 2 and S6). Studies on birds show a similar pattern of extensive speciation and high net diversification rates in the Cenozoic, which has been linked to the expansion of new habitats^28^.

Our genus-level angiosperm phylogenetic tree improves the phylogenetic resolution compared to family-level trees, henceforth, provides finer temporal resolution on the diversification history of angiosperms compared to previous studies conducted at the family-level or on smaller species-level trees and confirms the generality of these overall patterns^9,12–15^. However, the lack of resolution at the specie-level in our study may have led to an underestimation of the most recent diversification trend, particularly for genera where different intrageneric lineages have highly divergent diversification histories (Figs. 1 and 2). This might be the likely reason for the apparent decline of diversification rate in the last 15–20 Ma when most extant genera originate.

### Spatial patterns of flowering plant diversity, age, and diversification

The current genus diversity of flowering plants generally shows a significant latitudinal gradient, decreasing from the tropics towards the poles, with a notable outlier in the Fynbos of South Africa (Fig. 3). The Andes, Central America and Southeast Asia harbor the highest generic diversity (>2100 genera per ca 4^o^ × 4^o^ geographical unit), while temperate regions and the continuous arid zone ranging from northern Africa through southwestern Asia to central Asia and Mongolia (Afro-Asian drylands hereafter, see Methods) have the lowest generic diversity (<1000 genera per geographical unit in Afro-Asian drylands and temperate Eurasia). This global pattern is not biased by the size of geographical units and the number of distribution data sources that we used to compile the distributional data (Fig. S7). However, in some regions such as the Indochina peninsula (Cambodia, Laos, Vietnam), species distribution data are relatively insufficient, which may lead to underestimation of genus richness.

**Fig. 4:**
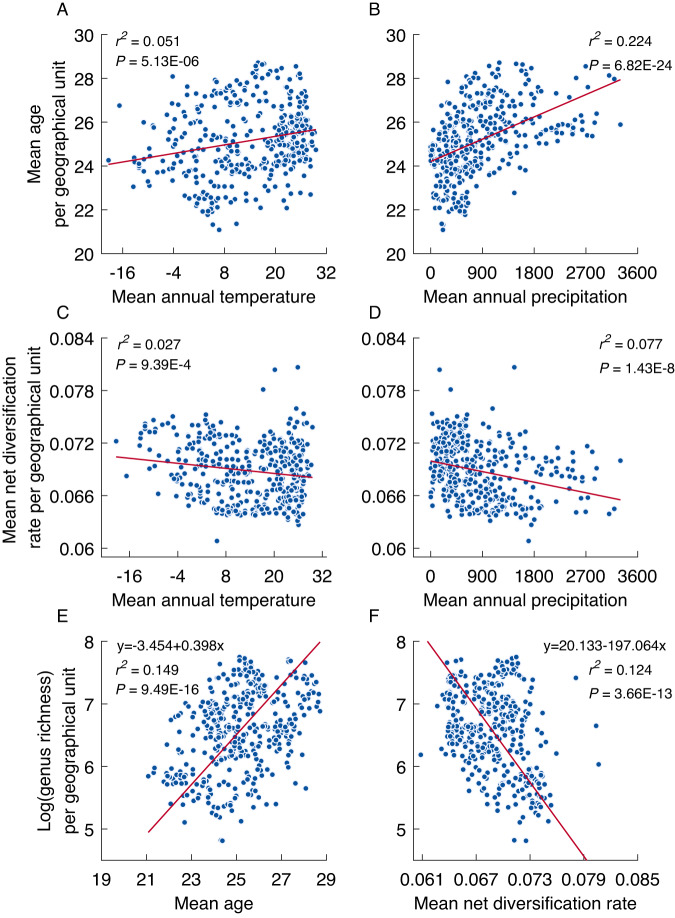
Variation in genus age and net diversification rate as a function of annual temperature and precipitation, and in generic diversity as a finction of genus age and net diversification. Variation in mean genus age (**A**, **B**) and mean net diversification rate (**C**, **D**) per geographic unit as a function of mean annual temperature and mean annual precipitation. Variation in the log-transformed generic diversity as a function of mean genus age (**E**) and mean net diversification rate (**F**) per geographical unit. Mean age and mean net diversification rate are based on the global phylogeny with angiosperms crown age constrained to 140–150 Ma. Solid red lines represent linear (**A**–**D**) and model II geometric mean regression line (**E**, **F**). All *p-*values (*P*) and *r*2 were estimated by linear regression.

We find pronounced latitudinal gradients of mean genus age, mean speciation rate, and mean net diversification rate per geographical unit. Specifically, the mean genus age per geographical unit is the oldest in the tropics and decreases with latitude in both hemispheres (Figs. 3 and S8–S10) and increases with mean annual temperature and precipitation (Fig. 4A, B). In contrast, the mean speciation and net diversification rates per geographical unit increase with latitude (Figs. 3 and S8–S10) and decrease with mean annual temperature and precipitation (Fig. 4C, D), reaching the highest values in temperate Eurasia and the Afro-Asian drylands (Figs. 3, S8, and S9). The latitudinal gradient in genus age, mean speciation and net diversification rates differed significantly from a null model assuming random spatial distributions of genera (Fig. S11), which suggests that the genera outside the tropics are not a random subset of angiosperms, but are significantly biased towards taxa with high speciation and net diversification rates and young ages. More interestingly, we find that the latitudinal gradients in mean speciation and net diversification rates have persisted since the Cenozoic (Figs. 2 and S5) to the present. The relationships of mean genus age and mean net diversification rate with mean annual temperature and precipitation remain unchanged when five quantiles (i.e., 5%, 25%, 50%, 75%, and 95%) instead of average values of mean annual temperature and precipitation within geographic units were used to present the spatial variations in climate (Fig. S12). Furthermore, geographic variations in mean genus age and mean net diversification rate are not significantly correlated with climatic heterogeneity within geographic units (Fig. S13). These results suggest that climatic heterogeneity within geographic units does not bias our findings on the relationships between mean genus age/net diversification rate and climate.

**Fig. 5:**
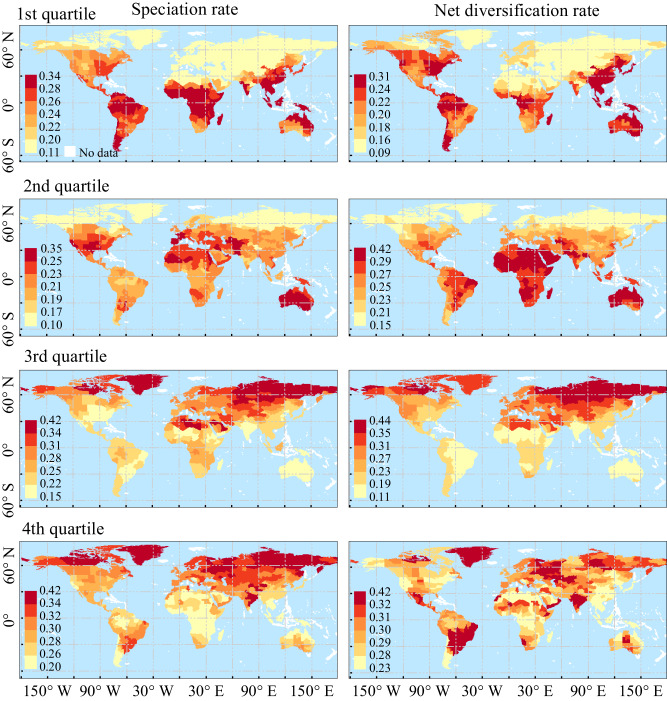
Geographical variation in the proportions of genera with different evolutionary rates. All genera are divided into four quartiles according to their species-level speciation and net diversification rates respectively. From the 1st to the 4th quartiles, the evolutionary rates increase. The proportion of each quartile in each geographical unit is calculated. All analyses are based on the global phylogeny with angiosperms crown age constrained to 140–150 Ma. The same results based on other phylogenies with different dating constraints are shown in Figs. S20–S24.

When we classified all genera into four quartiles according to their ages, speciation, or net diversification rates respectively (see Methods), we find that the oldest genera (stem age >30.83 Ma) and those with the lowest speciation and net diversification rates have the highest dominance in the tropical and subtropical floras. Their proportion in floras decreases with latitude and is the lowest in temperate Eurasia and the Afro-Asian drylands (Figs. 5 and S14–S19). In contrast, the youngest genera (stem age <11.24 Ma) and those with the highest speciation and net diversification rates have much higher contribution to temperate and dryland floras compared to floras in other regions (Figs. 5 and S20–S24).

Our results show markedly higher speciation and net diversification rates and younger ages of genera in drylands and temperate regions, despite low generic diversity in these regions (Figs. 3, S6, S8, and S9). Among all temperate and dryland regions, the floras of temperate Eurasia and the Afro-Asian drylands have genera with the youngest ages and the highest speciation and net diversification rates (Fig. 3). In the 280 flowering plant families inhabiting these regions, including large families such as Fabaceae and Poaceae (Supplementary Data 3), ~54% of these families have higher speciation and net diversification rates within these regions than in the rest of their distribution ranges. A further evaluation demonstrated that the endemic genera in these regions are significantly younger and diversify significantly faster than the endemic genera in tropical/subtropical regions (Wilcoxon rank-sum two-sided test, *p* = 6.048e–08, Fig. S6). In contrast to temperate and dryland regions, the diversity hotspots in tropical and subtropical regions are characterized by clades with relatively low speciation and net diversification rates, and old ages (>25 Ma) (Fig. 3). This suggests that tropical and subtropical regions may have accumulated species over longer periods of time in comparison to other regions of the world and might have served as a museum for flowering plants during long-term climate change, see also^15,35^.

Both geological and evolutionary processes may have contributed to the young age and high diversification rate of the floras in temperate Eurasia and the Afro-Asian drylands. For example, both regions experienced dramatic environmental changes in the Cenozoic, which may have provided new habitats for the radiation of flowering plants. A sharp decrease of temperature in temperate Eurasia during the early Oligocene might have also caused rapid expansion of temperate habitats in this region^30^. Furthermore, the retreat of the Paratethys Sea (20–30 Ma) created new terrestrial and arid areas in northern Africa as well as Southwest and Central Asia^32^ since the early Miocene^33^. These dramatic environmental changes have provided a large variety of new habitats for the rapid radiation of cold- and arid-adapted flowering plants. Moreover, the initiation of the east Asian monsoons in the early Miocene, and their later intensification in the mid-Miocene, may have led to more windy environments in temperate Eurasia^36^. This, in turn, may have enhanced the diversification of wind-pollinated families, such as Poaceae which are common in these regions^37^.

Herbaceous and small shrub species usually have higher proportions in dryland and temperate floras than tree species. Previous studies demonstrate that, compared to tree species, herbaceous species have higher rates of molecular evolution likely due to their shorter generation times^38^. In addition, herbaceous species and small shrubs tend to have higher ploidy levels^39–41^. Both of this may have also contributed to the younger age and higher speciation and net diversification rates in drylands and temperate regions (Fig. S25). In addition, a large component of the floras in temperate Eurasia and the Afro-Asian drylands are Crassulacean acid metabolism (CAM) plants^42,43^. These plants have experienced rapid diversification since the mid-Miocene in temperate and arid regions^42,44,45^. Indeed, we find that genera dominated by CAM species have higher diversification rates than those dominated by C3 plants (Fig. S25). The rapid expansion of CAM plants to newly formed habitats in temperate Eurasia and the Afro-Asian drylands since the mid-Miocene may have further contributed to high speciation and net diversification rates in these regions.

### The role of evolutionary processes on the global patterns of flowering plant diversity

We find a negative correlation between genus diversity of flowering plants and the mean diversification rates per geographical unit (modified *t-*test: correlation coefficient = −0.352, *Fstat* = 0.141, degrees of freedom = 54.229, *p* = 0.009, effective sample size = 52.229), but a positive correlation between genus diversity and mean genus age per geographical unit (modified *t-*test: correlation coefficient = 0.386, *Fstat* = 0.175, degrees of freedom = 32.771, *p* = 0.021, effective sample size = 33.771) (Figs. 4E, F and S26, and Tables S1 and S2). Our null model results (Fig. S11B, C) suggest that the observed relationships between genus richness and mean net diversification rate/mean genus age are not due to random processes. The negative relationship between genus diversity and mean net diversification rate per geographic unit is in contrast with the long-standing diversification-rate hypothesis, which states that a decrease in net diversification rate from the tropics to the poles drives the latitudinal gradient in species diversity of flowering plants^46^. Instead, our results are in line with the predictions of the time-for-speciation hypothesis and suggest that longer time for speciation in the tropics compared with other areas^47^ may have played a critical role in shaping the global patterns of flowering plants diversity. Similar conclusions have also been achieved in recent studies on other groups of organisms, e.g., birds, mammals, and turtles^28,48,49^.

Geological evidence indicates that the tropical climate has likely been present in the equatorial area during the Cretaceous and the early Cenozoic and has persisted ever since^50^, while the temperate in high latitudes (such as the temperate Eurasia) and arid climates in Afro-Asian drylands may have arisen only since the mid-Cenozoic^51,52^. Therefore, these temperate and dryland regions may have had much less time for species accumulation than the tropics. Our analysis also reveals that genera originating before the mid-Cenozoic are mostly restricted to tropical and subtropical climates, while those radiating in the cold and arid regions have mostly originated after the mid-Cenozoic and, in general, have higher diversification rates (Figs. 3, 5, S8, S9, and S14–S24). Fossil records^53^ and spatial patterns in family crown ages of flowering plants^14^ show consistent patterns. Together these findings indicate that the current latitudinal gradient in species diversity may have been formed only in the last 30–40 Ma following the expansion of modern temperate climate and drylands, especially in the Northern Hemisphere^54^. In addition to this effect of time for speciation, other factors such as differences in the area of tropical and temperate regions through time^55^ or ecological constraints^56^ may have also played an important role in the establishment of the current latitudinal gradient in species diversity of flowering plants. Their relative effects compared with evolutionary processes should be tested in future studies.

### Summary

Our results suggest that flowering plants have experienced two bursts of diversification, which agrees with paleontological data^3^. Extant flowering plant species are mainly derived from the second diversification burst where intense global cooling and aridification induced a rapid diversification of species in newly emerged habitats. Across different biomes, the temperate and dryland regions in Eurasia and northern Africa host angiosperm genera with the youngest ages and the highest speciation and net diversification rates. Moreover, the global diversity pattern of angiosperms is negatively correlated with mean speciation and net diversification rates, suggesting that processes other than speciation and net diversification rates may have driven the global diversity patterns of flowering plants. Our study demonstrates the necessity of integrating species distributions with mega-phylogenies to understand the mechanisms underlying large-scale biodiversity patterns.

## Methods

### Phylogenetic reconstruction

#### Sequence downloading and quality screening

We downloaded all sequence data of seed plants available in GenBank (as of 19 May 2018) for the following genes commonly used in plant phylogenetic studies: *18* *S* ribosomal DNA (*18* *S* rDNA), *internal transcribed spacer* region (*ITS*, including *ITS1*, *5.8S* ribosomal DNA and *ITS2*), and *26S* ribosomal DNA (*26S* rDNA) from the nuclear genome; *ATPase β-subunit* gene (*atpB*), *Maturase K* (*matK*), *NADH dehydrogenase F* (*ndhF*) and *ribulose bisphosphate carboxylase large chain* (*rbcL*) from the chloroplast genome; and *Maturase R* (*matR*) from the mitochondrial genome. The *ITS1*, *5.8S,* and *ITS2* were downloaded and treated as a single fragment referred to as *ITS* herein. This gene sample represents both quickly (e.g., *ITS*) and slowly (e.g., *18S* rDNA, *rbcL*, *matR*) evolving genes. Sequence download and quality checks were managed using the NCBIminer v. 4.0^57^. In total, the raw data included 669,619 records of seed plant DNA sequences for 132,373 infrageneric taxa and 457 families.

We first filtered the raw data to species level following a few simple rules. 1) Sequences belonging to hybrids or from taxa that were not identified to the genus level according to the GenBank taxonomic database were removed. 2) Only the longest sequence of each species was kept for each genetic marker. When more than one sequence was found as longest (due to equal lengths), the most recently published sequence was kept. 3) Sequences used in published peer-reviewed papers were preferred to those not used in publications. The dataset resulting from this procedure included 260,477 sequences from 124,646 infrageneric taxa. To avoid errors due to mismatches between GenBank taxonomy and the taxonomy that we applied to the distributional data, we updated the taxonomy of these sequences using the same procedure as the one applied to the distributional data (see Species and genus names section).

To improve the coverage of genetic markers for each genus, we used congeneric sequences for some genera. Because genera may be non-monophyletic, we assessed the monophyly of each genus with sequence data using the large species-level phylogeny of ref. ^20^. The tree of ref. ^20^ is based on few genetic markers and contains a huge proportion of missing data thus it is not free of issues and is likely not able to provide a conclusive test of genera monophyly (e.g., genera may be inferred to be non-monophyletic due to lack of data). However, when taxa described in the same genus are found to form a monophyletic group it provides evidence that these genera are likely monophyletic. A total of 593 genera (4.7%) were identified as non-monophyletic. We screened carefully all non-monophyletic genera. 1) For the non-monophyletic genera caused by very few stochastic intruders from other genera, we removed the intruders’ sequences from our database. 2) For those non-monophyletic genera with several clades, we identified all the monophyletic clades and estimated the number of species included in the tree of ref. ^20^ for each clade. Then the largest clade of each non-monophyletic genus, was used to represent the genus. 3) For polyphyletic genera, we only selected species from the core monophyletic clades. These steps ensure that we only combine sequences from species that form monophyletic groups in the cases where sequences in our final dataset were from multiple species.

To minimize the number of non-conspecific sequences representing monophyletic composite genera while maximizing the coverage of genetic markers for each genus, we developed the following complementary method for sequence filtering. (1) For a genus, we sorted the genetic markers in an ascending order of the number of species per marker. (2) For the first marker (i.e. the marker with the least number of species), we selected the species covering the highest number of markers and (when the number of markers was the same for more than one species) the highest total relative sequence length. In this way, selected species may also cover the remaining genetic markers. (3) We repeated the above procedure for all genetic markers until the maximum number of genetic markers for each genus was achieved. 4) As the above procedure might lead to the selection of multiple species for each marker, we then selected the longest sequences for each marker to maximize the total number of base pairs for each genus in the final matrix. The relative sequence length was calculated as the number of base pairs of a genetic marker for a species divided by the maximum number of base pairs of the marker for the specific genus.

Using the outlined procedure, we produced a final matrix for 12,539 seed plant genera representing ca. 87.5% of the known seed plant genera (87.4% of the angiosperms and 100% of the gymnosperms) based on sequences from 22,277 species. We downloaded sequences for 9 species of ferns as outgroups. The list of accession numbers and taxonomic information for all genera in the final dataset is given in Supplementary Data 4.

The outlined procedure has an important aspect that should be highlighted. The use of one representative sequence per genetic marker per genus as placeholder of the genus could lead to composite terminals for large genera (especially those with more than 3 species) as different genetic markers may come from different species. Composite terminals represented ca. 46% of the genera in the final molecular dataset. About 52% of these composite terminals had sequence data from only 2 species, and 48% had sequence data from 3 or more species (Fig. S27, see Supplementary Data 4 for the species list used for each genus). This approach should not result in biases and artifacts in the resulting phylogenies due to the following reasons. 1) We do not intend to address any relationships within genera (i.e., at the species level). 2) We used only taxa that could be unambiguously assigned to accepted genera (e.g., we did not use sequences that were ambiguously assigned to genera following the GenBank sequence annotations). 3) Recent studies on mega-phylogenies indicate that most accepted genera are recovered as monophyletic even when analyzing large and highly incomplete molecular super-matrices, suggesting that the current taxonomy of flowering plant genera is overall well supported by available molecular data^10,20^. In cases where genera were found to be non-monophyletic we made sure that sequences of composite terminals come only from one monophyletic lineage representing the bulk of species diversity in these taxa. Composite taxa approach has been proven advantageous as it not only helps to reduce the computational demands for data analyses but also significantly increase phylogenetic accuracy by decreasing the amount of missing data (i.e. gaps in the supermatrix of DNA data) in most cases^58^. This approach has been successfully used in recent studies^59,60^. Therefore, composite terminals after data screening in our study should not influence higher-level relationships and might minimize the potential uncertainties on the estimate of the diversification history for flowering plants based on the available DNA data for a limited number of species.

#### Sequence alignment

To make sure that different accessions are oriented in the same direction we performed the following steps for each gene. 1) We selected the two longest sequences for every order and wrote these sequences into a single fasta file. 2) We aligned these representative sequences using MAFFT^61^ and the L-INS-i algorithm with the commands *--localpair -- maxiterate 1000 --adjustdirectionaccurately*. This step generated an alignment of the longest sequences of all orders in the same direction. Let Alignment0 denote this alignment. 3) We separated all available sequences into different files, one file per order, and sorted them in order of decreasing sequence length. Therefore, the longest sequences of each order were always on top. 4) We replaced the longest two sequences in all files with the corresponding sequences from Alignment0 whose directions had been adjusted. 5) Finally, we aligned the sequences of each order respectively using the same algorithm: *--localpair -- maxiterate 1000 --adjustdirectionaccurately*. These steps ensure that the sequences within each order and across orders are in the same direction.

Because the alignment of some gene regions (particularly the ITS1 and ITS2) between very divergent groups is difficult and may lead to unwanted artefacts, we adopted an alignment strategy with the following steps. 1) The sequences of each plant order were placed in a separate matrix and were aligned using the L-INS-i strategy in MAFFT with the following commands: *--localpair -- maxiterate 1000 --adjustdirectionaccurately* (step 5 above). 2) The order-level alignments of each gene were merged as a single fasta file. For each of these combined fasta files *subMSAtable* file with the information on which sequences correspond to individual order-level alignments was created using the *makemergetable.rb* script distributed with MAFFT. 3) The order-level alignments for each gene were then aligned to each other in MAFFT using the *--localpair --merge* commands that allow alignment of multiple sequence alignments. 4) The resulting aligned matrices of all genes were concatenated to each other to generate a super-matrix of aligned sequences, which was then used in subsequent phylogenetic analyses. All alignments were conducted at the High-performance Computing Platform of Peking University.

It is worth mentioning that the separate order-level alignments will, although indirectly, introduce to some extent a soft constraint on orders monophyly even if some orders were not explicitly constraint to be monophyletic (see next section). This approach is consistent with the topological constraints that we used for the deeper nodes on the backbone topology in the following analysis and is better than other alternatives as it decreases alignment errors and ensures higher consistency with currently accepted taxonomy.

#### Phylogenetic analyses

Phylogenetic analyses were run using RAxML v 8.0.26^62^ at the High-performance Computing Platform of Peking University and at the Abel cluster at the University of Oslo. Data was partitioned by gene and the GTRGAMMA model (with parameters optimized independently for each partition) was used for evaluation of the tree likelihood. To ensure better consistency between our analysis and the current knowledge on higher-level seed plant relationships, e.g., above family level, we constrained the phylogenetic analyses using the currently accepted view on relationships among angiosperm orders and among eudicots, monocots and magnoliids^63^. This approach is similar to the one undertaken in recent large-scale comparative studies of angiosperms^10,20^ but differs in the level at which topological constraints were applied. We opted to apply the constraints on the topology at the deeper nodes of the tree (i.e., at order level), as these are more likely to present problems given the character sample for this study (for example, some of these relationships have been elucidated using morphological data that is not used in our analyses). In contrast, in the analysis by ref. ^10^, a family-level topology was used as a backbone constraint for their phylogeny reconstruction.

The topology resulting from the RAxML analysis was then subjected to molecular dating using penalized likelihood as implemented in the program treePL^64^. In our analyses, we used 100 fossil calibration points described in recent refs. ^10,13,14^. Among these ref. ^14^. provides the most comprehensive list of curated fossil calibrations (see the Data2b_CalibrationList.xls supplementary file of ref. ^14^). A complete list of the publications describing these fossils is provided in Supplementary Data 2. In previous studies using large plant taxon samples, the strict molecular clock has been rejected^10^. Therefore, we did not repeat this test here. In the penalized likelihood dating analysis, the selection of the smoothing value parameter may influence age estimates due to its effect on substitution rates estimates. With the increase of smoothing value, the rate heterogeneity decreases and more clock-like mode of rate evolution is assumed^65^. To select appropriate smoothing value for our dating analyses, we performed a cross validation with smoothing parameter set to 0.0001, 0.001, 0.1, and 100 (the default setting in treePL). Due to the large size of the dataset and the computational burden of full cross-validation, we did not test all potential values of the smoothing parameter. Based on the *χ*^2^ test, the result of these cross validation runs suggested that the lowest smoothing value (0.0001) is preferred. Using a smoothing value of 0.0001 in our dating runs under different constraints for the age of the crown angiosperms showed higher congruence in age estimates with the fossil record and previous studies. Moreover, the smoothing value = 0.0001 did not generate bias towards much younger ages in some groups such as Gymnosperms, while the use of higher smoothing values in alternative test runs did. Thus, based on the results from the random cross valuation of the three different dating runs and to account for potentially very large differences in rates among lineages (particularly between angiosperms and gymnosperms), we have chosen the setting with a very small smoothing value (0.0001) for the final penalized likelihood analyses. The preference for such small smoothing values is consistent with previous studies of large clades with heterogeneous rates of molecular evolution where even lower settings for the smoothing parameter were found to be appropriate^66^.

Dating runs based on all combinations of the fossil constraints and the four levels of the smoothing values that we tested found that the ages for the crown Angiosperms substantially exceeded the commonly referred age for this node, i.e. 140–150 Ma^18^. The time of origin of the crown Angiosperms is still debated and different studies have provided estimates that varied from a maximum of about 280 Ma^67^ to a minimum of 130 Ma^68^ with virtually all possible values in-between. As differences in age estimates may have significant impacts on the following diversification analyses, we used an additional constraint on the age of the crown Angiosperms to investigate the potential effects on diversification induced by the uncertainties associated with the dating of this node. This additional constraint was designed based on the overview of angiosperm dating studies and fossil evidence provided in refs. ^18, 19^. Three different settings were used: the first incorporated a wider temporal interval (min = 149 Ma, max = 256 Ma) based on the results of ref. ^19^ to accommodate the most common average ages for this group; the second took a median estimate of the age of this group (min = 140 Ma, max = 210 Ma); and the third was more restrictive encompassing the highest probability density for the average age of the crown Angiosperms at ca. 145 Ma (min = 140, max = 150 Ma) as suggested by ref. ^16^. A similar approach of using various dating strategies to account for the uncertainties in the age estimates for crown angiosperms was recently adopted in a study showing global patterns of angiosperms families^14^.

#### Construction of the global genus-level phylogeny

To include all currently described seed plant genera (see Supplementary Data 4 for the genera list) in our phylogeny, we added genera without sequence data into the dated phylogeny according to their family-level placement or according to the order level placement if family placement was uncertain. Specifically, all genera that were not represented in the molecular dataset were added to the crown nodes of their corresponding families or orders as polytomies. To speed up the analyses the topology of each order containing taxa added following this procedure was extracted and polytomies were resolved following the methods of^69^, with BEAST v1.8.0^70^. The polytomy resolver method uses the input topology and branch length information to generate an xml file that can be then directly analyzed in BEAST estimating both branch lengths and phylogenetic placement of taxa for which character data is not available simultaneously. The polytomy resolver analysis was run in BEAST until stationary was achieved in the MCMC chain. For the BEAST analyses we used a birth-death model with uniform priors for the mean growth rate (λ − μ) and the relative death rate (μ/λ) parameters. We used this model set up as it is intended to be wildly applicable when additional information on the model priors is not available (as in our case)^69^. Each BEAST run was run for 11 million generations and posteriors were sampled every 1000 generations. After discarding the first 10% of generations as a burn-in, we used Tracer v1.6.0^71^ to examine the effective sampling sizes (ESS) of all parameters, chain mixing, and convergence to a stationary distribution. The post burn-in sample showed convergence to stationary distribution, good mixing, and high ESS for all parameters (ESS > 200). The maximum clade credibility tree for each order was extracted from the corresponding post-burn-in sample. These complete order-level topologies were then used to replace the corresponding order level sub-trees in the dated molecular trees to produce the final ultrametric topologies that included all described angiosperm genera. This divide and conquer strategy follows the approach of^28^ and results in greatly improved ESS and faster BEAST analyses when compared to analyses of the tree as a whole.

To evaluate the consistency of the resulting diversification analysis, we repeated all analyses using all global and all molecular trees. These analyses produced highly similar patterns (See supplementary figures for details). In addition, we compared the estimates of evolutionary rates using different age constraints for the crown of angiosperms on the molecular and global phylogeny (Fig. S28). Overall, constraining the crown age of angiosperms to 140–210 Ma and 149–256 Ma resulted in somewhat older age estimates for genera compared to that when the constraint was 140–150 Ma. These findings apply both to the molecular tree and global tree.

Our approach for building the phylogeny is similar to the one in a recent study of world floristic regionalization^72^. Compared with ref. ^72^, we used an updated and greatly expanded set of fossils in our dating analysis and our tree includes ca 2000 additional genera. The global phylogeny with the angiosperm crown age constraint of 140–150 Ma along with associated metadata was then used to generate an interactive website that allows the user to explore the topology and access information about taxa, divergence times, and diversification rates. This website was generated using the OneZoom tool^73^ and is available at https://en.geodata.pku.edu.cn/index.php?c=content&a=list&catid=200.

### Global distributions of seed plant genera

#### Geographical standard units

The geographic standard units used in the database is an updated version of refs. ^74,75^, which uses the World Geographical Scheme for Recording Plant Distributions (WGSRPD) and administrative boundaries from the Global Administrative Areas (GADM) database version 1 (http://www.gadm.org/) as base maps. WGSRPD was developed by the Biodiversity Information Standards, formerly Taxonomic Databases Working Group (http://www.kew.org/gis/tdwg/index.html). The aim of WGSRPD is to provide a standard database of geographic names so that the data could be exchanged efficiently across databases without any loss of information^76^. Currently, WGSPRD were widely used to record species distribution, e.g. World Checklist of Selected Plant Families (WCSP, http://apps.kew.org/wcsp/home.do) and the database of Plants of the World Online (http://www.plantsoftheworldonline.org/). GADM provides maps for all countries and their subdivisions and offers the possibility to map species distribution according to the collection localities. However, the sizes of the geographical units in the WGSRPD and GADM vary significantly across space. Therefore, we established our geographical standard units (GSU) for the earth landmasses by (1) merging small adjacent regions of WGSRPD and GADM into larger ones and (2) splitting the large units of WGSRPD to small ones based on GADM to reduce the effects of distribution data deficiency and area on the estimation of genus richness. The final GSUs classified the earth landmasses (islands and Antarctica not included) into 403 geographical units. We then prepared a dictionary of geographical names to link the names of administrative units at different levels (e.g. county, province, country) within GADM and WGSRPD to the names of our GSUs. We standardized and georeferenced the recorded geographical names from different data sources based on the global geographical names database (GeoNames, http://www.geonames.org/).

The maps of our GSUs were prepared using Goode projection (Land) in ArcGIS 10. The areas of all GSUs are roughly standardized with area ranging from 37,923 km² to 2,151,791 km² (Fig. S7A). The mean area of all GSUs is 329,670 km² with a standard deviation of 198,191 km². Linear regression showed that the area of GSU has a non-significant relationship with latitude and can capture the global latitudinal gradient of environmental conditions (Fig. S7B–D). Hence the potential bias of GSU area on species richness is avoided.

#### Compilation of distributional data

We compiled distribution data for global seed plant species from >1100 available data sources, including regional and local floras, online databases of specimens and species, and published checklists and papers in different regions following the approach of ref. ^72^ as outlined below. See Supplementary Data 1 for a detailed list of all data sources used for the compilation of species distribution data.

For different continents and large regions, we compiled data from published regional and continental databases and floras. For example, the distribution records from the former Soviet Union came from the Flora of USSR, which includes distribution data for over 7000 native species. Distribution data for Chinese species were extracted from the Flora of China (both Chinese and English versions) (http://www.efloras.org/; http://frps.iplant.cn/), and Catalogue of Chinese Higher Plants, which include over 200,000 province-level distribution records for over 34,000 species. For India, we used the data from the online Angiosperm Flora of India (beta version, http://flora.indianbiodiversity.org/), which includes the information of over 20,000 species and distribution maps of over 12,000 species. For Europe, we used the data from Euro+Med PlantBase (http://www.emplantbase.org/home.html) that contains the distribution records of over 95% of the European vascular plants. The distribution data for North America was compiled from the Plant Database of US Department of Agriculture (https://plants.usda.gov/java/) and the Database of Vascular Plants of Canada (VASCAN, http://data.canadensys.net/vascan/search/), which contain over 300,000 state-level distribution records for over 31,000 species in USA and Canada. Distribution data for Australia was obtained from the Australian Plant Census (APC, https://www.anbg.gov.au/chah/apc/) and the Census of South Australian Plants, Algae and Fungi (http://www.flora.sa.gov.au/census.shtml). Species distribution data for Brazil was supplemented using the Catalogue of Plants and Fungi of Brazil that includes ca. 35,000 higher plants and over 130,000 state-level distribution records. For Africa, the African Plant Database (http://www.ville-ge.ch/musinfo/bd/cjb/africa/recherche.php) that includes information for over 70,000 species and distribution maps of over 55,000 species. We also compiled distribution data from floras covering smaller scales, for example, the local floras on Russian floras in different regions, and the floras published on eFloras (http://www.efloras.org/). These published databases and floras provided relatively reliable distribution data of species at different spatial scales.

We compiled the global distribution records of species (or genera) from well recognized and authorized datasets at a global scale, e.g. World Checklist of Selected Plant Families (WCSP, http://apps.kew.org/wcsp/home.do), which collects global species distribution data and at the time of our search included information for 173 plant families. We supplemented these records further by adding the distributions of all legume species from the Internal Legume Database & Information Service (ILDIS, http://ildis.org/). We also compiled species distribution data from Tropicos (http://www.tropicos.org/ProjectList.aspx), and online databases and checklists published or maintained by plant research institutes or governments, e.g. Royal Botanic Garden Edinburgh, Smithsonian Tropical Research Institute, British Natural History Museum, Kunming Institute of Botany Chinese Academy of Sciences and Institute of Botany Chinese Academy of Sciences. For example, Bolivia Catalogue compiled by Tropicos includes over 10,000 species and over 50,000 state-level distribution records. These databases have been regularly updated and maintained and contain the latest and relatively reliable data for spatial distributions of plant species.

In addition to these datasets, we also used occurrence data from herbarium specimens, personal collections, and online checklists, some of which have not been scrutinized by taxonomic experts to the same standards. Therefore, these records were used with caution. To improve the quality of species distribution data, we conducted a strict quality control process (see Quality control of the distributional data).

Depending on the types of the raw data on species distributions, we used different methods to reduce spatial conflicts and to improve the accuracy of species distributions in the final dataset. We classified the raw distributional data into four types: coordinates, range maps, gridded distributions, and recorded localities. For species distribution data recorded as coordinates, we first removed the spurious records with latitudinal values outside the range of −90 to 90 and longitudinal values outside the range of −180 to 180. Then, we used the MATLAB function ‘inpolygon’ to map the coordinates to GSUs and retained only those coordinates which were inside the GSUs. To improve the data accuracy, when the coordinates in the herbarium specimen conflicted with the described localities, we used collection localities rather than coordinates to map the taxa to GSUs. For species distributions recorded as range maps, we manually extracted the range map of each taxon using ArcGIS 10 and used the boundaries of the original datasets wherever possible. For species distributions recorded as grid cells, we overlapped these grid cells with the GSUs. Only when the intersected area of a grid cell by GSUs was larger than half of its size, the record of this grid cell was kept. For species distribution data recorded as locality names, all locality names were first searched in the global geographical names service (http://www.geonames.org/) and then were standardized by our geographical names dictionary to make it consistent with the GSUs. When the boundary of a locality did not completely overlap with the GSU boundaries, we intersected its boundary with the GSUs and assigned the locality to the corresponding GSU that covered at least 80% of its area.

The species distribution data in image format was digitized, georeferenced and converted into GIS shape files. All geographical operations were done in ArcGIS 10. We used MATLAB (2013b) to read and import data into the SQL server database (2008 R2) through the SQL JDBC driver.

#### Quality control of the distributional data

To improve the quality of species distribution data, we conducted the following quality control process. We set a threshold for the number of data sources to retain an occurrence record of a species in a GSU in different regions. For European GSUs, occurrence data corroborated by at least 3 data sources were retained; for GSUs of Australia, China, Madagascar, and North America, the threshold was set to 2 data sources. The entire data was retained for Afghanistan, Central America, South America, Africa, Temperate Asia, and Tropical Asia because of the relative data deficiency in these regions. We did not include distributions of genera in the introduced parts of their range in our database. To identify the introduced genera in each geographical unit, we first collected the information for each species. When all species of the genus are introduced in the geographical unit, we deemed it as an introduced genus. We also removed the genus level introduced record according to Plants of the World Online (POWO, http://www.plantsoftheworldonline.org/ accessed: Aug, 2023).

Finally, we manually checked the distribution maps of each genus. New records were added and dubious records were removed according to the genus description from Flora of North America, Flora of China, Wikispecies (https://en.wikipedia.org/wiki/Wikispecies), and other sources. In total, our final species database includes 360,000 species and 15,500 genera with over 2,180,000 distribution records. Based on this species distribution database, we generated a genus distribution database, which includes 397,403 records for 14,976 angiosperm genera. Of these 13,719 genera are included in our global phylogeny, and 11,798 are included in the phylogenetic tree for genera with molecular data. Differences in the number of genera in the distributional dataset and in the phylogenies are due to lack of detailed distributional data for some taxa. Spatial and latitudinal genera richness patterns are shown in Fig. 3. Figure S7E shows the quality of the available data in different geographical units.

#### Species and genus names

Distributional data sources were checked for nomenclatural issues independently of the molecular data and before the final distributional dataset was compiled. We compiled the distributional data for each seed plant genus by aggregating distribution data of all its species. The taxonomic status and the accepted names of species from all data sources were standardized using the recently updated databases Catalogue of Life (COL, http://www.catalogueoflife.org/col/, accessed: May, 2018) and the plant list (TPL) available at http://www.theplantlist.org/ (accessed: Jan 3, 2015). We first matched all the species names with the accepted names of COL and TPL. The unmatched taxonomic names were basically the synonyms, unresolved names, and misspelt names. Therefore, we thoroughly rechecked species names and synonyms in TPL and replaced them with the corrected/accepted names. For taxonomic names that returned multiple matches from TPL, we selected accepted names with the highest confidence level. However, when they showed the same confidence level, we crosschecked the names manually in the World checklist of selected plant names (http://apps.kew.org/wcsp/) as well as Tropicos (http://www.tropicos.org/). The misspelt taxonomic names were corrected using the Taxonomic Name Resolution Service 4.0 (TNRS, http://tnrs.iplantcollaborative.org/TNRSapp.html, accessed: 18 May 2016). The taxonomic names including ‘aff.’, ‘cf.’, and ‘x’ (representing hybrids or taxa of uncertain identification) were not included. The final data set was then compared to the recently updated databases of Plants of The World online (PTW, http://plantsoftheworldonline.org/, accessed: Aug, 2023). When we found conflicts among these databases, we first followed COL, and then POWO. Taxonomic names that were identified as ‘unresolved’ in both COL and POWO were removed. Finally, we compiled 397,403 unique distributional records for 13,719 genera that were also included in our full genus level flowering plant phylogeny (Supplementary Data 5).

We also collected CAM^42,77–79^ and C4^43,80^ photosynthetic pathway data, and the proportion of woody species for each genus. Due to the difficulty in determining CAM and C4 photosynthetic pathway for each species, we classified genera as CAM or C4 when some of their species were identified as CAM or C4 species. The proportion of woody species of each genus was estimated using a newly generated plant life from database which includes life form information for each species based on online databases, published papers, and floras^10,81^. We defined a genus as woody when the proportion of woody species within the genus was over 60%. Similarly, we defined a genus as herbaceous when the proportion of woody species was <40%. Evolutionary rates of different groups of taxa based on the traits outlined above were compared in the R^82^ package “multcompView” and “rcompanion” using one-tailed Wilcoxon test or pairwise Mann–Whitney *U* tests^83,84^.

### Contemporary climate data

Mean annual temperature (MAT) and mean annual precipitation (MAP) data were downloaded from the WorldClim database (v2.0) at a spatial resolution of 2.5 arc minutes^85^. These climate data are generated from terrestrial climate stations (34,542 stations for mean annual precipitation and 20,268 stations for mean annual temperature) using thin‐plate spline method, in which mean MODIS cloud cover, daytime land surface temperatures, distance to oceanic coast, and elevation are used as covariates^85^. These data have been widely used in previous studies at different scales. Mean values of these two climate variables for each geographical unit were then calculated as the average of all cells of 2.5 × 2.5 arc minutes using Zonal Statistics in ArcGIS 10.1 (ESRI Inc.). Mean annual temperature/precipitation ranges were calculated as the difference between maximum and minimum values within each geographical unit. Mean and range of MAT and coefficient of variation of MAP were used to explore the relationships between the mean net diversification rate per geographical unit (calculated as the average of the current net diversification rates of all tips occurring in each geographical unit) and climate (Figs. 4A–D, S12, and S13).

### Diversification analyses

#### Temporal patterns of seed plant diversification

The assumption that phylogenetic trees can be used to study diversification dynamics trough time using stochastic birth-dead models has recently been subjected to criticism^86^. In their study ref. ^86^ shows that current methods to estimate historical fluctuations of evolutionary rates based on dated phylogenies cannot provide reliable estimates of past rates as, for a given tree, there is an infinite number of diversification scenarios that are equally likely. They suggest that the only metric that current methods can estimate are the rates at present or “tip rates” and suggest using two new metrics - pulled speciation rate and pulled diversification rate. The identifiability issues raised by^86^ are important and have far-reaching implications as all widely used methods to study diversification (as those used here) are affected. A recent evaluation of the relevance and potential impact of identifiability issues for studies of diversification based on dated trees of extant species shows that non-identifiability does not imply that current methods to study diversification cannot be used^87^. Acknowledging the importance of identifiability problems^87^, argue that using hypothesis-driven approaches, implementing priors in Bayesian frameworks, and penalizing for models complexity, limits their impact and allows current diversification methods to be used.

Here our main conclusions are mostly based on estimates of tip rates which are suggested to be less affected by identifiability issues. To study rates through time, we use three different methods, two of which are Bayesian approaches (BAMM and diversification rate models implemented with RevBayes). These Bayesian approaches do not aim at selecting a single best fit model that describes lineages evolutionary history but rather sample the posterior distribution of model space and evaluate models based on their frequency in that posterior distribution.

Although estimates of diversification rates shall be interpreted with caution, given our focus on tip rates, the use of a mixture of analytical approaches and the fact that despite some differences all results support our conclusions, we believe that our methodological approach can assess the patterns of diversification at the scale and with the precision that is necessary to support our conclusions.

To study the speciation and net diversification through time, we used the program Bayesian analyses of macroevolutionary mixtures v2.3.0 (BAMM^88^). BAMM models the evolutionary dynamics of lineages through time by defining distinct macroevolutionary cohorts that share common rates of speciation and extinction and that are separated by other such cohorts because of diversification rate shifts. BAMM can assess diversification rate heterogeneity on highly incomplete and phylogenetically non-random datasets and thus is appropriate for the analysis of our data. Although our molecular dataset has high coverage of seed plant genera, as described above we used the polytomy resolver method to include all genera of seed plants in our analyses. The resulting phylogeny covers all the flowering plant genera. However, at the species level, the tree is highly incomplete, as it only includes a single representative from each known genus. In addition, the level of sampling incompleteness is highly non-random as one species represents much higher proportion of the known diversity of species-poor genera, while this is negligible for species-rich genera containing thousands of species (e.g., *Astragalus*).

To account for the diversity of species within genera, BAMM analyses were run for the full and molecular phylogenies separately. In these runs, we set the fraction of backbone completeness and the sampling fractions for each tip (genus) using the *sampleProbsFilename* parameter in BAMM. This parameter is taken as an input file where the level of sampling incompleteness for each tip and for the backbone topology is specified. For analyses based on the molecular phylogeny, the fraction of backbone completeness was set to 0.725 assuming that the species that belong to the 1792 genera without DNA must have branched out from the backbone portion of the phylogeny. When using the global phylogeny, although we have included all described genera, we followed a conservative assumption, and we used a backbone sampling fraction of 0.97. The sampling incompleteness for each tip was set as the reciprocal of the known species diversity of each genus. Using clade-specific sampling fractions allows accounting better for potential biases introduced by non-random sampling strategies and the non-random distribution of missing tips^89^. These files are also available upon request from the authors.

Before all BAMM analyses, outgroup taxa were pruned and proper priors and MCMC chain settings were selected using the *setBAMMpriors* function in the package BAMMtools and the *chainSwapPercent.R* function respectively^89^. All analyses were run on the Abel cluster at the University of Oslo and the High-performance Computing Platform of Peking University until satisfactory chain mixing and effective sampling size values (ESS) of log-likelihood were achieved. ESS was examined using the R library coda as recommended in ref. ^90^.

Outputs from the diversification analyses in BAMM were processed in the R package BAMMtools v 2.0.6^89^. We used the BAMMtools functions to evaluate the shifts in diversification regimes across the seed plant phylogeny (Fig. 1), to extract tip rates of diversification and to visualize the evolutionary rates dynamics through time (Figs. 2, S1, and S4). To extract tip speciation and net diversification rates from the results of the BAMM analyses we used the *getTipRates* function. BAMM was run using a *segLength* setting = 0.02, thus branches ware split into fragments with a length of 0.2% of the total height of the topology. For each of these 0.2% fractions constant rates are assumed. This discretization of the rates is used in BAMM to speed up computation. Here the 0.02 setting implies that branches are discretized in intervals ranging from 3 Ma (for the tree dated with the 140–150 Ma constraint for the crown angiosperms) up to about 5 Ma (for the tree dated with the 149–256 Ma constraint), thus the tip rates that we estimate here are depended on the recent evolutionary history of genera (in the last 3 Ma to 5 Ma).

While working on the present diversification analyses, the BAMM analytical approach has been subjected to a critique by ref. ^91^. Using simulations, the authors of ref. ^91^ reach to the conclusion that there are two major flaws with the BAMM approach. First, they conclude that the likelihood function used to estimate model parameters is incorrect; and second, they found compound Poisson process prior model to be incoherent. We have carefully considered the arguments of ref. ^91^ as the core of our discussion is based on results from analyses in BAMM, and here we outline our arguments in favor of using BAMM in combination with other analytical approaches. Most of the points raised by ref. ^91^, and specifically the one concerning the Poisson prior, have been addressed by ref. ^92^ and in the BAMM online manual^93^ which includes reanalysis of the data used by^91^. This rebuttal shows that there are some major flaws in the way the author of^91^ used BAMM and demonstrates that the likelihood function of ref. ^91^ is incorrect. Another comment on the ability of the BAMM approach to estimate diversification rates correctly has also become available recently^94^; for a reply see ref. ^95^. Although refs. ^91, 94^ discuss important concerns that are relevant to the usage of BAMM, the rebuttal provided by refs. ^92, 95^, show that BAMM performs as intended.

To further ensure that the use of BAMM here does not lead to biased results we used two alternative approaches to estimate diversification rates through time, RPANDA^96^ and RevBayes 1.0.10^97^. In RPANDA, we fitted four different birth-death models (speciation and extinction constant, speciation constant extinction variable, extinction constant speciation variable, and both speciation and extinction variable) to our data, taking into account the fraction of species not present in our phylogeny. Of these four possibilities, the model where both speciation and extinction were allowed to vary best fit the data (based on AIC scores). Results from BAMM also favor a scenario where both speciation and extinction vary through time. Next, we used the *fit_ env* function to investigate the diversification dynamics of angiosperms in relation to environmental variability (in that case in relation to global temperature). For the temperature, we used the environmental data on average global temperatures through the Cenozoic that is provided by RPANDA (in the InfTemp dataset distributed with the package) and extended it to include the time interval back to the origin of crown angiosperms (depending on the dating analyses either 150, 210, or 260 Ma). The data of global historical temperature in the InfTemp dataset has been reconstructed using the delta-O-18 measurements^98,99^, and is available for free. Additional historical temperature data for periods before the Cenozoic were extracted from ref. ^24^, and were estimated with δ^18^O measurements with corrections for water pH effects (see ref. ^24^ for more details). Although the temporal resolution and precision of deep-time temperature data are not as high as information for recent geological periods, it is indicative for the general trends in global temperature fluctuation. Results of these environmental dependency diversification analyses show general trends that are very similar to those obtained by BAMM and are presented in Fig. S2.

In RevBayes we set analyses using the episodic birth-death model^100^ following the instruction in the RevBayes manual. This model allows rates to change between intervals in which they are treated as constant. RevBayes analyses were run until ESS of estimated model parameters exceeded 200 after burn-in. Results were then processed with the R package RevGadgets v 1.0.0. (Fig. S3).

In addition, we compared the inferred diversification dynamics of Silvestro et al.^16^ based on fossils with our results. In their study Silvestro et al.^16^ used a different methodological approach and an independent data set based on the fossil record and found patterns of diversification dynamics that are very consistent with our BAMM, RPANDA and RevBayes results. The convergence of results from BAMM, RPANDA, RevBayes and fossil-based analyses is an additional indication that our results represent a real phenomenon and not an analytical artefact. Thus, we used BAMM to generate the results in the main text and figures, as it is more flexible than RPANDA and RevBayes.

We also compared the estimated evolutionary rates based on the three dating schemes, and we found that these are also very similar (Fig. S28).

#### Spatial patterns of generic diversity and evolutionary dynamics

To calculate spatial patterns of angiosperm generic diversity (Fig. 3), we summed the total number of genera in each GSU. We mapped the spatial patterns of angiosperm evolutionary dynamics in terms of average genus age, speciation, and net diversification of all genera within GSUs.

We calculated the mean values of genus age, speciation rate and net diversification rate for all genera in each GSU (Figs. 3, S8, and S9). To demonstrate the latitudinal gradients of these variables, we used the ‘lowess’ function to plot the generic diversity, mean genus age and mean evolutionary rates per geographical unit against the latitude of the geometric center of the geographical units (Figs. 3 and S10). The ‘lowess’ regression line was generated with the ‘smooth’ function implemented in MATLAB (2017a).

To visually check the spatial patterns of generic diversity according to their evolutionary history, we first assigned all genera to four quartiles by their speciation rate or net diversification rate respectively. For each quartile, we then mapped their relative generic diversity within each geographical unit (Figs. 5 and S20–S24). Spatial variation of absolute and relative generic diversity (proportion) mean evolutionary rates per geographical unit among different age quartiles were also mapped by classifying all genera to four quartiles by their age (Figs. S14–S19).

To explore the correlations between the spatial variation of climate and the spatial variation in mean evolutionary rates per geographical unit, we employed linear regression models using mean net diversification rate per geographical unit as the dependent variable and mean annual temperature or mean annual precipitation as predictors respectively. Reduced major axis regression (i.e. type II regression) was used to explore the correlations between generic richness and mean genus age and mean evolutionary rate because of the potential errors for both dependent variables and predictors (Figs. 4E, F and S26). Linear regressions and reduced major axis regressions were performed with MATLAB v. 2017b using the functions ‘lm.fit’ and ‘lsqfitgm’ (https://www.mbari.org/summary-of-modifications/). We built a null model to test whether diversification rate was randomly distributed across space, assuming that species were randomly distributed across space according to genus richness patterns. We repeated the random process 999 times and used *t*-test to assess whether the observed correlations between the spatial variation of climate and the mean evolutionary rates per geographical unit differed significantly from the null model.

Finally, to evaluate the spatial patterns of rates across latitudes, we investigated the evolutionary dynamics of present-day generic assemblages by dividing the world into 13 latitudinal belts at 10-degree intervals as follows: S5-N5 (the equator), S5-S15, S15-S25, S25-35, S35-S45, S45-S55, N5-N15, N15-N25, N25-N35, N35-N45, N45-N55, N55-N65 and N65-N75. We then estimated the evolutionary rates through time for all the genera distributed in each belt separately (Figs. 2B, S5, and S10). Genera of each latitudinal belt were extracted by aggregating the genera of all GSU within each latitudinal belt. GSU were assigned to a latitudinal belt if >50% of its area fell into a specific belt. Genera that are found in more than one latitudinal belt were included in all respective subsamples. We used the Koppen climate classification system to categorize temperate regions and drylands. In our study, drylands correspond to dry climate (B) while temperate regions correspond to continental region (D) in Koppen climate classification system.

### Reporting summary

Further information on research design is available in the Nature Portfolio Reporting Summary linked to this article.

### Supplementary information

Supplementary Information

Description of Additional Supplementary Files

Supplementary Data 1

Supplementary Data 2

Supplementary Data 3

Supplementary Data 4

Supplementary Data 5

Reporting Summary

## Data Availability

All information needed to evaluate the results and conclusions presented in this study is provided in the manuscript and/or supplementary materials. Phylogenies are publicly available at https://en.geodata.pku.edu.cn/index.php?c=content&a=list&catid=200. Distributional data and diversification rates estimates are provided in Supplementary Data 5. Distribution data was obtained from both on-line databases and directly from the literature and the complete list of distributional data sources is provided in Supplementary Data 1. Species distribution data recorded as locality names were searched in the global geographical names service http://www.geonames.org. Global Administrative Areas boundaries were downloaded from http://www.gadm.org and were used as a base to develop the geographical units used in our spatial analyses. The shape file of the geographical units used in the analyses is included in Supplementary Data 5. Family level evolutionary rate estimates are provided in Supplementary Data 3. All sequences used in the phylogenetic analyses are available in GenBank and accession numbers for all sequences used in the analyses are provided in Supplementary Data 4. Sampling fractions used in the BAMM analyses are available in Supplementary Data 4. The information on fossil calibrations used in the analyses along with a complete list of the relevant references is provided in Supplementary Data 2. The taxonomic status and accepted names of species were standardized using the Catalogue of Life (http://www.catalogueoflife.org/col/, accessed: May, 2018), the plant list (TPL; http://www.theplantlist.org/), World Checklist of Selected Plant Families (http://apps.kew.org/wcsp/) and Tropicos (http://www.tropicos.org/). Misspelled taxonomic names were corrected using the Taxonomic Name Resolution Service 4.0 (TNRS, http://tnrs.iplantcollaborative.org/TNRSapp.html, accessed: May, 2018). The final data set was also compared to the Plant of The World on-line (PTW, http://plantsoftheworldonline.org/, accessed: August, 2023). Climate data was downloaded from the WorldClim database (v2.0) and Chelsea (v2.0) https://chelsa-climate.org/ and climatic data used in the analyses are provided in Supplementary Data 5. Photosynthetic pathway data was collected directly from the literature^77–80^. Growth form data was obtained from the Plant Trait Database https://www.try-db.org/ and from published databases^10^^,^^81^.

## Code availability

During the work on this manuscript, we did not develop custom code, novel computation algorithms, or mathematical approaches. We used publicly available software packages as described in the materials and methods.
