# Supplementary material for: Diversification of flowering plants in space and time
Source: Nat Commun. 2023 Nov 22;14:7609. doi: 10.1038/s41467-023-43396-8 (PMC10665465; doi:10.1038/s41467-023-43396-8)

Supplementary Information for
Diversification of flowering plants in space and time

This pdf includes:
Tables S1 and S2
Figures S1 to S28

Table S1 Variation in mean net diversification rate and mean genus age per geographic unit as a function of mean annual temperature and mean annual precipitation and their ranges. Variation in the log-transformed generic diversity as a function of mean genus age and mean net diversification rate per geographical unit. Mean age and mean net diversification rate per each geographic unit are based on the global phylogeny with angiosperms crown age constrained to 140-150 Ma. See Methods for more details.

Ordinary Least Square (degree of freedom = 401)						
models	variables	Estimate	SE	tStat	pValue	r2
Mean age~Mean annual temperature	Intercept	24.702	0.126	196.343	0.000	0.051
	Mean annual temperature	0.033	0.007	4.626	0.000	
Mean age~Mean annual precipitation	Intercept	24.203	0.113	213.633	0.000	0.224
	Mean annual precipitation	0.001	0.000	10.771	0.000	
Mean net diversification~Mean annual temperature	Intercept	0.069	0.000	269.836	0.000	0.027
	Mean annual temperature	0.000	0.000	-3.338	0.001	
Mean net diversification~Mean annual precipitation	Intercept	0.070	0.000	279.981	0.000	0.077
	Mean annual precipitation	0.000	0.000	-5.800	0.000	
Log(genus richness)~Mean age	Intercept	2.704	0.463	5.842	0.000	0.149
	Mean age	0.154	0.018	8.373	0.000	
Log(genus richness)~Mean net diversification rate	Intercept	11.338	0.635	17.863	0.000	0.124
	Mean net diversification rate	-69.269	9.213	-7.519	0.000	
Mean age~Mean annual temperature range	Intercept	25.288	0.152	166.539	0.000	0.003
	Mean annual temperature range	-0.009	0.009	-1.004	0.316	
Mean age~CV of precipitation	Intercept	25.437	0.130	195.622	0.000	0.018
	CV of precipitation	-0.777	0.288	-2.695	0.007	
Mean net diversification~ Mean annual temperature range	Intercept	0.069	0.000	225.780	0.000	0.006
	Mean annual temperature range	0.000	0.000	-1.514	0.131	
Mean net diversification~ CV of precipitation	Intercept	0.069	0.000	258.988	0.000	0.001
	CV of precipitation	0.000	0.001	0.767	0.443	

Table S2. Variation in mean genus age and mean net diversification rate per geographic unit as a function of the 5%, 25%, 50%, 75% and 95% quartiles of the range of mean annual temperature and mean annual precipitation within geographic unit. Mean age and mean net diversification rate are based on the global phylogeny with angiosperms crown age constrained to 140-150 Ma. Estimated by linear regression.

Model	Estimate	SE	tStat	pValue	r2	dfe
Mean age~5% annual temperature_(Intercept)	24.854	0.102	243.193	0.000		401
Mean age~5% annual temperature_x1	0.003	0.001	4.620	0.000	0.051	401
Mean age~25% annual temperature_(Intercept)	24.757	0.115	214.950	0.000		401
Mean age~25% annual temperature_x1	0.003	0.001	4.732	0.000	0.053	401
Mean age~50% annual temperature_(Intercept)	24.705	0.127	194.791	0.000		401
Mean age~50% annual temperature_x1	0.003	0.001	4.535	0.000	0.049	401
Mean age~75% annual temperature_(Intercept)	24.661	0.137	179.511	0.000		401
Mean age~75% annual temperature_x1	0.003	0.001	4.407	0.000	0.046	401
Mean age~95% annual temperature_(Intercept)	24.591	0.149	164.862	0.000		401
Mean age~95% annual temperature_x1	0.003	0.001	4.464	0.000	0.047	401
Mean age~5% annual precipitation_(Intercept)	24.396	0.107	227.779	0.000		401
Mean age~5% annual precipitation_x1	0.001	0.000	9.639	0.000	0.188	401
Mean age~25% annual precipitation_(Intercept)	24.314	0.110	221.901	0.000		401
Mean age~25% annual precipitation_x1	0.001	0.000	10.160	0.000	0.205	401
Mean age~50% annual precipitation_(Intercept)	24.245	0.112	216.604	0.000		401
Mean age~50% annual precipitation_x1	0.001	0.000	10.544	0.000	0.217	401
Mean age~75% annual precipitation_(Intercept)	24.196	0.114	211.841	0.000		401
Mean age~75% annual precipitation_x1	0.001	0.000	10.720	0.000	0.223	401
Mean age~95% annual precipitation_(Intercept)	24.253	0.115	210.626	0.000		401
Mean age~95% annual precipitation_x1	0.001	0.000	10.041	0.000	0.201	401
Mean net diversification~5% annual temperature_(Intercept)	0.069	0.000	329.906	0.000		401
Mean net diversification~5% annual temperature_x1	0.000	0.000	-2.948	0.003	0.021	401
Mean net diversification~25% annual temperature_(Intercept)	0.069	0.000	293.590	0.000		401
Mean net diversification~25% annual temperature_x1	0.000	0.000	-3.203	0.001	0.025	401
Mean net diversification~50% annual temperature_(Intercept)	0.069	0.000	267.812	0.000		401
Mean net diversification~50% annual temperature_x1	0.000	0.000	-3.287	0.001	0.026	401
Mean net diversification~75% annual temperature_(Intercept)	0.070	0.000	248.190	0.000		401
Mean net diversification~75% annual temperature_x1	0.000	0.000	-3.393	0.001	0.028	401
Mean net diversification~95% annual temperature_(Intercept)	0.070	0.000	229.482	0.000		401
Mean net diversification~95% annual temperature_x1	0.000	0.000	-3.651	0.000	0.032	401
Mean net diversification~5% annual precipitation_(Intercept)	0.070	0.000	299.881	0.000		401
Mean net diversification~5% annual precipitation_x1	0.000	0.000	-5.248	0.000	0.064	401
Mean net diversification~25% annual precipitation_(Intercept)	0.070	0.000	291.093	0.000		401
Mean net diversification~25% annual precipitation_x1	0.000	0.000	-5.424	0.000	0.068	401
Mean net diversification~50% annual precipitation_(Intercept)	0.070	0.000	283.808	0.000		401
Mean net diversification~50% annual precipitation_x1	0.000	0.000	-5.623	0.000	0.073	401

Mean net diversification~75% annual precipitation_(Intercept)	0.070	0.000	278.383	0.000		401
Mean net diversification~75% annual precipitation_x1	0.000	0.000	-5.872	0.000	0.079	401
Mean net diversification~95% annual precipitation_(Intercept)	0.070	0.000	278.085	0.000		401
Mean net diversification~95% annual precipitation_x1	0.000	0.000	-5.447	0.000	0.069	401

Figure S1. Speciation and net diversification through time estimated based on the global and molecular phylogenies with the constraints for the angiosperm crown age being 140-150 Ma, 140-210 Ma and 149-256 Ma. Solid red lines represent mean rate estimates and shaded areas represent the 95% confidence intervals.

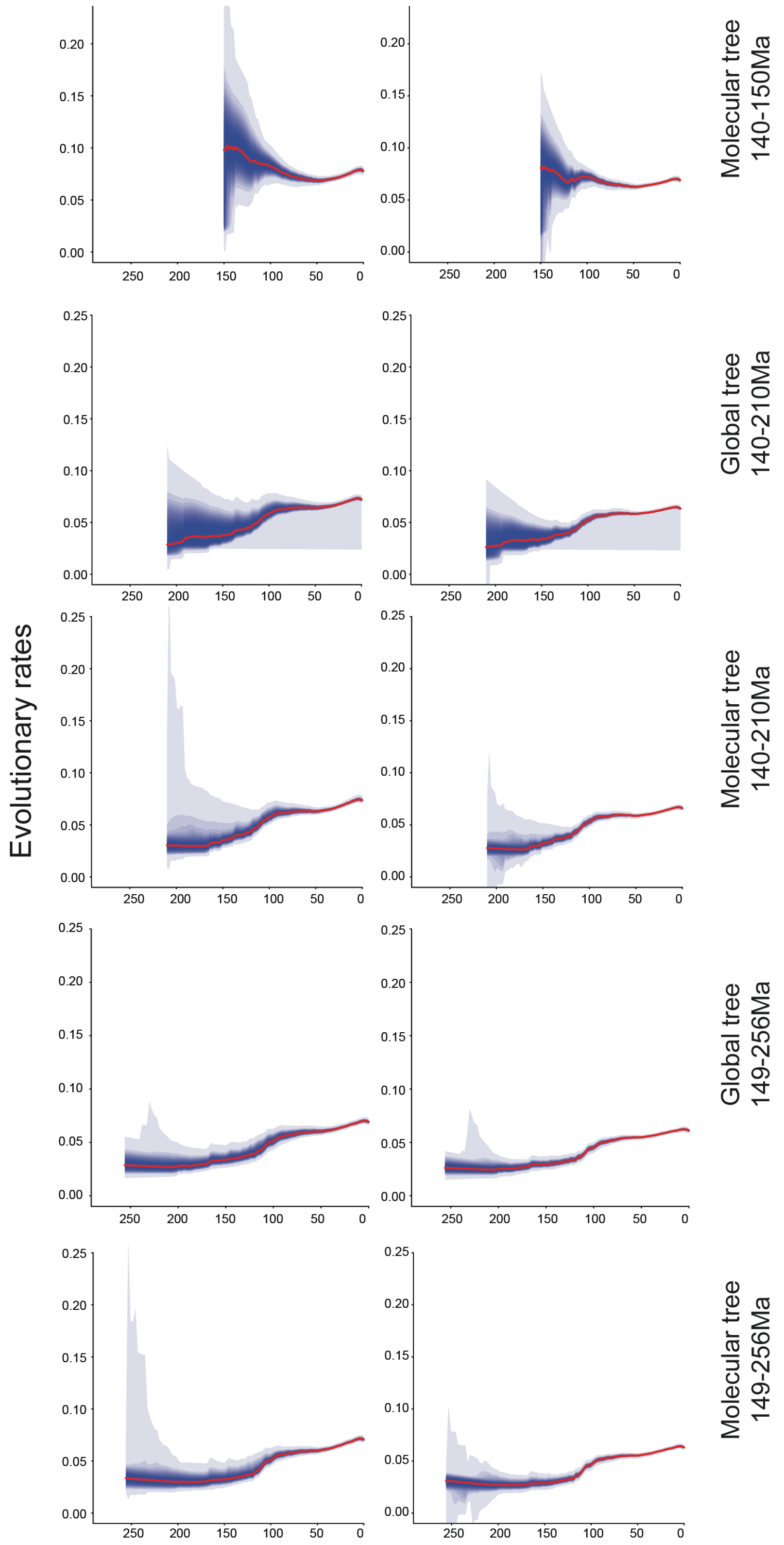

Figure S2. Speciation and net diversification rates estimated by RPANDA. The results about the temporal trends in evolutionary rates based on RPANDA are consistent with those evaluated by BAMM.

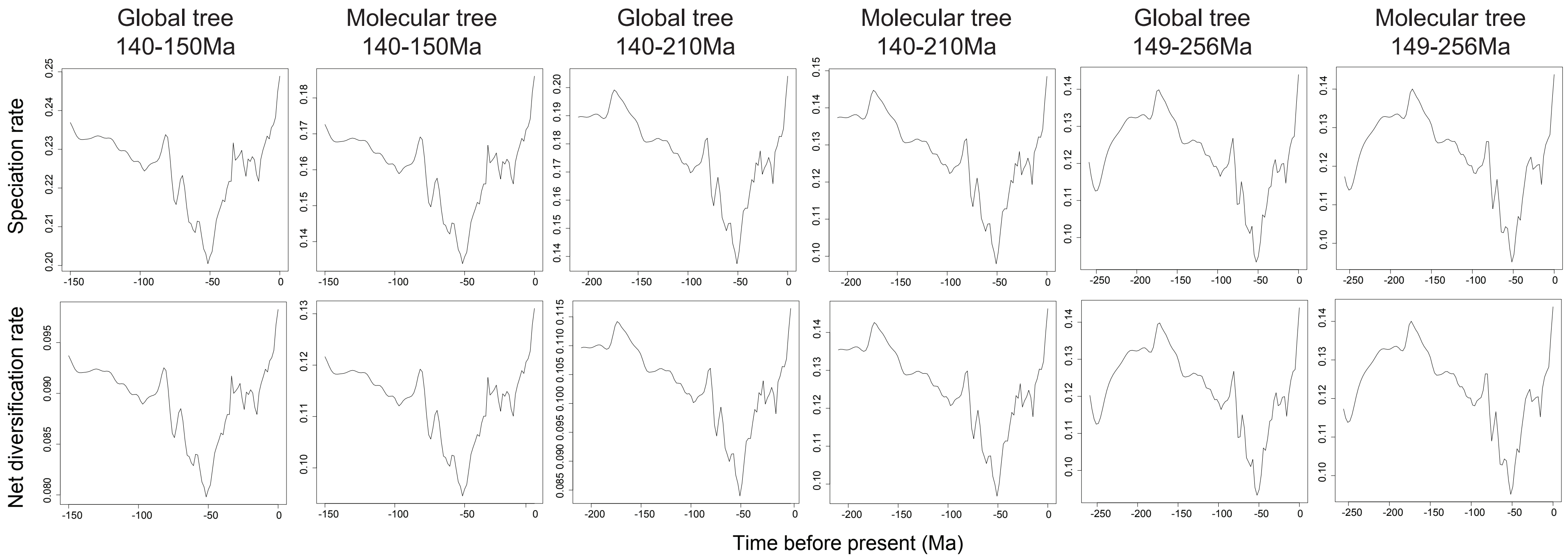

Figure S3 Speciation and net diversification rate using results from the tree dating schemes and the global trees based on the episodic birth-death model in RevBayes. Solid lines represent the mean estimate and colored areas around the 95% confidence interval.

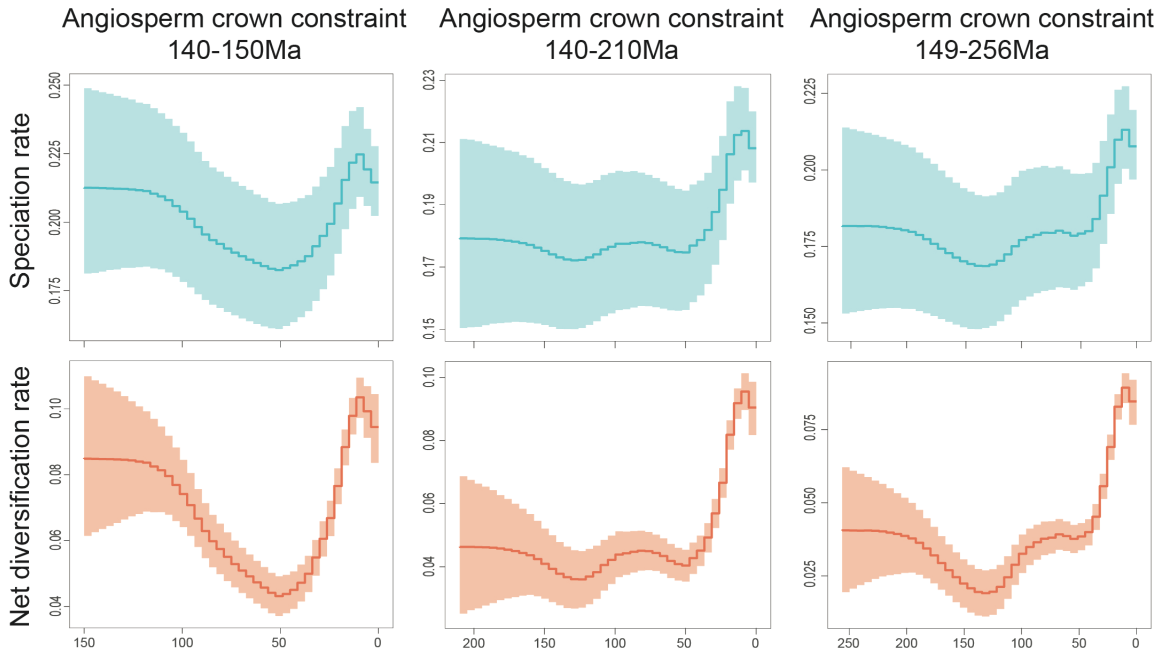

Figure S4. Speciation and net diversification through time estimated based on the molecular phylogenies using only monophyletic genera with the constraints for the angiosperm crown age being 140-150 Ma, 140-210 Ma and 149-256 Ma. Solid red lines represent mean rate estimates and shaded areas represent the 95% confidence intervals.

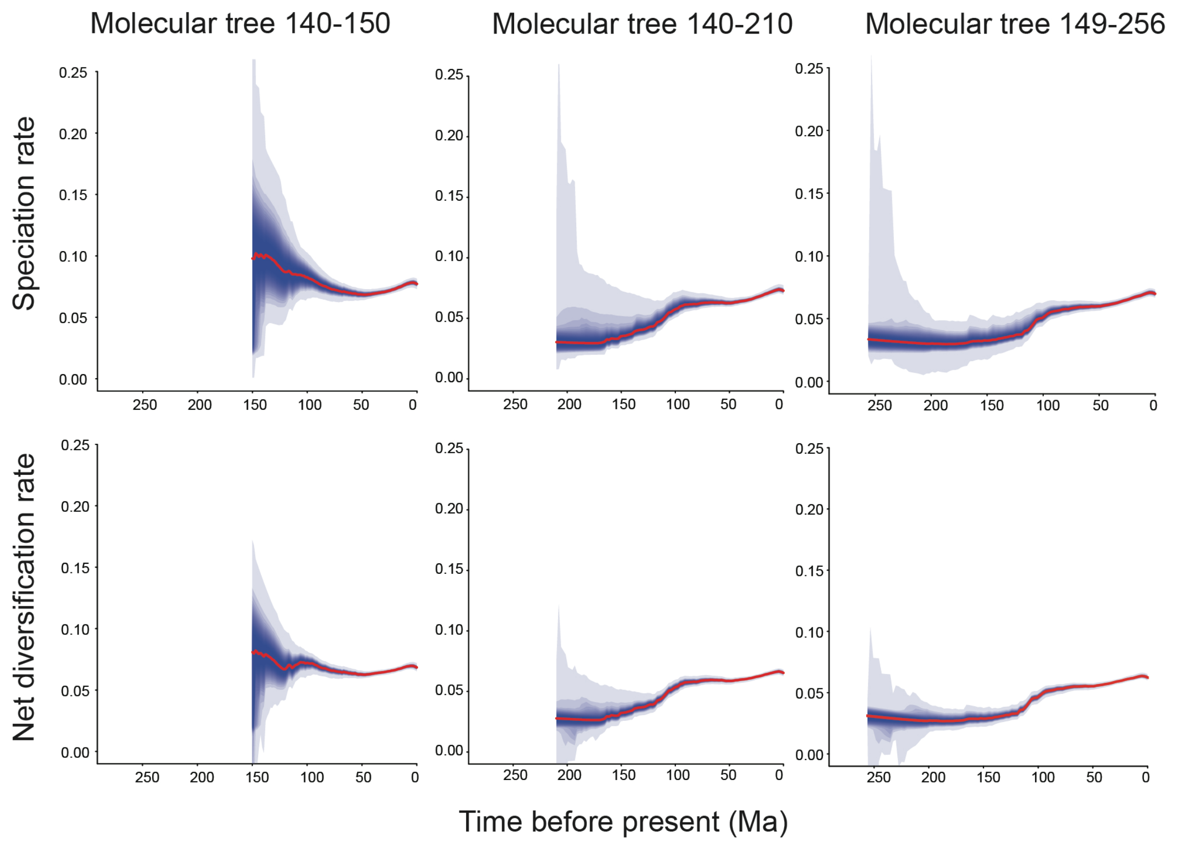

Figure S5. Evolutionary rates through time for flowering plant genera distributed in different latitudinal belts. Evolutionary rates were estimated using the following phylogenies: molecular phylogeny with constrain 140-150 Ma (A), global phylogeny with constrain 140-210 Ma (B), molecular phylogeny with constrain 140-210 Ma (C), global phylogeny with constrain 149-256 Ma (D), molecular phylogeny with constrain 149-256 Ma (E). From top down, the two rows represent speciation rates and net diversification rates. The darker colors indicate higher latitudes, and the red line indicates the Equator belt (-5 ° to 5 °).

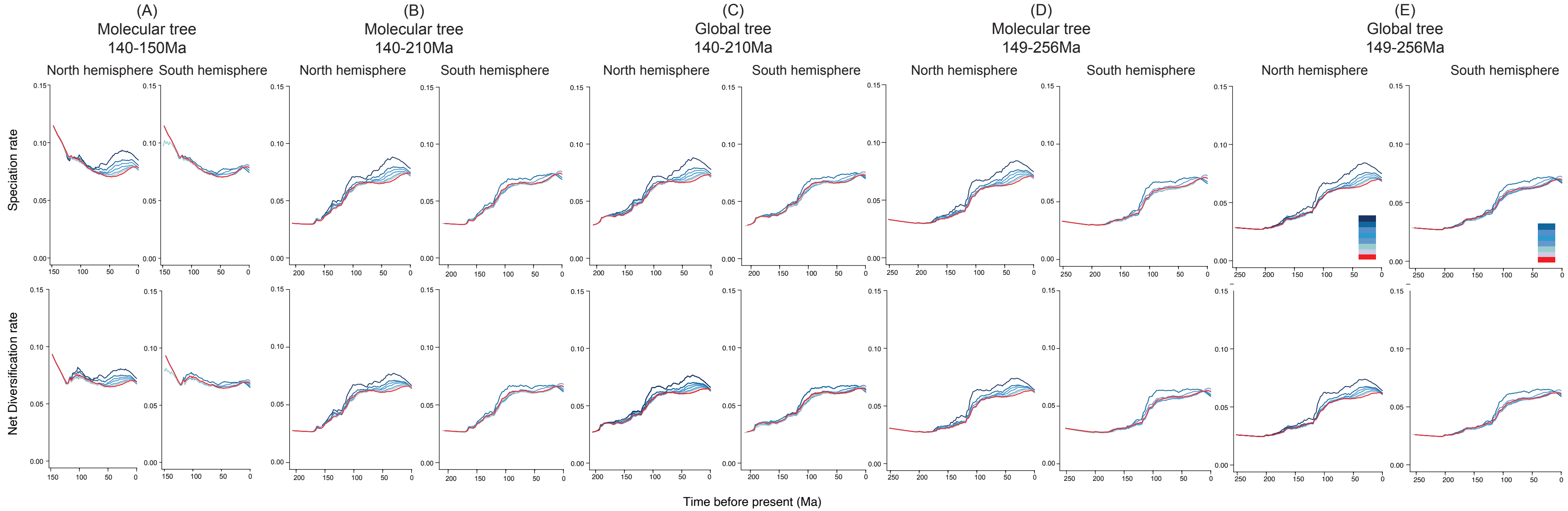

Figure S6. Comparison between speciation and net diversification rates of genera endemic to the temperate regions (*n* = 4921) vs genera endemic to the tropics (*n* = 4366) and genera restricted to deserts and drylands (*n* = 1831) vs genera found only outside arid regions (*n* = 3843) based on the global phylogeny dated using a constrain 140-150 Ma for the crown angiosperms. Wilcoxon rank-sum two-sided test was performed. The white dot shows the median in each violin plot, and the black box illustrate 25th and 75th percentiles, with whiskers extending to the largest value within 1.5-fold the interquartile range.

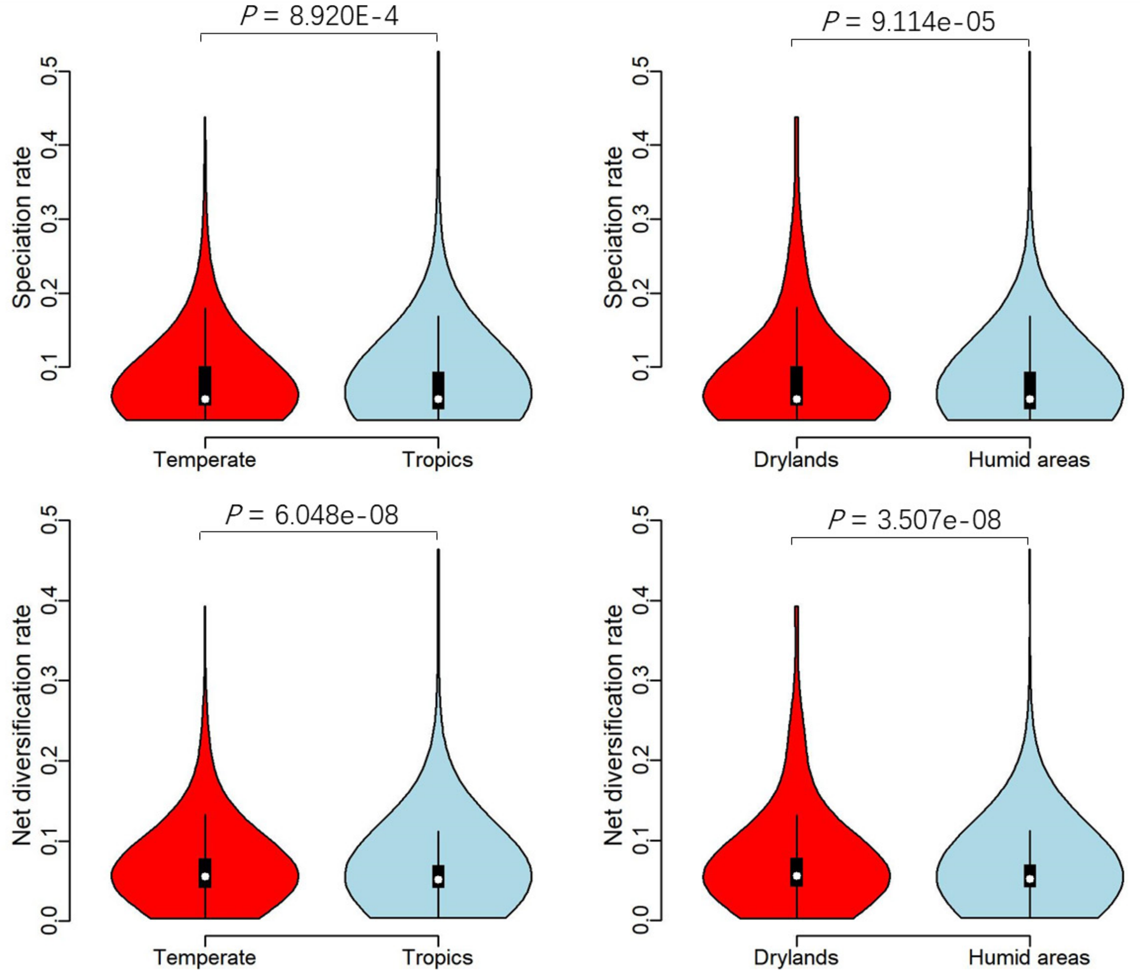

Figure S7 Area frequency distribution (A) and latitudinal trend of the area (B) of the geographical units in the standardized geographical map (SGM) used for the compilation of the angiosperm distribution. The SGM has 403 geographical units excluding islands, mean area per geographical unit is ca. 2 $\times 10^5$ km^2 (3.8 × 10^4 - 2.15 × 10^6 km^2). The area of each geographical unit is insignificantly correlated to latitude, indicating that area will not bias latitudinal trends of generic diversity and evolutionary rates. C and D show he variation of mean annual temperature (C) and precipitation (D) per geographical unit. E shows the quality of the available data in different geographical units. (F) shows the correlation between genus richness and the number of data sources used for the compilation of distribution data within geographical units.

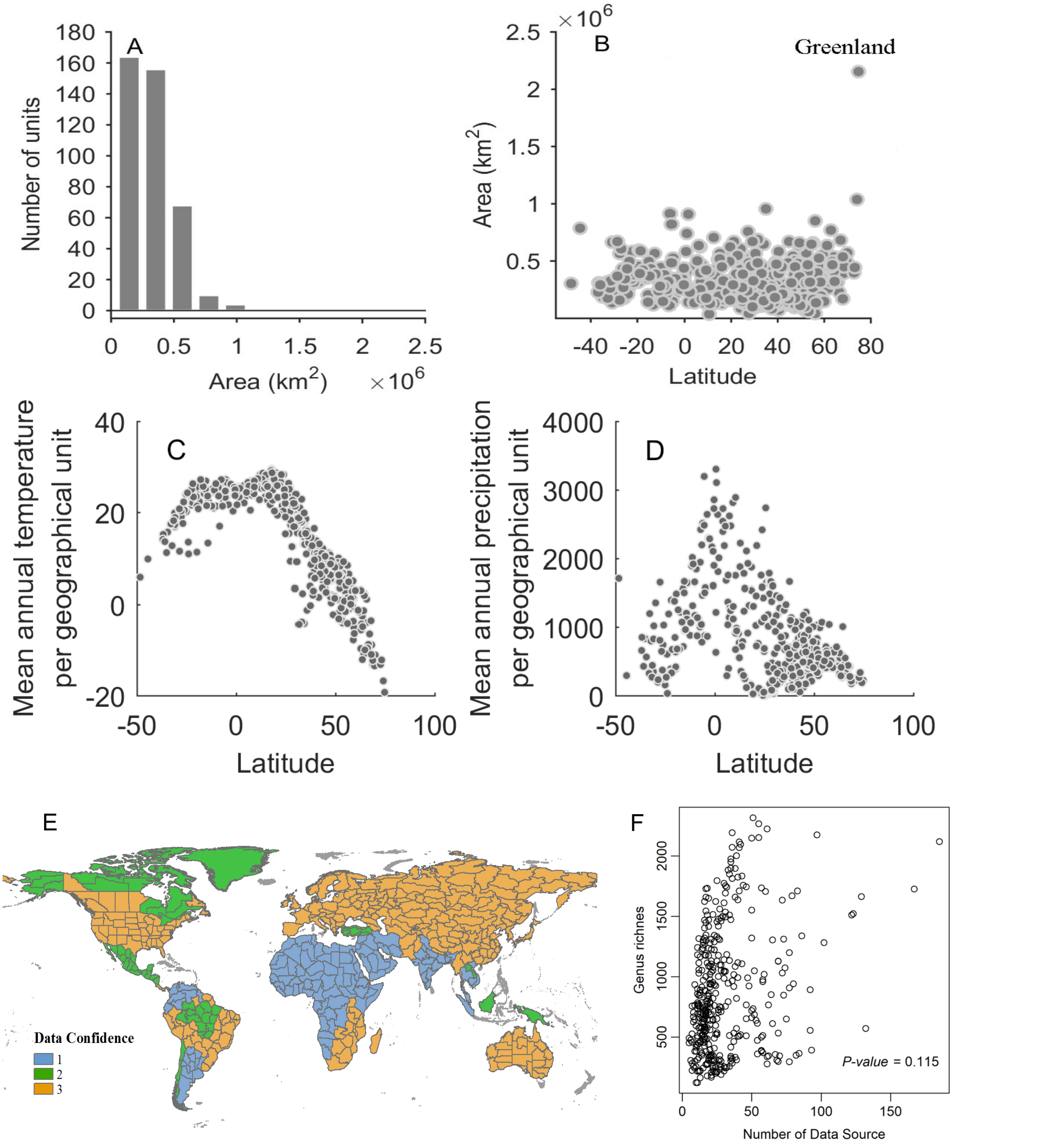

Figure S8. The spatial patterns of mean genus age (first row), mean speciation rate (second row), mean net diversification rate (third row). These analyses were repeated for 1) the molecular phylogeny with the constraint for the crown age of angiosperms to being 140-150 Ma (first column), 2) the global (second column) and 3) molecular (third column) phylogenies with the constraint for the crown age of angiosperms being 140-210 Ma, 2) the global (fourth column) and 3) molecular (fifth column) phylogenies with the constraint for the crown age of angiosperms being 149-256 Ma.

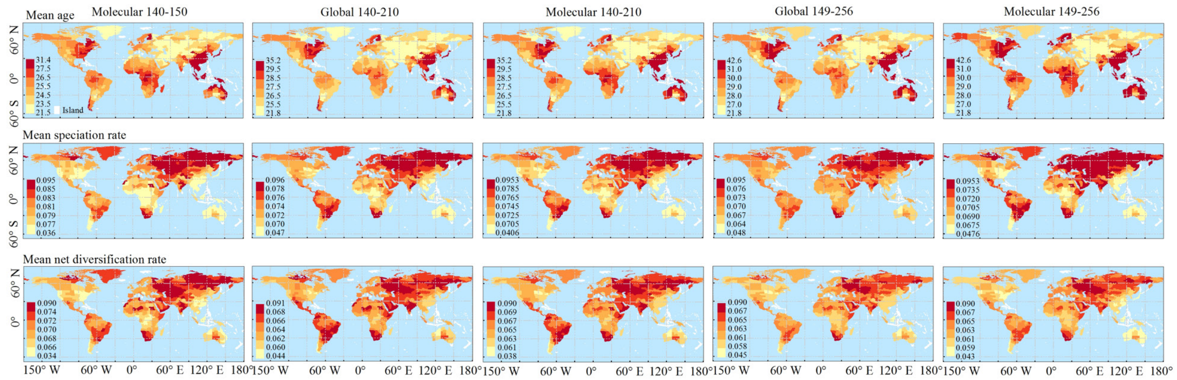

Figure S9. Global patterns of mean values of genus age (first row), speciation rates (second row) and net diversification rates (third row) for each geographic unit. Mean genus age and mean evolutionary rates are estimated for only monophyletic genera using the molecular phylogenies with angiosperms crown age constrained to 140-150 Ma, 140-210 Ma, and 149-256 Ma. Regions with no distributional data are shown in white.

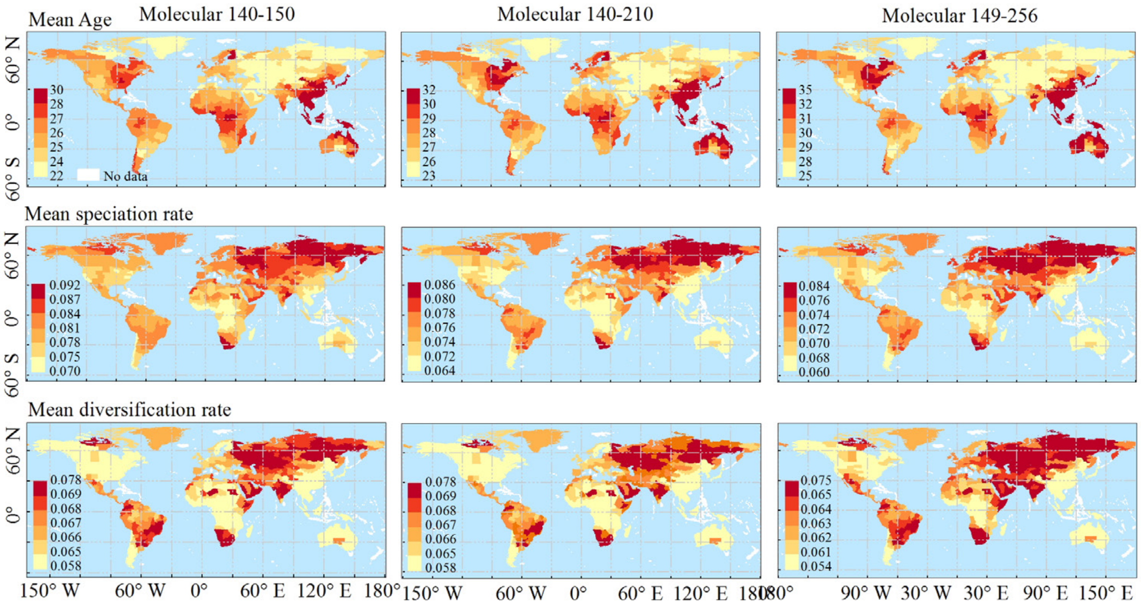

Figure S10. Latitudinal trends in mean values of genus age (the first column), speciation rate (the second column) and diversification rate (the third column) for each geographic unit. These analyses were repeated for 1) molecular (the first row) phylogenies with the constraint for the crown age of angiosperms being 140-150 Ma, 2) the global (the second row) and molecular (the third row) phylogenies with the constraint for the crown age of angiosperms being 140-210 Ma; 3) the global (the fourth row) and molecular (the fifth row) phylogenies with the constraint for the crown age of angiosperms being 149-256 Ma and 5). The speciation rate and net diversification rate show consistent latitudinal gradients. From the equator to the poles, the speciation rate and net diversification rate increase. Individual data point in blue, black lines are drawn using lowess regression with span of 0.5.

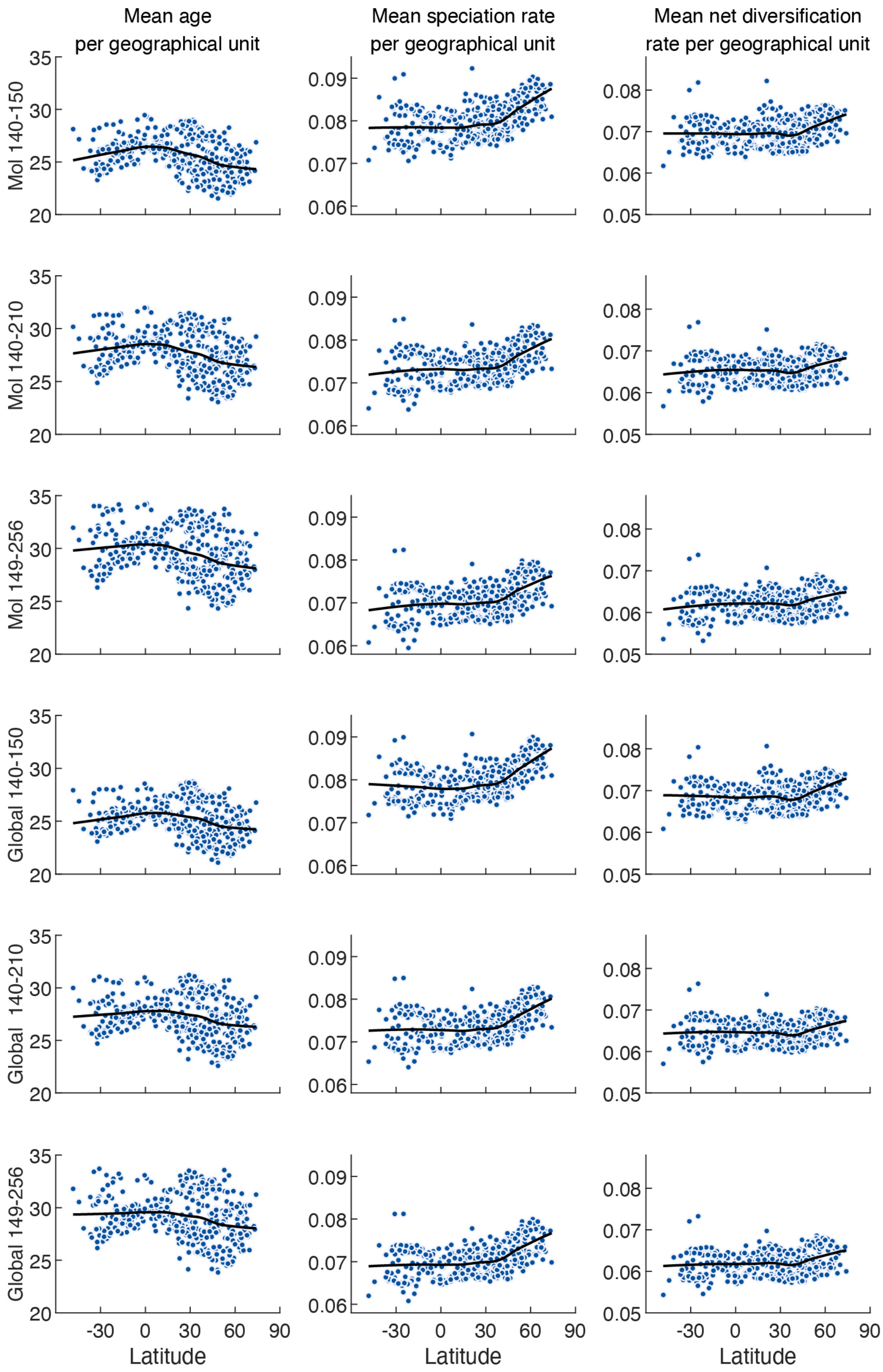

Figure S11. The frequency distribution of slope, P-value (Pr(>|t|)) and adjusted-r2 (adj.r.squared) from the null model for the latitudinal trend of (A), relationship between genus age and latitude; (B) relationship between mean speciation rate and latitude; (C) relationship between mean net diversification rate and latitude; (D) relationship between genus richness and latitude; (E) relationship between genus richness and mean genus age. All results are based on the global tree with crown age of angiosperms constrained to 140-150 Ma. Red arrows show the observed values, which differ significantly from the null model predictions (*t*-test: $\alpha = 0.05$).

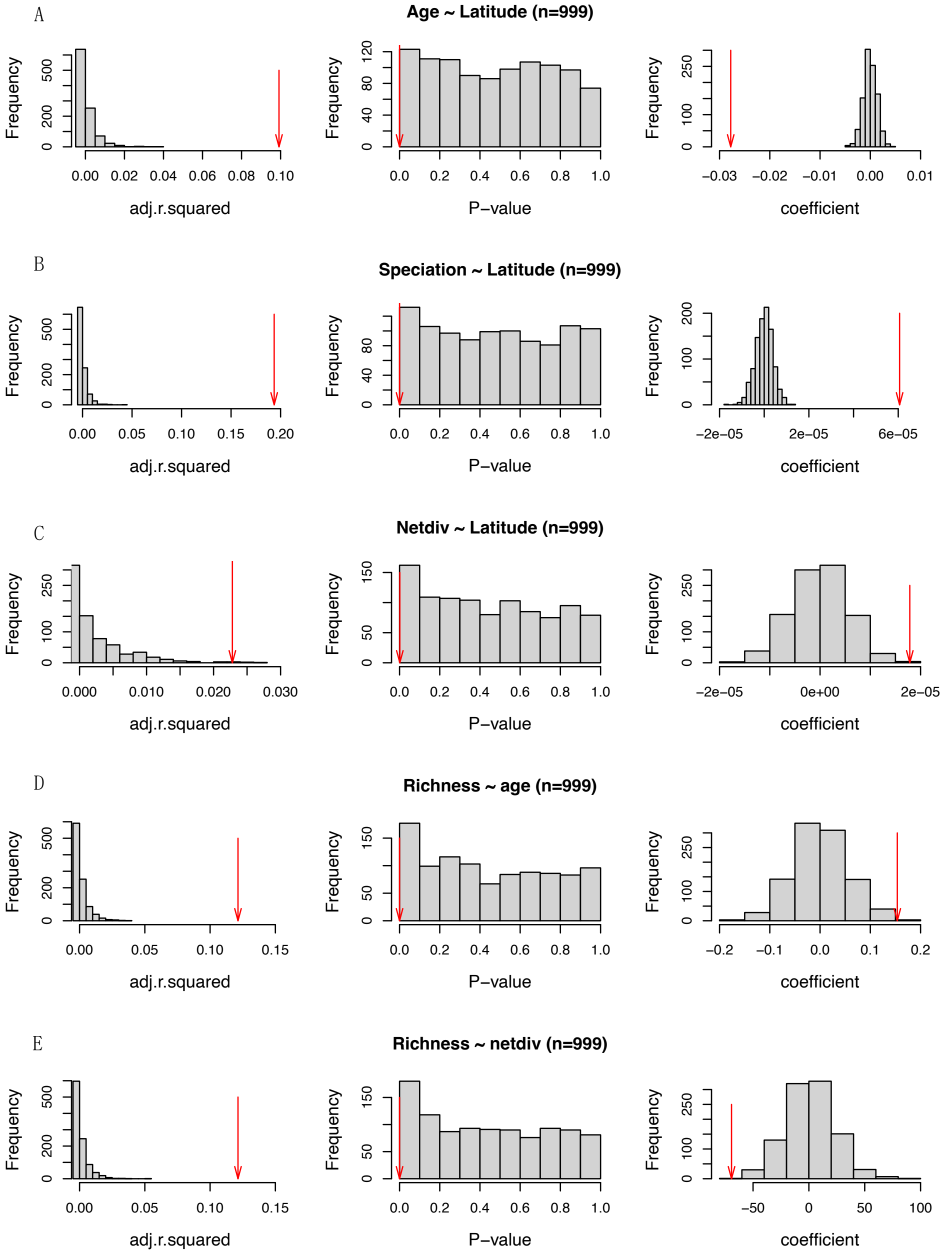

Figure S12. Variation in mean genus age and mean net diversification rate per geographic unit as functions of five quartiles (i.e., 5%, 25%, 50%, 75% and 95%) of the mean annual temperature and mean annual precipitation within geographic units. Mean genus age and mean net diversification rate are estimated using the phylogeny with angiosperm crown age constrained to 140-150 Ma. Solid red lines represent linear regression lines, individual data points are in blue. All *p*-values (*P*) and r^2 presented in the figures were estimated by linear regressions.

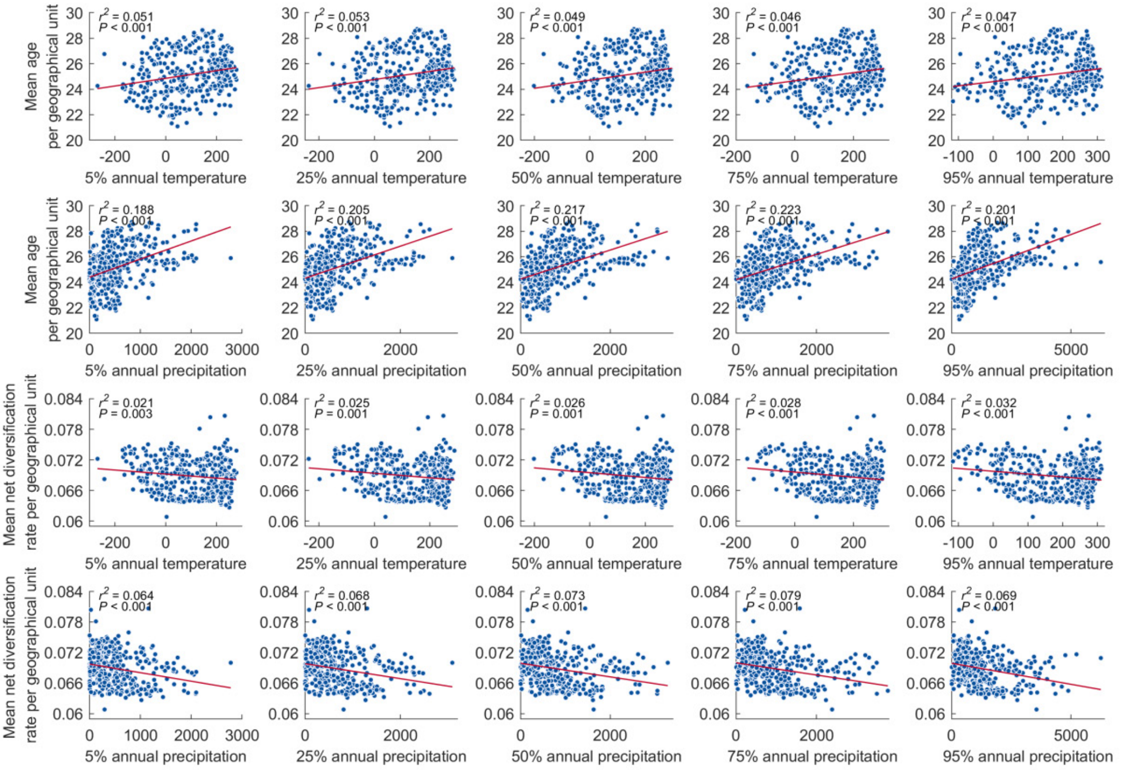

Figure S13. Variation in mean genus age (A, B) and mean net diversification rate (C-D) per geographic unit as functions of the range of mean annual temperature and the coefficient of variation of mean annual precipitation within geographic unit. Mean genus age and mean net diversification rate are based on the phylogeny with the angiosperm crown age constrained to 140-150 Ma. Solid red lines represent the linear regression lines, individual data points are in blue. All *p*-values (*P*) and r^2 presented in the figures were estimated by linear regressions.

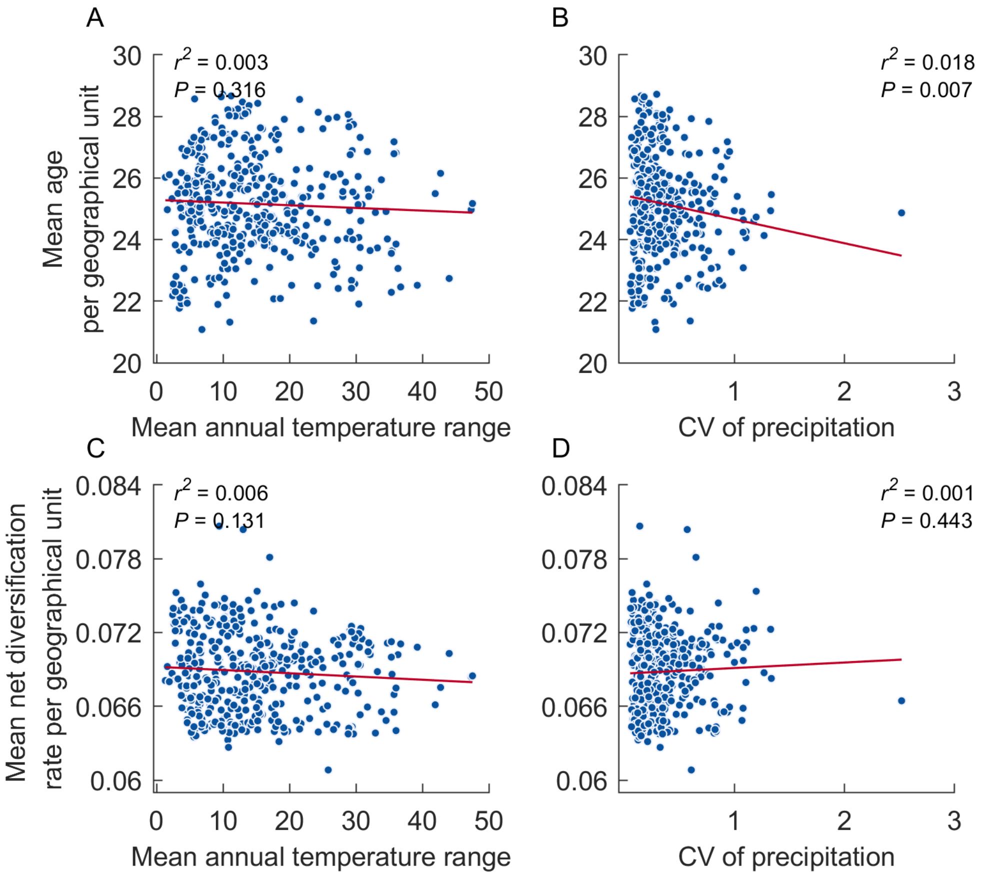

Figure S14. The richness (the first column), proportions (the second column), mean speciation rate (the third column) and net diversification rate (the third column) of angiosperm genera with different ages. The evolutionary rates of genera were estimated using the global phylogeny with the constraint for the crown age of angiosperms being 140-150 Ma. All genera were divided into four quartiles in an increasing order of genus age.

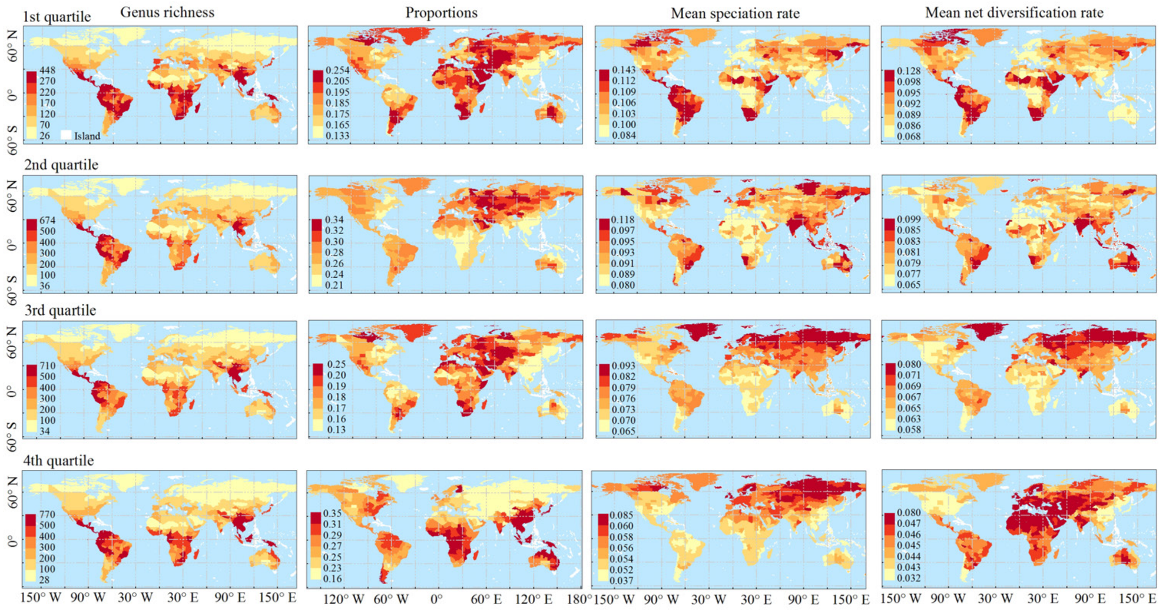

Figure S15. The richness (the first column), proportions (the second column), mean speciation rate (the third column) and net diversification rate (the third column) of angiosperm genera with different ages. The evolutionary rates of genera were estimated using the molecular phylogeny with the constraint for the crown age of angiosperms being 140-150 Ma. All genera were divided into four quartiles in an increasing order of genus age.

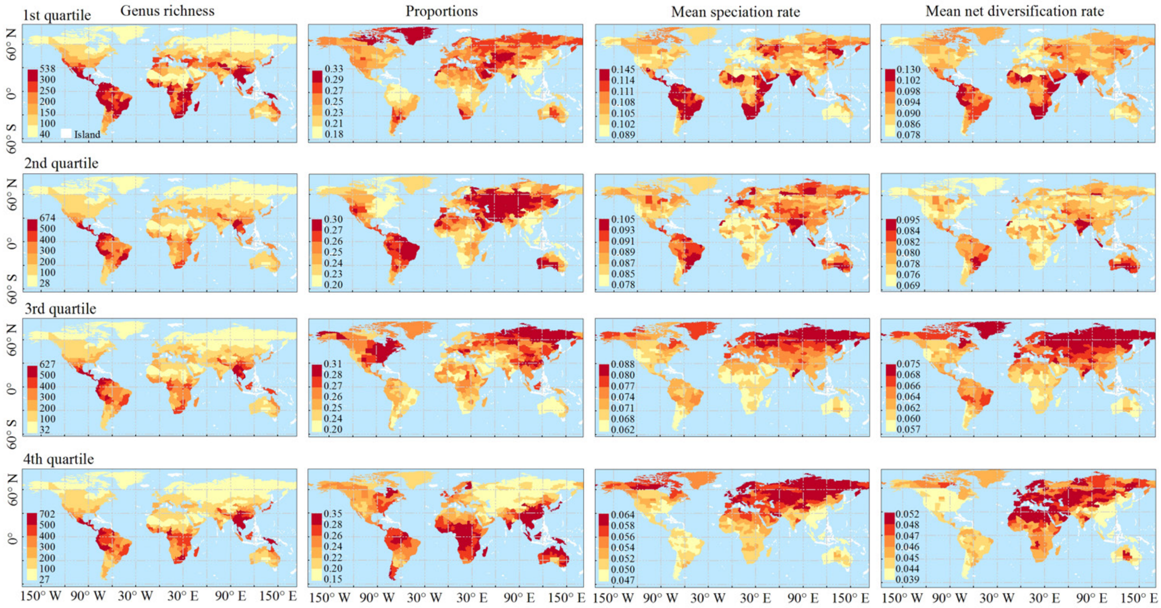

Figure S16. The richness (the first column), proportions (the second column), mean speciation rate (the third column) and net diversification rate (the third column) of angiosperm genera with different ages. The evolutionary rates of genera were estimated using the global phylogeny with the constraint for the crown age of angiosperms being 140-210 Ma. All genera were divided into four quartiles in an increasing order of genus age.

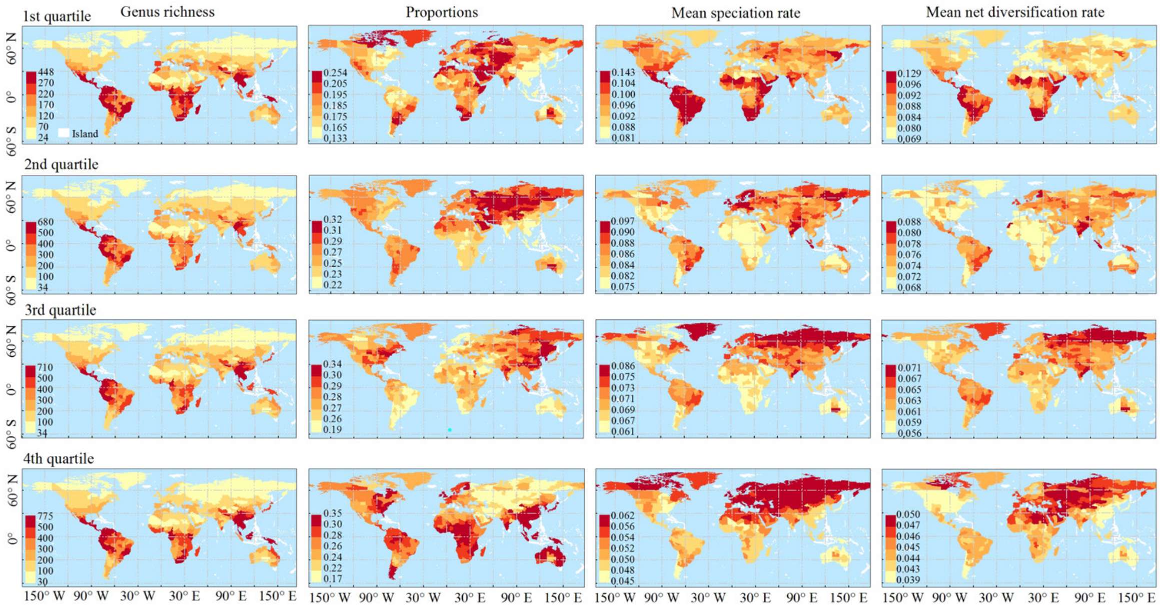

Figure S17. The richness (the first column), proportions (the second column), mean speciation rate (the third column) and net diversification rate (the third column) of angiosperm genera with different ages. The evolutionary rates of genera were estimated using the molecular phylogeny with the constraint for the crown age of angiosperms being 140-210 Ma. All genera were divided into four quartiles in an increasing order of genus age.

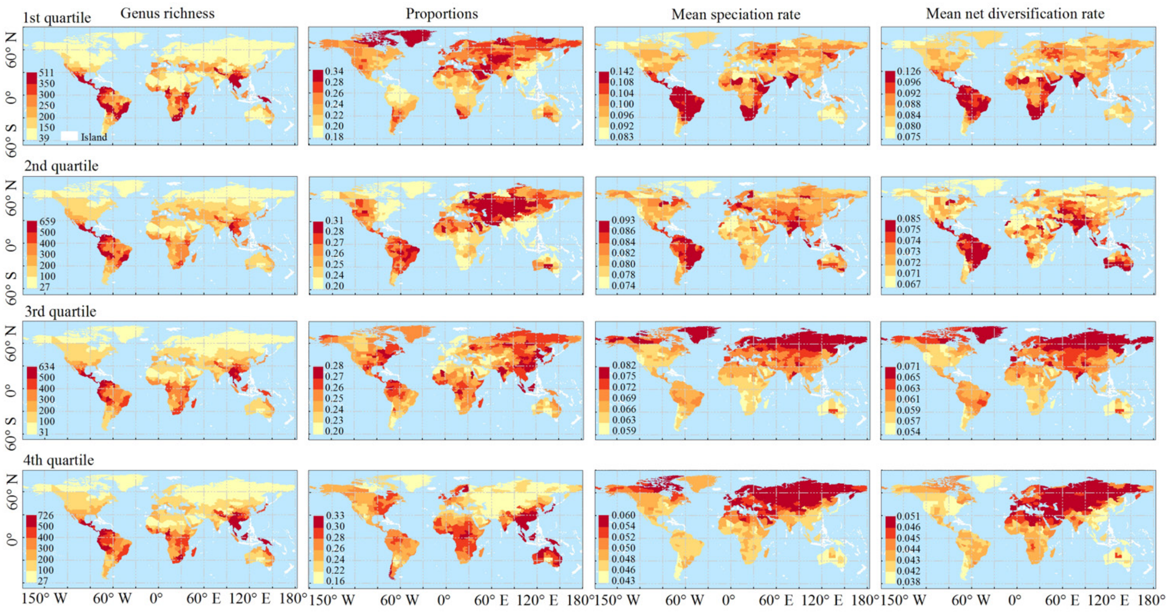

Figure S18. The richness (the first column), proportions (the second column), mean speciation rate (the third column) and net diversification rate (the third column) of angiosperm genera with different ages. The evolutionary rates of genera were estimated using the global phylogeny with the constraint for the crown age of angiosperms being 149-256 Ma. All genera were divided into four quartiles in an increasing order of genus age.

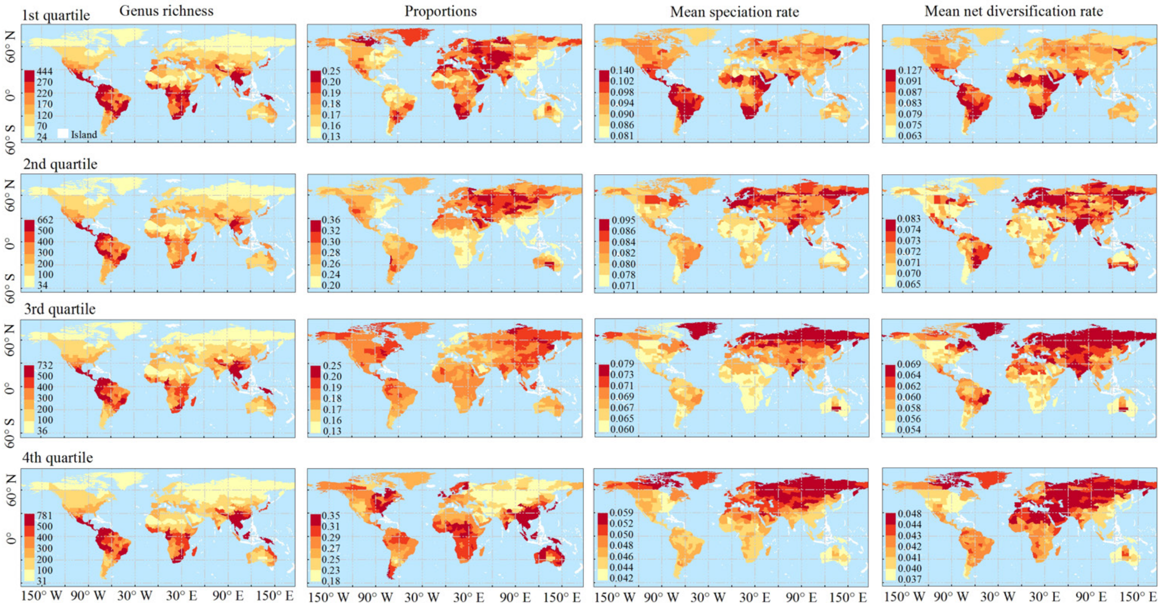

Figure S19. The richness (the first column), proportions (the second column), mean speciation rate (the third column) and net diversification rate (the fourth column) of angiosperm genera with different ages. The evolutionary rates of genera were estimated using the molecular phylogeny with the constraint for the crown age of angiosperms being 149-256 Ma. All genera were divided into four quartiles in an increasing order of genus age.

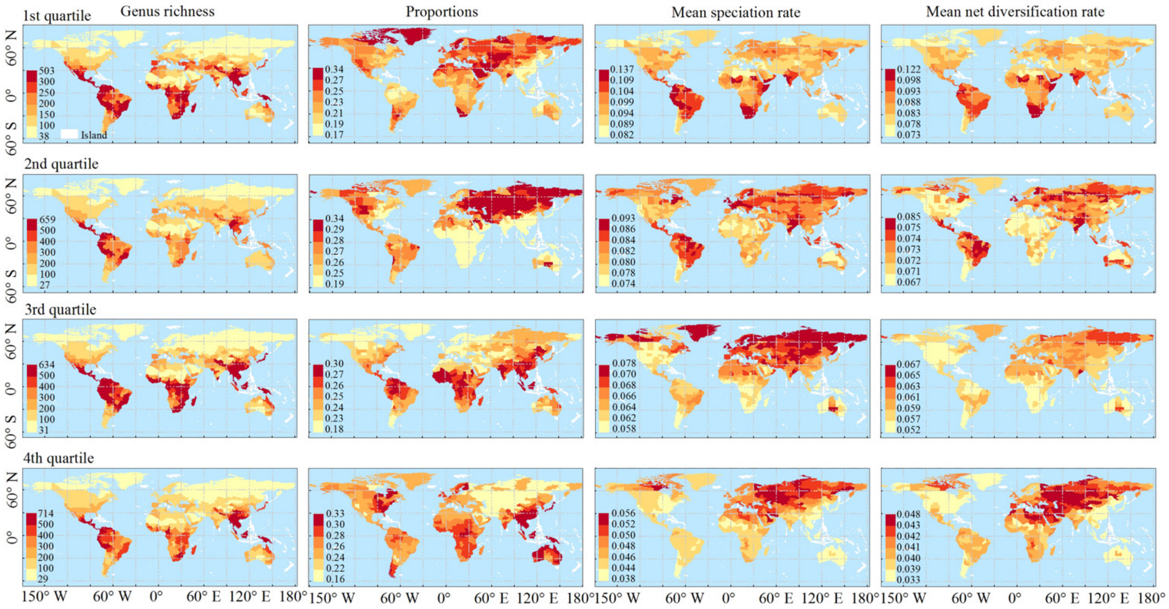

Figure S20. Patterns in the proportions of genera with different evolutionary rates. All genera were divided into four quartiles in an increasing order of their speciation rates (first column) and net diversification rates (second column) separately. From the first to the fourth quartile, the evolutionary rates of genera increase. Then we calculated the proportion of each quartile relative the total generic diversity in each geographical unit. The results show that the proportions of the first quartiles defined by speciation rate and diversification rate are both high in the tropics, while those of the fourth quartiles are both high in temperate regions. The evolutionary rates of each genus were estimated using the molecular phylogeny with the constraint for the crown age of angiosperm to be 140-150 Ma.

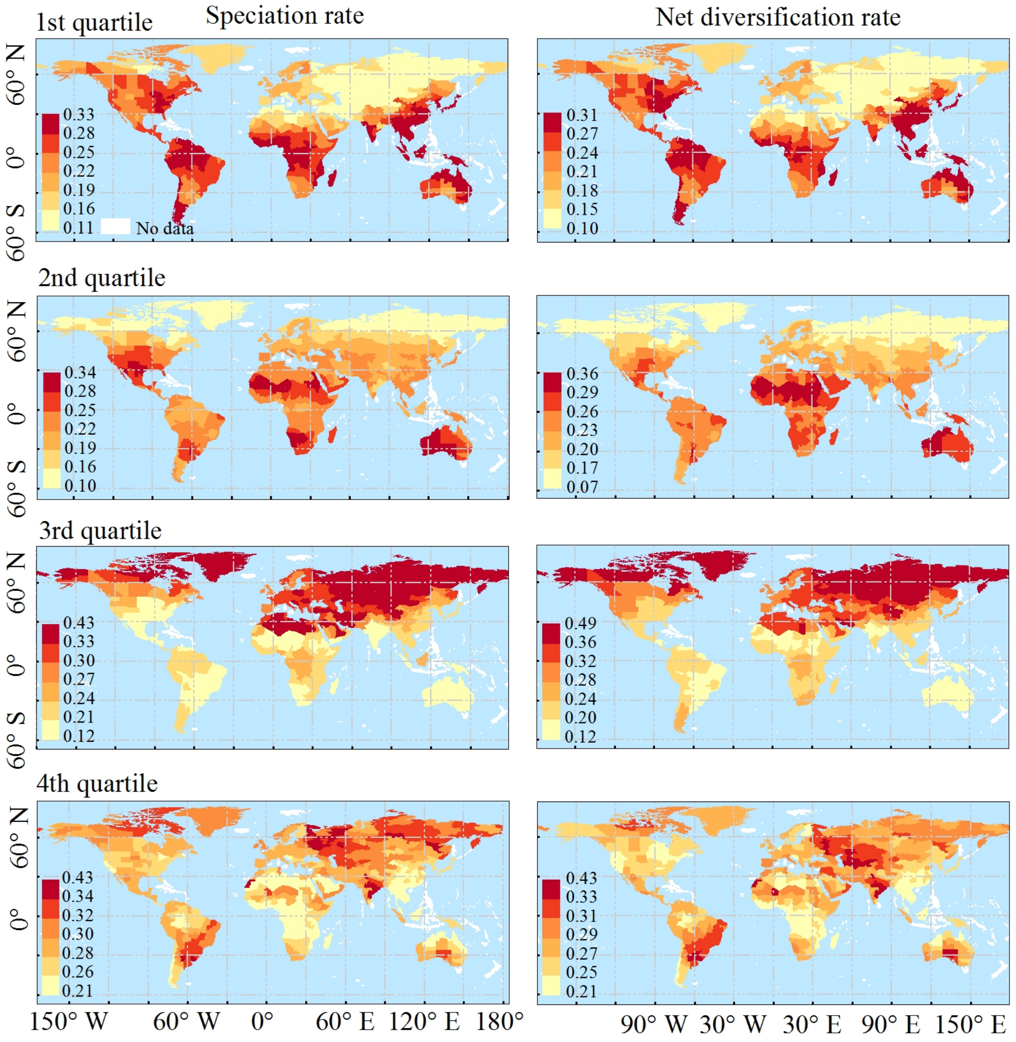

Figure S21. Patterns in the proportions of genera with different evolutionary rates. All genera were divided into four quartiles in an increasing order of their speciation rates (first column) and net diversification rates (second column) separately. See Methods and Figure S18 for details of the division of genus groups. The evolutionary rates of each genus were estimated using the global phylogeny with the constraint for the crown age of angiosperm to be 140-210 Ma.

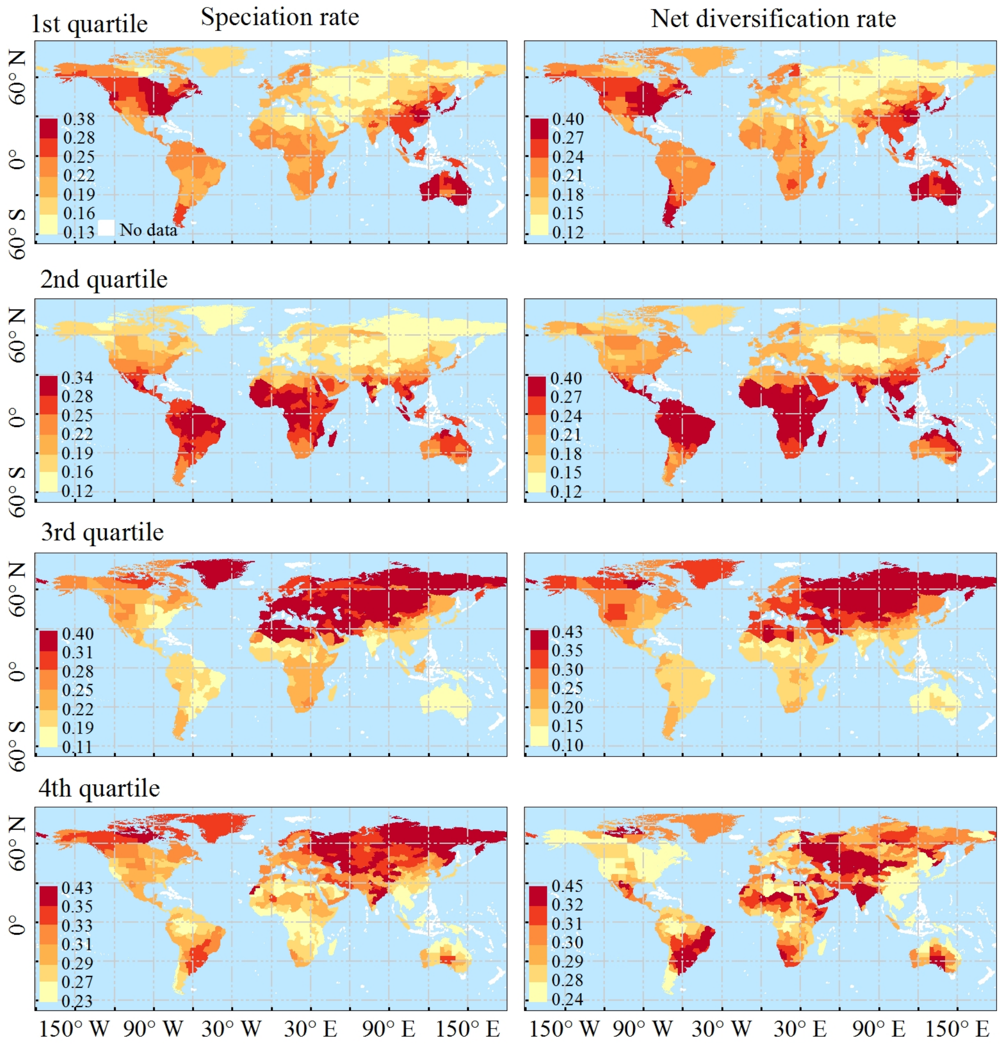

Figure S22. Patterns in the proportions of genera with different evolutionary rates. All genera were divided into four quartiles in an increasing order of their speciation rates (first column) and net diversification rates (second column) separately. See Methods and Figure S18 for details of the division of genus groups. The evolutionary rates of each genus were estimated using the molecular phylogeny with the constraint for the crown age of angiosperm to be 140-210 Ma.

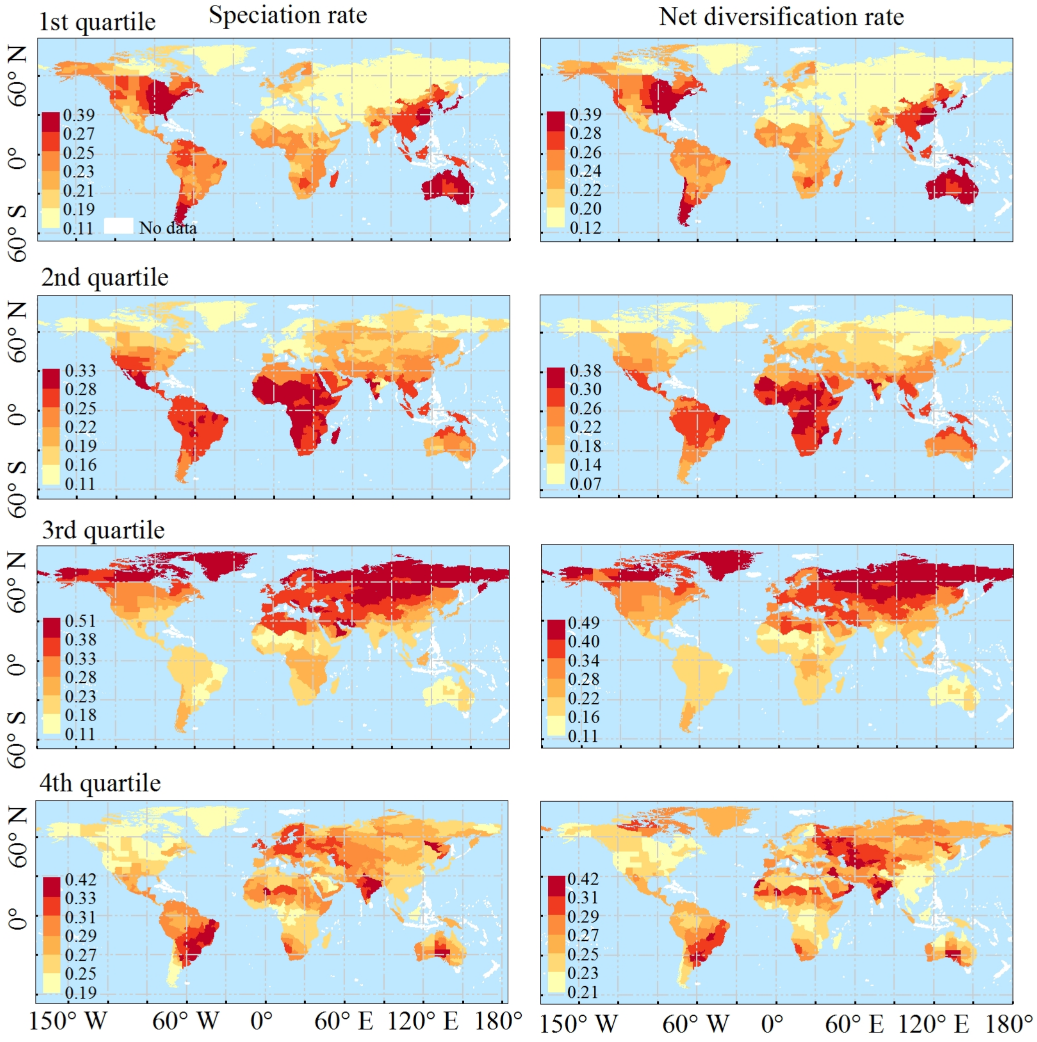

.

Figure S23. Patterns in the proportions of genera with different evolutionary rates. All genera were divided into four quartiles in an increasing order of their speciation rates (first column) and net diversification rates (second column) separately. See Methods and Figure S18 for details of the division of genus groups. The evolutionary rates of each genus were estimated using the global phylogeny with the constraint for the crown age of angiosperm to be 149-256 Ma.

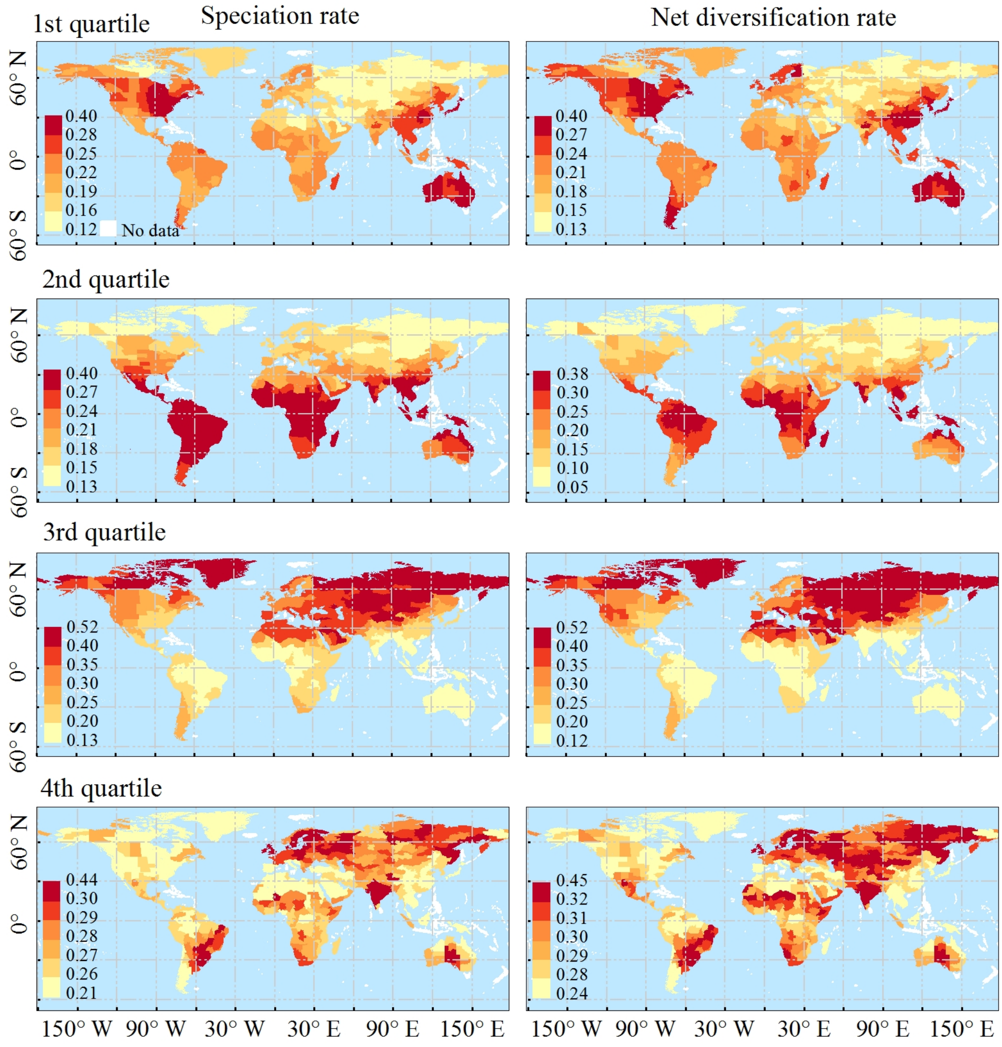

Figure S24 Patterns in the proportions of genera with different evolutionary rates. All genera were divided into four quartiles in an increasing order of their speciation rates (first column) and net diversification rates (second column) separately. See Methods and Figure S18 for details of the division of genus groups. The evolutionary rates of each genus were estimated using the molecular phylogeny with the constraint for the crown age of angiosperm to be 149-256 Ma.

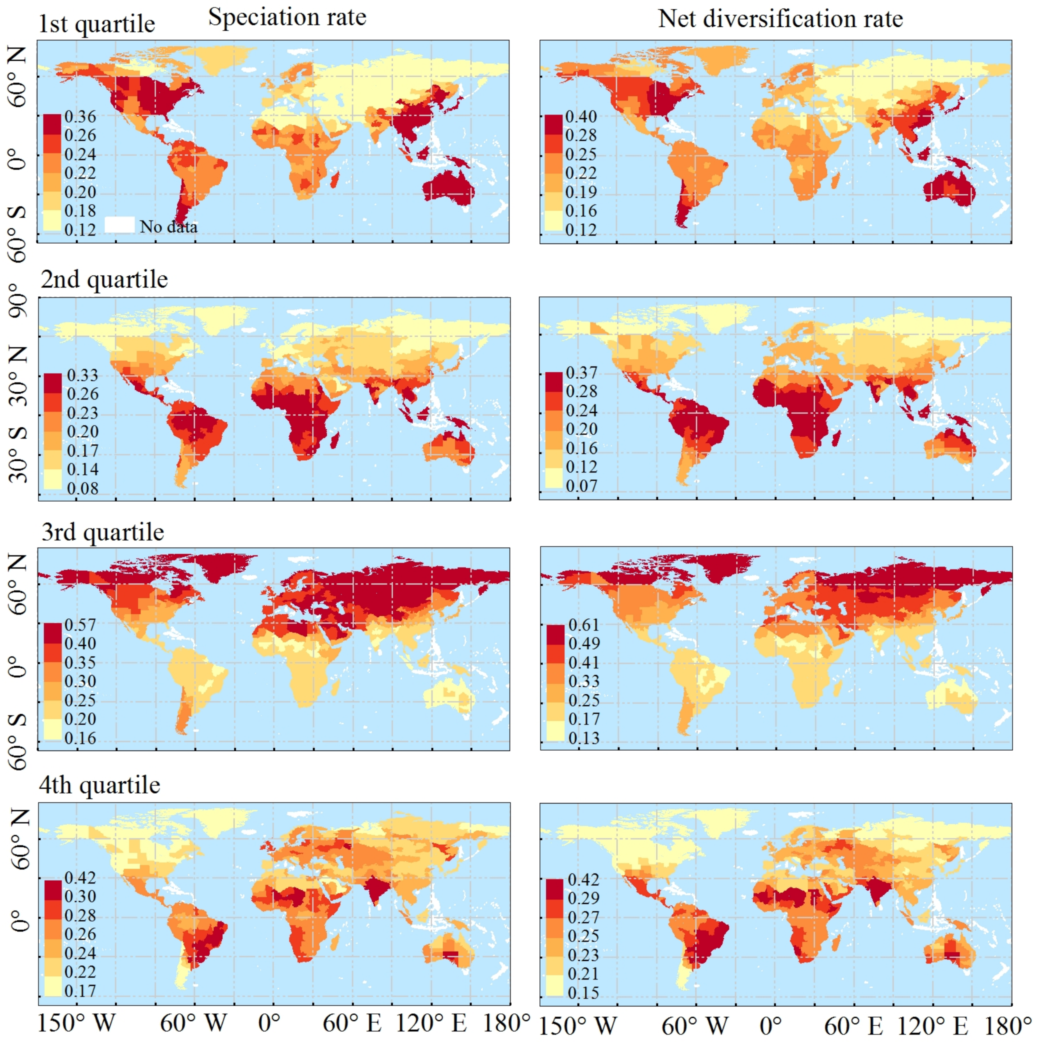

Figure S25. Comparison between speciation and net diversification rates of angiosperm genera with different life forms (herbaceous and Woody, $n_{\text{herbaceous}}$ = 5695 and n_{Woody} = 5722) and photosynthetic pathways (C4, CAM and C3, n_{C4} = 365, n_{CAM} = 337, and n_{others} = 13542) based on the global phylogeny dated using a constrain 140-150 Ma for the crown angiosperms. Wilcoxon rank-sum two-sided test was performed for genera with different life forms. Pairwise Mann–Whitney two-sided U-tests was performed for genera with different photosynthetic pathway with *P*-values were corrected using Benjamini-Hochberg method. The white dot shows the median in each violin plot, and the black box illustrate 25th and 75th percentiles, with whiskers extending to the largest value within 1.5-fold the interquartile range.

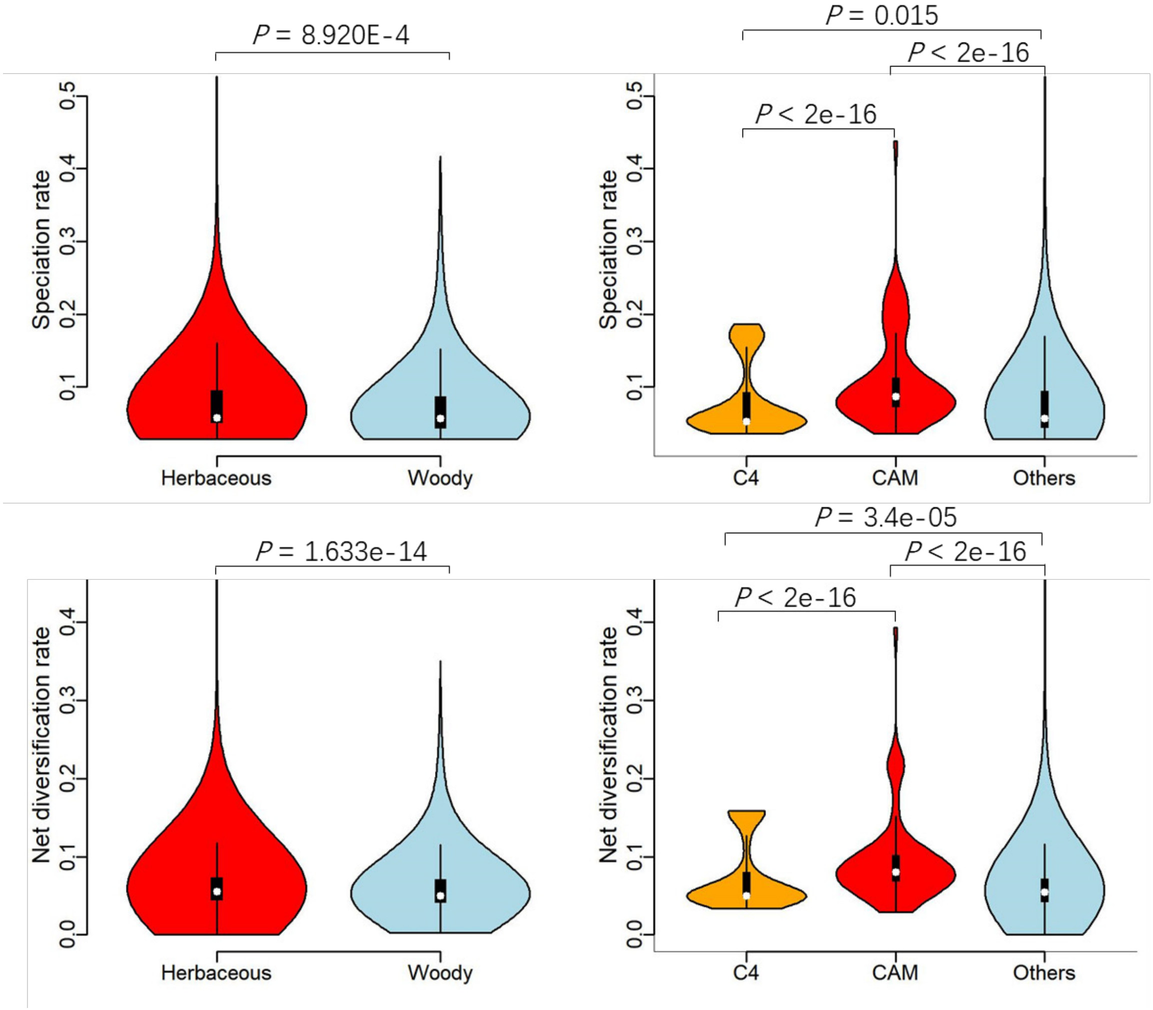

Figure S26. Relationships between log10 transformed generic richness (y-axis) and mean age and evolutionary rates across space estimated using model II geometric mean regression. The age and evolutionary rates were estimated using 1) the global (first row) and 2) molecular (second row) phylogenies with the constraint for the crown age of angiosperms being 140-150 Ma, 3) the global (third row) and 4) molecular (fourth row) phylogenies with the constraint for the crown age of angiosperms being 140-210 Ma, 5) the global (fifth row) and 6) molecular (sixth row) phylogenies with the constraint for the crown age of angiosperms being 149-256 Ma. r^2 was estimated by ordinary least square regression. Solid red lines represent the regression lines, individual data points are in blue.

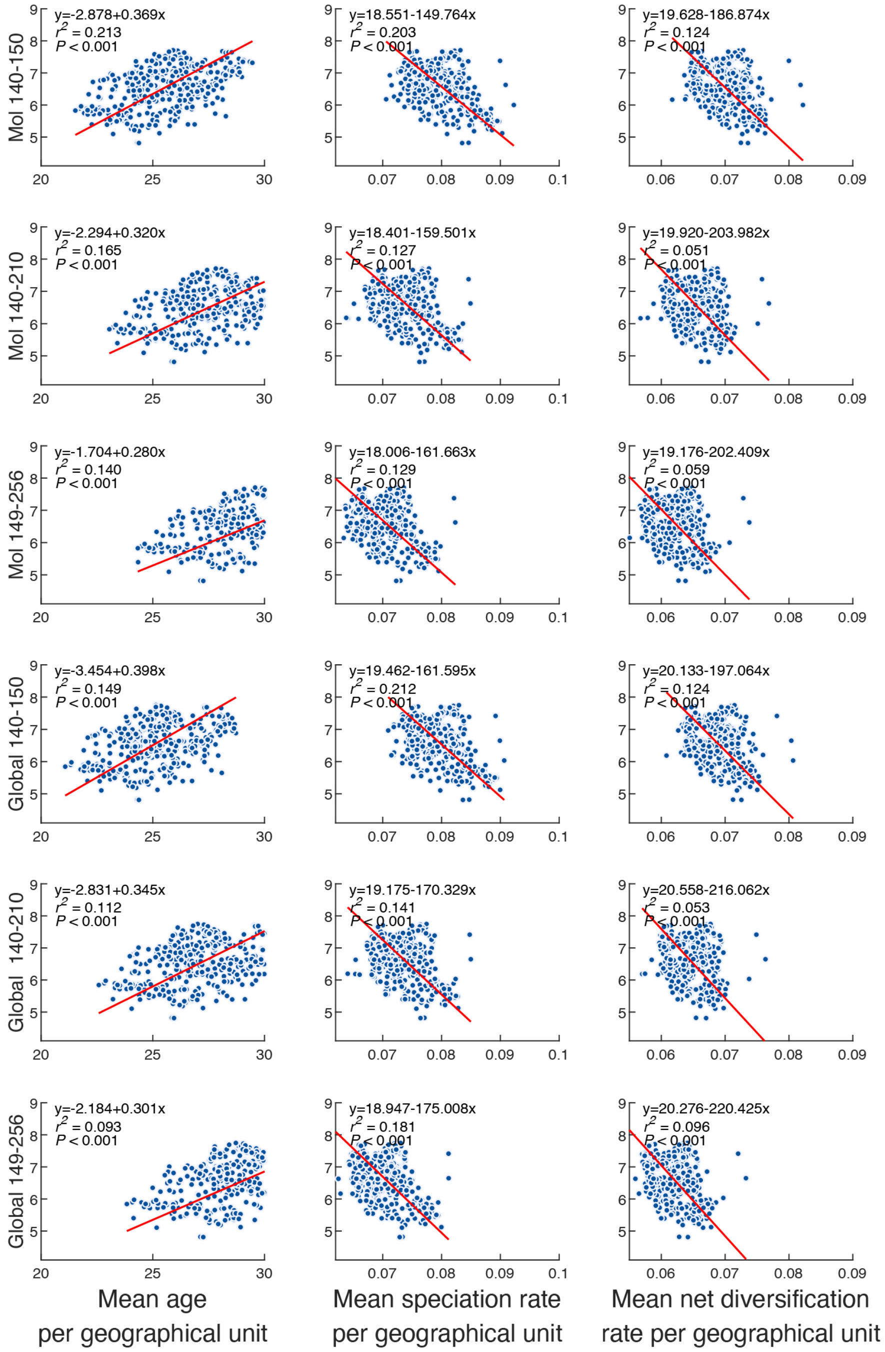

Figure S27. Frequency distribution (i.e. number of genera) for the number of congenic species used in molecular tree construction. In total, the sequences of ca. 54% of genera were from only one species, and two species for 24% of genera.

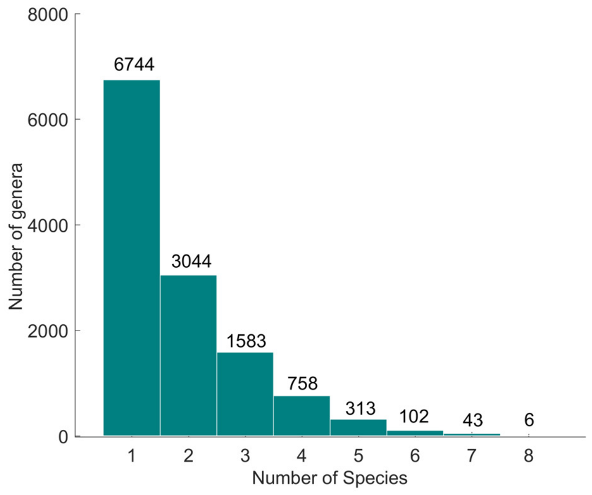

Figure S28. Comparison of tip speciation rates (the first column) and net diversification rates (the second column) estimated from the three different constraints for the crown age of angiosperms. The **x** axes represent the evolutionary rates estimated by the dating with the constraint being 140-150 Ma. The y axes represent the dating constraint being 140-210 Ma (the first and third row) and 149-256 Ma (the second and fourth row) respectively. The first two rows show the comparison between the three global trees, and the second two rows show the comparison between the three molecular trees. Solid red lines represent the linear regression lines, individual data points are in blue. r^2 presented in the figures were estimated by linear regressions.

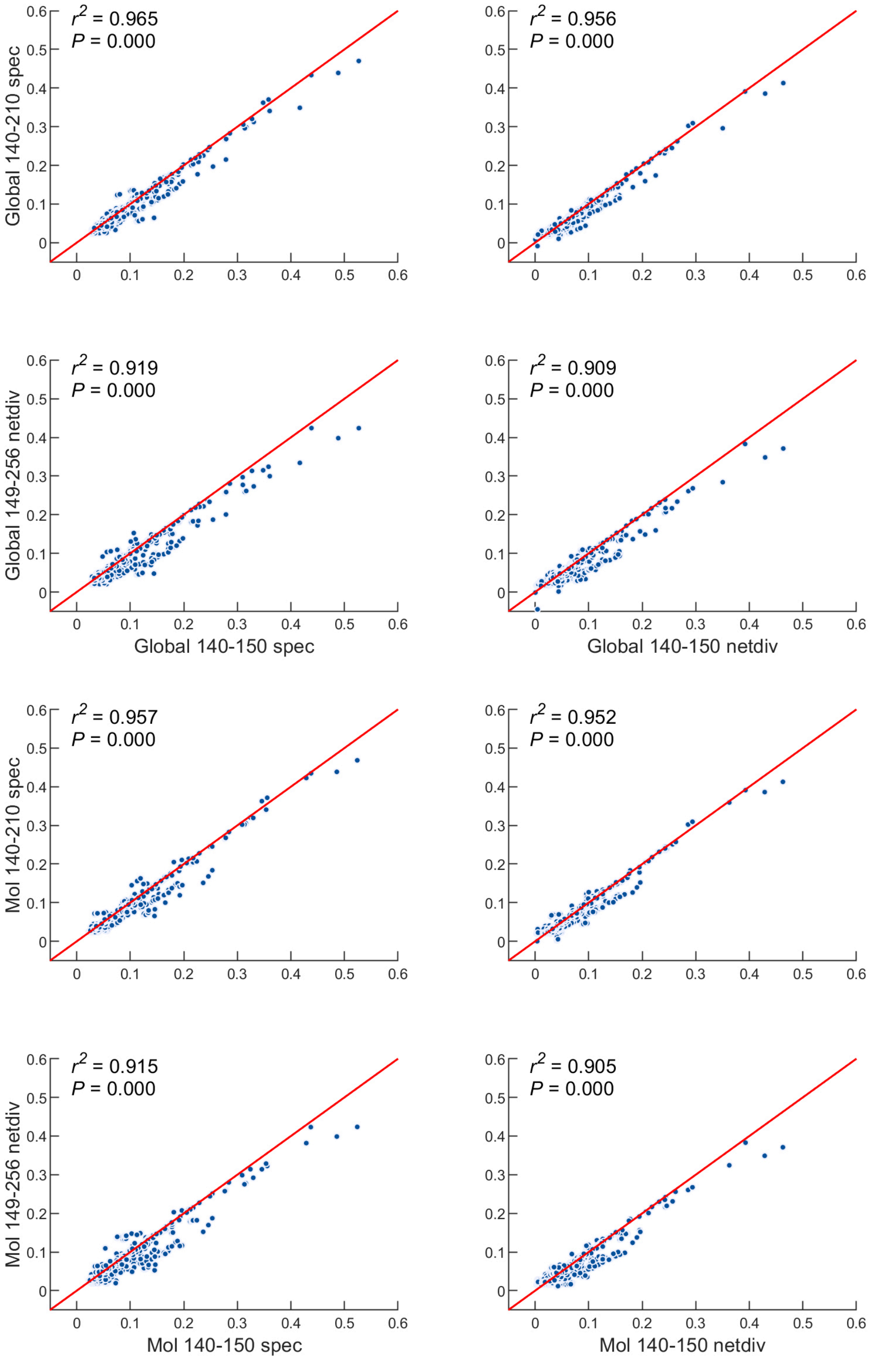

Supplement: Supplementary file 1 — Supplementary Information [file 41467_2023_43396_MOESM1_ESM.pdf]
