# Supplementary material for: Diversification of flowering plants in space and time
Source: Nat Commun. 2023 Nov 22;14:7609. doi: 10.1038/s41467-023-43396-8 (PMC10665465; doi:10.1038/s41467-023-43396-8)
Supplement: Supplementary file 2 — Description of Additional Supplementary Files [file 41467_2023_43396_MOESM2_ESM.pdf]

Description of Additional Supplementary Files

File Name: Supplementary Data 1
Description: Complete list of distribution data sources used to compile the distributional data presented in Supplementary Data 5.

File Name: Supplementary Data 2
Description: List of fossils used in the dating analyses and corresponding references.

File Name: Supplementary Data 3
Description: Families evolutionary rates estimated under the three alternatives for the age of crown Angiosperms.

File Name: Supplementary Data 4
Description: Genus list and GenBank accession numbers for all sequences used in the final analyses. The two additional sheets include the information on the sampling fractions used in the BAMM analyses based on the molecular and global phylogenies.

File Name: Supplementary Data 5
Description: Distributional data for all genera included in the analyses. The Database S5 sheet includes presence data for each genus and each geographical unit. Geographic units are referred by their ID and centroid coordinates. The Full Tree Spatial Pattern sheet include the data of species richness and mean diversification rates estimates under the three dating alternatives for each geographical unit. The Climate Chelsea sheet included that information on climatic variables for each geographical unit extracted from the Chelsea database (see Materials and methods in the main text for additional details). The corresponding shape file of the geographical units used is also provided.
