# Supplementary material for: Diversification of flowering plants in space and time
Source: Nat Commun. 2023 Nov 22;14:7609. doi: 10.1038/s41467-023-43396-8 (PMC10665465; doi:10.1038/s41467-023-43396-8)
Supplement: Supplementary file 3 — Supplementary Data 1 [file 41467_2023_43396_MOESM3_ESM.pdf]

Supplementary Data 2. List of distribution data sources

Literatures and Floras

1. Brouillet L, Desmet P, Coursol F, Meades SJ, Favreau M, Anions M, Bélisle P, Gendreau C, Shorthouse D, and contributors (2010+). Database of Vascular Plants of Canada (VASCAN). Online at http://data.canadensys.net/vascan. Data paper ID: doi: http://doi.org/10.3897/phytokeys.25.3100 [accessed time: 01-02-2016]
2. Conn, B.J. (2008+) Census of Vascular Plants of Papua New Guinea (http://www.pngplants.org/PNGCensus)(Access time: 03-05-2016).
3. National Museum of Natural History (U.S.). 2003. *Botanical Exploration in Myanmar*. Washington D.C: Smithsonian National Museum of Natural History. http://bibpurl.oclc.org/web/5856 http://persoon.si.edu/myanmar/. (Access time: 25-02-2016)
4. Instituto de Botánica Darwinion. Flora of Argentina. http://www.floraargentina.edu.ar/ (Access time: 01-09-2014)
5. University of Greifswald, Institute of Botany and Landscape Ecology, Institute of Geography and Geology, Computer Centre, 2010- (continuously updated). FloraGREIF - Virtual Flora of Mongolia (http://floragreif.uni-greifswald.de/floragreif/). Computer Centre of University of Greifswald, D-17487 Greifswald, Germany. (Access time: 01-02-2015)
6. Scott A. Mori, Melissa Tulig, Jean-Jacques de Granville, Sophie González, Véronique Guerin, Hervé Chevillotte & Jérôme Chave, FRENCH GUIANAN E-FLORA PROJECT. http://scum.nybg.org/science/projects/fg/. (Access time: 19-08-2014)
7. Miller, A.G., Cope, T.A. & Nyberg, J. (1996). Flora of the Arabian peninsula and Socotra. Edinburgh University Press in Association with Royal Botanic Garden.
8. Muthumperumal, C., & Parthasarathy, N. (2009). Angiosperms, climbing plants in tropical forests of southern Eastern Ghats, Tamil Nadu, India. Check List, 5(1), 092-111
9. Rai, T., & Rai, L. (1994). Trees of the Sikkim Himalaya. Indus Publishing.
10. The Rainforest Initiative. (2017) Trees of the rainforests of the Western Ghats. URL: https://www.rainforest-initiative.org/downloads/pdfs/wghats_trees.pdf
11. Gaikwad, K. N. & Mali, M. V. (2012). Tree flora of Nashik city (Maharashtra). International Journal of Life Science & Pharma Research. 2 (2), 94–101.
12. Pérez, R., & Condit, R. (2018). Tree atlas of Panama. URL: http://ctfs.arnarb.harvard.edu/webatlas/maintreeatlas.php.
13. Conn, B. J., & Damas, K. Q. (2006). Guide to trees of Papua New Guinea. URL: http://www.pngplants.org/PNGtrees/
14. Janzen, D. H. (Ed.). (2018). Costa Rican natural history. University of Chicago Press.
15. Fox, J. E. (1970). Preferred check-list of Sabah trees. Sabah forest record (Book 7). Borneo Literature Bureau, Sabah.
16. Lantican, C. B. (2015) Philippine Native Trees – What to Plant in Different Provinces. UPLB College of Forestry and Natural Resources, Laguna.

17. Hamann, A., Barbon, E. B., Curio, E., & Madulid, D. A. (1999). A botanical inventory of a submontane tropical rainforest on Negros Island, Philippines. Biodiversity & Conservation, 8(8), 1017-1031.
18. Culmsee, H., Pitopang, R., Mangopo, H., & Sabir, S. (2011). Tree diversity and phytogeographical patterns of tropical high mountain rain forests in Central Sulawesi, Indonesia. Biodiversity and Conservation, 20(5), 1103-1123.
19. Chaudhary, L., Kushwaha, A., Kumar, A., Bajpai, O., Pandey, J., & Srivastava, A. (2015). Tree species of the Himalayan Terai region of Uttar Pradesh, India: a checklist. Check List, 11, 1.
20. Soejarto, D. D., Regalado, J. C., Hiep, N. T., Loc, P. K., Bien, L. K., Bich, T. Q., ... & Kadushin, M. R. (2002). Atlas of Seed Plants of Cuc Phuong National Park. URL: (http://www.uic.edu/pharmacy/depts/ICBG/index.php).
21. Alves, R. J. V., & Kolbek, J. (2009). Summit vascular flora of Serra de São José, Minas Gerais, Brazil. Check list, 5(1), 035-073.
22. Manral, U., Raha, A., Solanki, R., Hussain, S. A., Babu, M. M., Mohan, D., & Talukdar, G. (2013). Plant species of Okhla Bird Sanctuary: a wetland of Upper Gangetic Plains, India [with erratum]. Check List, 9(2), 263-274.
23. Chong, K. Y., Tan, H. T., & Corlett, R. T. (2009). A checklist of the total vascular plant flora of Singapore: native, naturalised and cultivated species. URL: http://seadiv.org/node/30.
24. Culmsee, H., & Pitopang, R. (2009). Tree diversity in sub-montane and lower montane primary rain forests in Central Sulawesi. Blumea-Biodiversity, Evolution and Biogeography of Plants, 54(1-2), 119-123.
25. Yamada, I. (1975). Forest Ecological studies if the montane forest of Mt. Pangrango, West Java: I. Stratification and floristic composition of the mountain rain forest near Cibodas. Tonan Ajia Kenkyu (The Southeast Asian Studies), 13(3).
26. Beech, E., Rivers, M., Oldfield, S., & Smith, P. P. (2017). GlobalTreeSearch: The first complete global database of tree species and country distributions. Journal of Sustainable Forestry, 36(5), 454-489.
27. Saville, P. S., & Fox, J. E. D. (1967). Trees of Sierra Leone. Trees of Sierra Leone.
28. Kitamura, S. (1960). Flora of Afghanistan (Vol. 2). Kyoto Univ.
29. Väre, H., Daniels, F., Gillespie, L., & Poulin, M. (2013). Plants (Chapter 9). In Arctic Biodiversity Assessment: Status and Trends in Arctic Biodiversity (pp. 310-353). Conservation of Arctic Flora and Fauna (CAFF), Arctic Council.
30. Villaseñor, J. L. (2016). Checklist of the native vascular plants of Mexico. Revista Mexicana de Biodiversidad, 87(3), 559-902.
31. Cortés, S. (1897). Flora de Colombia: Volumen Primero. Comprende la flora terapéutica, la industrial, el catálogo de los nombres vulgares de las plantas y una introducción geológica (Vol. 1). Papeleria, Imprenta y Litografia de Samper Matiz.
32. Goodwin, Z. A., Lopez, G. N., Stuart, N., Bridgewater, S. G., Haston, E. M., Cameron, I. D., ... & Whitefoord, C. (2013). A checklist of the vascular plants of the lowland savannas of Belize, Central America. Phytotaxa, 101(1), 1-119.

33. Dauby, G., Zaiss, R., Blach-Overgaard, A., Catarino, L., Damen, T., Deblauwe, V., & Engledow, H. (2016). RAINBIO: a mega-database of tropical African vascular plants distributions. PhytoKeys, (74), 1.
34. Maitner, B. S., Boyle, B., Casler, N., Condit, R., Donoghue, J., Durán, S. M., & McGill, B. (2018). The bien r package: A tool to access the Botanical Information and Ecology Network (BIEN) database. Methods in Ecology and Evolution, 9(2), 373-379.
35. Liu Ningyan (chief editor). (1993). Annals of Taiwan Province Vol. 2 (land), Chapter Plant, Nantou City, Taiwan Province, Taiwan provincial literature Committee.
36. Editorial Committee of Sylva in Zhongtiao Mountain. (1995). Tree flora of Zhongtiao Mountain, Beijing, China Forestry Publishing House.
37. Jia Jingxian, Jia Dingxian, Ren Qingmian. (2006). Crop and its wild relatives in China-Fruit tree, Beijing, China Forestry Publishing House.
38. Pan Zhigang et al. (1994). Introduction and cultivation of the main foreign tree species in China, Beijing, Beijing Science and Technology Press.
39. Editorial Committee of CAS. (1959-2004). Flora Republicae Popularis Sinicae. Vol. 1-80, Beijing, Science Press. http://www.iplant.cn/frps
40. Liu Mengjun (editor in chief). (1998). Wild fruit trees in China., Beijing, Agriculture Press.
41. Editorial Committee of China Sylva. (1983). Tree flora of China, Beijing, China Forestry Publishing House.
42. Lanzhou Institute of CAS. (1992-1992). Desert flora of China, Beijing, Science Press.
43. Cheng Junqing, Yang Zongju, Liu Peng. (1992). Timber of China, Beijing, China Forestry Publishing House.
44. Qu Zezhou, Wang Yonghui (editor in chief). (1993). Fruit tree flora of China (Jujube part), Beijing, China Forestry Publishing House.
45. Guo Shanji (editor in chief). (1993). Fruit tree flora of China (Ginkgo part), Beijing, China Forestry Publishing House.
46. Zhang Jiayan, Zhang Zhao (editor in chief). (2003). Fruit tree flora of China (Apricot part), Beijing, China Forestry Publishing m,dde.
47. Wang Zuhua, Zhuang Enji (editor in chie\. (2001). Fruit Tree Flora of China (Peach part), Beijing, China Forestry Publishing House.
48. Zhao Huanzhun, Feng Baotian (editor in chief). (1996). Fruit Tree Flora of China (Hawthorn part), Beijing, China Forestry Publishing House.
49. Lu Qiunong, Jia Dingxian (editor in chief). (1999). Fruit tree flora of China (Apple part), Beijing, China Forestry Publishing House.
50. Zhu Meng. (1999). Fruit tree flora of China (Plum part), Beijing, China Forestry Publishing House.
51. Qiu Wuling, Zhang Huizhi (editor in chief). (1996). Fruit tree flora of China (Longan and loquat part), Beijing, China Forestry Publishing House.
52. Wu Shuxian (editor in chief). (1998). Fruit tree flora of China (Litchi part), Beijing, China Forestry Publishing House.

53. Zhang Jiayan, Zhou En (editor in chief). (1998). Fruit Tree Flora of China (plum part), Beijing, China Forestry Publishing House.
54. Xi Rongting, Zhang Yiping (Editor-in-chief). (1996). Fruit Tree Flora of China (Walnut part), Beijing, China Forestry Publishing House.
55. Deng Mingqin, Lei Jiajun. (2005). Fruit tree flora of China (Strawberry part), Beijing, China Forestry Publishing House.
56. Fu Liguo et al. (2000-2005). Chinese higher plants Vol. 3, 6, 8, 10, 11, 13, Qingdao, Qingdao press.
57. Bai Songlin and Wu Decheng. (1994). Flora of Greater Khingan Range, Harbin, Heilongjiang science and Technology Publishing House.
58. Editorial Committee of Zhejiang flora. (1992-1993). Flora of Zhejiang Vol. 1-7, Hangzhou, Zhejiang Science and Technology Press.
59. Department of Health of Zhejiang Province. (1960). Medicinal plant flora of Tianmushan mountain, Hangzhou, Zhejiang people's Publishing House.
60. Shanghai Museum of Natural History. (1989). Spore flora of the Yangtze River Delta and its adjacent area, Shanghai, Shanghai science and Technology Publishing House.
61. Committee for Zoning of Agriculture Area of Tonghua. (1985). Flora of wild economic plants in the southwest of Changbai Mountain, Changchun, Office of Tonghua District Agricultural Zoning Committee of Jilin Province.
62. Li Chunguang, Wang Yongming, Xuan Wu. (1990). Flora of wild economic plants in northeastern Changbai Mountain, Yanbian, Yanbian people's Publishing House.
63. Kunming Institute of Botany, CAS. (1997-2006). Flora of Yunnan Vol. 7-15, 18-21, Beijing, Science Press.
64. Southwest Forestry University. (1990). Atlas of trees in Yunnan, Kunming, Yunnan Science and Technology Press.
65. Editorial Committee of Local chronicles of Yunnan Province. (1993). Local chronicles of Yunnan Vol. 5 (Flora), Kunming, Yunnan People's Publishing House.
66. Sun Hang, Zhou Zhekun. (2002). Seed plant in the Brahmaputra Valley, Kunming, Yunnan Science and Technology Press.
67. TAnimal Husbandry Department of Xinjiang Uygur Autonomous Region. (1990). Main forage plants in Xinjiang, Urumchi, Volksverlag Xinjiang.
68. CAS, Xinjiang Institute of biological soil desert. (1977). Medicinal plants in Xinjiang, Urumchi, Volksverlag Xinjiang.
69. Zhou Yiliang. (1955). Woody plants in Xiao Hinggan Mountains, Beijing, China Forestry Publishing House.
70. Kunming Institute of Botany, CAS. (1984). Plant list in Xishuangbanna, Kunming, Yunnan National Publishing House.
71. Liu Jiayi (editor in chief). (2004). Flora of Tianjin, Tianjin, Tianjin science and Technology Publishing House.
72. Liu Tianwei, Zhang Yunfeng (chief editor). (1990). Flora of Taiyuan, Beijing, China Science and Technology Publishing House.
73. Yi Tongpei. (1997). Sichuan bamboo flora, Beijing, China Forestry Publishing House.

74. Editorial Committee of Sichuan flora. (1981). Flora of Sichuan, Chengdu, Sichuan people's Publishing House.
75. Agricultural Biotechnology Research Institute, Sichuan branch of CAS. (1962-1963). Sichuan wild economic plants, Chengdu, Sichuan people's Publishing House.
76. Yang Qinzhou. (1997). Sichuan tree distribution., Guizhou, Guizhou Science and Technology Publishing House.
77. Xing Fuwu, Yu Mingsi (editor in chief). (2000). Wild plants in Shenzhen, Beijing, China Forestry Publishing House.
78. Shanghai Academy of Sciences. (1999). Flora of Shanghai, Shanghai, Shanghai science and Technology Literature Publishing House.
79. Niu Chunshan (editor in chief). (1990). Tree flora of Shaanxi, Beijing, China Forestry Publishing House.
80. Xu Weiying, Le Tianyu. (1957). ShanGanNing basin flora, Beijing, China Forestry Publishing House.
81. Editorial Committee of Shanxi flora. (1992). Flora of Shanxi, Beijing, China Science and Technology Publishing House.
82. Shanxi Academy of forestry. (2001). Shanxi trees, Beijing, China Forestry Publishing House.
83. Committee for Zoning of Agriculture Area of Shanxi Province. (1991). The trees in Shanxi, Beijing, Science Press.
84. Li Huimin. (1990). Flora of Shanxi Province, Beijing, China Forestry Publishing House.
85. Committee for Zoning of Agriculture Area of Shanxi Province. (1990). Flora of Shanxi Province, Beijing, China Forestry Publishing House.
86. Wu Aiping, Kang Zhigang, Dong Fasheng, Gao Baoqin, Wang Xigui. (1999). Shanxi plant Heicha forest area of, Taiyuan, Shanxi Science and Technology Press.
87. Chen Hanbin (editor in chief). (1990). Flora of Shandong, Qingdao, Qingdao press.
88. Li Fazeng (editor in chief), Zhao Zuntian (editor). (2004). Shandong plant essence, Beijing, Science Press.
89. Huang Jinxiang, Li Xin, Qian Jinyuan. (1996). Saihanba flora, Beijing, China Science and Technology Publishing House.
90. Li Yaojie (editor in chief). (1987). Qinghai Woody Flora, Xining, Qinghai people's Publishing House.
91. Northwest Plateau Institute of biology, CAS. (1987). Qinghai Economic Plant, Xining, Qinghai people's Publishing House.
92. Nansha comprehensive scientific investigation team, CAS. (1996). Flora of Spratly Islands and its adjacent islands, Beijing, Ocean Press.
93. Nansha comprehensive scientific investigation team, CAS. (1996). Flora of Spratly Islands and its adjacent islands, Beijing, Ocean Press.
94. Li Shuxin (Editor in chief). (1988-1992). Flora of Liaoning, Shenyang, Liaoning Science and Technology Press.

95. Institute of forestry and soil, CAS, Liaoning Provincial Department of Commerce, Liaoning Provincial Department of Forestry, Shenyang College of pharmacy. (1960). Liaoning economic plant, Shenyang, Liaoning People's Publishing House.
96. Sun Xianwu. (1962). Lanzhou flora, Lanzhou, Gansu people's Publishing House.
97. Gao Weiheng. (1998). Kongtong mountain flora, Lanzhou, Gansu Culture Press.
98. Xin Zehua et al. (2002). Flora of Jiaozuo, Xi'an, Xi'an Map Publishing House.
99. Editor committee of flora of Jiangxi. (1993). Flora of Jiangxi, Beijing, China Science and Technology Publishing House.
100. Jiangsu Institute of Botany. (1977). Flora of Jiangsu, Nanjing, Jiangsu People's Publishing House.
101. Jiangsu Provincial Department of Commerce, Institute of plant science, CAS, Zhongshan Nanjing Botanical Garden. (1959). Jiangsu wild flora, Nanjing, Jiangsu People's Publishing House.
102. Committee for Zoning of Agriculture Area of Tonghua. (1961). Wild economic plants in Jilin Province, Changchun, Jilin People's Press.
103. Northwest Institute of Botany. (2000). Flora of the Loess Plateau, Beijing, Science Press.
104. Tan Peixiang. (1983). Southern China cuckoo, Guangzhou, Guangdong Science Press.
105. Chen Shouliang. (1962). Gramineae flora of East China, Nanjing, Jiangsu People's Publishing House.
106. Editorial Committee of Tree Flora in North China. (1984). Tree flora of North China, Beijing, China Forestry Publishing House.
107. Cui Youwen. (1953). Economic plants in North China, Beijing, Science Press.
108. Qi Chengjing, Yu Xunlin. (2002). Overview of seed plants in Hunan, Changsha, Hunan Science and Technology Press.
109. Editorial Committee of Hunan flora. (2000). Flora of Hunan Vol. 2, Changsha, Hunan Science and Technology Press.
110. Qi Chengjing, Lin, Qinzhong. (2000). Tree flora of Hunan, Changsha, Hunan Science and Technology Press.
111. Wuhan Institute of Botany, CAS. (2001). Flora of Hubei, Wuhan, Hubei Science and Technology Press.
112. Qinghai Tibet Plateau comprehensive scientific expedition team, CAS. (1994). Vascular plants in Hengduan mountains, Beijing, Science Press.
113. Zhou Yiliang (editor in chief). (1985). Flora of Heilongjiang, Harbin, Northeast Forestry University Press.
114. Ding Baozhang, Wang Suiyi and Gao Zengyi. (1981-1998). Flora of Henan, Zhengzhou, Henan people's Publishing House.
115. Compilation Committee of local chronicles in Henan. (1993). Local chronicles of Henan Vol. 7 (Flora), Zhengzhou, Henan people's Publishing House.
116. Yu Xueyou, Zhang Junpu, Lu Jionglin. (1998). Atlas of woody plants of Henan, Guangzhou, New Century Publishing House.
117. Editorial Committee of Henan Economic Plants. (1963). Flora of Henan economic plants, Zhengzhou, Henan people's Publishing House.

118.Editorial Committee of Hebei flora. (1986). Flora of Hebei, Shijiazhuang, Hebei Science & Technology Press.
119.Du Yibin (editor in chief). (2000). Flora of Hebei wild resources plants, Baoding, Hebei University Press.
120.Zhao Jiancheng, Wang Zhenjie, Li Lin (editor in chief). (2005). List of higher plants in Hebei, Beijing, Science Press.
121.Department of pharmacy, the first hospital of Shanghai. (1961). Medicinal plants in Hangzhou, Shanghai, Shanghai science and Technology Publishing House.
122.South China Institute of Botany, CAS. (1964-1977). Flora of Hainan, Beijing, Science Press.
123.Editorial Committee of Guizhou flora. (1982-2004). Flora of Guizhou, Guiyang, Guizhou people's Publishing House.
124.South China Institute of Botany, CAS. (1956). Flora of Guangzhou, Beijing, Science Press.
125.Guangxi Institute of plant science, Guangxi Academy of Sciences. (1991). Guangxi flora Vol. 1, Guilin, Guangxi science and Technology Publishing House.
126.South China Institute of Botany, CAS. (1987). Flora of Guangdong, Guangzhou, Guangdong science and Technology Publishing House.
127.An Dingguo. (2002). Flora of vascular plant plants in Xiaolongshan mountain , Lanzhou, Gansu National Publishing House.
128.Gansu Province Institute of apiculture. (1987). Gansu nectar flora, Lanzhou, Gansu science and Technology Publishing House.
129.Feng Zicheng, Xu Menglong. (1994). Atlas of tree in Gannan, Lanzhou, Gansu science and Technology Publishing House.
130.Institute of forestry and soil, CAS. (1959). Medicinal plants in Northeast China, Beijing, Science Press.
131.Liaoning Forestry Soil Research Institute. (1977). Moss flora of Northeast China, Beijing, Science Press.
132.Zhang Guangchu, Gao Qian. (1981). Liverworts flora of Northeast China, Beijing, Science Press.
133.Institute of forestry and soil, CAS. (1955). Atlas of woody plants of Northeast China, Beijing, Science Press.
134.Huang Dazhang (editor in chief), Bai Tongren (editor). (1964). Economic wood of Northeast China, Beijing, Science Press.
135.Han Quanzhong, Wang Zhengxing. (1993). Flora of Dalian, Dalian, Dalian University of Technology Press.
136.Zi Xingzhong, Zhang Dingcheng (editor in chief). (2006). Flora of the Dabie Mountains, Beijing, China Forestry Publishing House.
137.He Shiyuan et al. (1993). Flora of Beijing, Beijing Publishing House, Beijing.
138.Qu Zezhou (editor in chief). (1990). Beijing fruit trees, Beijing, Beijing Publishing House.
139.Mei Zhifen. (1999). Common trees in Beijing, Beijing, China Forestry Publishing House.

140.Editorial Committee of Florae Anhuiensis. (1986). Flora of Anhui Vol. 1-5, Hefei, Anhui Science and Technology Press.
141.Compilation group of woody plants in Anhui. (1983). Woody plants in Anhui, Hefei, Anhui Science and Technology Press.
142.Editorial Committee of Florae Anhui economic plants. (1990). Flora of Anhui economic plants, Hefei, Anhui Science and Technology Press.
143.Redactorum Florae Xinjiangensis Commissione. (1992-2004). Flora of Xinjiang Vol. 1-6, Urumqi, Xinjiang Science & Technology Publishing House.
144.Editor committee of Inner Mongolia flora. (1989-1998). Flora of Inner Mongolia (Second Edition), Hohhot, Inner Mongolia Peoples Publishing House.
145.Fang, J., Wang, Z., and Tang, Z. (2011). Atlas of Woody Plants in China Distribution and Climate (Berlin & Beijing Higher Education Press, Springer).
146.Li, Z.Y., and Wang, W.T. (2005). Plants of Gesneriaceae in China (Zhengzhou Henan Science and Technology Publishing House).
147.Tree Flora of Sabah and Sarawak Editorial Committee (1995-2007) Tree Flora of Sabah and Sarawak. Forest Research Institute Malaysia; Sabah Forestry Department, Malaysia; Sarawak Forestry Department, Malaysia, Malaysia.
148.Zhen Yu Li & Wen Tsai Wang (2005) Plants of Gesneriaceae in China. Henan Science and Technology Publishing House, Zhengzhou.
149.Qi Lu, Jihe Wang & Jianmin Chu (2012) Desert Plants in China. China Forestry Publishing House, Beijing.
150.John Norton, Sara Abdul Majid, Debbie Allan, Mohammed Alsafran, Benno Boer & Renee Richer (2009) An Illustrated Checklist of the Flora of Qatar. Browndown Publications, Gosport, UK.
151.Davis PH (1965-1985) Flora of Turkey and The East Aegean Islands. Edinburgh University Press, Edinburgh.
152.Editorial Committee of Flora Qinghaiica (1997-1999) Flora Qinghaiica. Qinghai people's Publishing House, Xining.
153.Kitamura S (1960) Flora of Afghanistan. Kyoto University, Kyoto.
154.
155.Editorial Committee of Flora of the U.S.S.R. (1963-1977) Flora of the U.S.S.R. (in English, translated from the Russian): Volume I to XXI; Volume XXIV. Keter Publishing House Jerusalem Ltd., Jerusalem, Israel.
156.Editorial Committee of Flora of the U.S.S.R. (1999-2004) Flora of the U.S.S.R. (in English, translated from the Russian) VOLUME XXII to XXIII; VOLUME XXV to XXIX. Smithsonian Institution Libraries Washington, D.C., U.S.A.
157.J. Undarmaa, T. Okuro, N. Manibazar & N. Yamanaka (2015) Rangeland Plants of Mongolia. Munkhiin Useg Co, Itd., Ulaanbaatar, Mongolia.
158.M. Urgamal, B. Oyuntsetseg, D. Nyambayar & Ch. Dulamsuren (2014) Conspectus of the vascular plants of Mongolia. Admon Printing Press, Ulaanbaatar, Mongolia.
159.Zhengyi Wu (1983-1987) Flora Xizangica. Science Press, Beijing.

160. Commissione Redactorum Florae Xinjiangensis (1992-2004) Flora of Xinjiang. Xinjiang Science & Technology Publishing House, Urumqi.
161. Yizhi Zhao (2006) Vascular Plants of Plateau Ordos. Inner Mongolia University Press, Hohhot.
162. Yizhi Zhao (2012) Classification and its Florristic Ecological Geographic Distribution of Vascular plant in Inner Mongolia. Inner Mongolia University Press, Hohhot.
163. Zongyuan Zhu, Cunzhu Liang & Zhigang Li (2011) Flora of Helan Mountain. Sunshine Press Co. LTD, Yinchuan.
164. Plant diversity in Paraguay: https://www.nhm.ac.uk/research-curation/scientific-resources/collections/botanical-collections/plants-paraguay/index.html. (Access time: 03-08-2016)
165. Hyde, M.A., Wursten, B.T., Ballings, P. & Coates Palgrave, M. (2020). Flora of Mozambique. URL: https://www.mozambiqueflora.com/index.php, retrieved 05-2016 Flora of Zimbabwe http://www.zimbabweflora.co.zw/index.php
166. Hyde, M.A., Wursten, B.T., Ballings, P. & Coates Palgrave, M. (2020). Flora of Botswana. URL: https://www.botswanaflora.com/index.php, retrieved 04-2016.
167. Hyde, M.A., Wursten, B.T., Ballings, P. & Coates Palgrave, M. (2020). Flora of Malawi. URL: https://www.malawiflora.com/index.php, retrieved 04-2016.
168. Hyde, M.A., Wursten, B.T., Ballings, P. & Coates Palgrave, M. (2020). Flora of Zimbabwe. URL: https://www.zimbabweflora.co.zw/index.php, retrieved 04-2016.
169. Hyde, M.A., Wursten, B.T., Ballings, P. & Coates Palgrave, M. (2020). Flora of Caprivi. https://www.capriviflora.com/index.php, retrieved 04-2016.
170. Bingham, M.G., Willemen, A., Wursten, B.T., Ballings, P. and Hyde, M.A. (2020). Flora of Zambia. URL: https://www.zambiaflora.com/index.php, 04-2016.
171. Flora of Equatorial Guinea. http://www.floradeguinea.com

172. Instituto de Investigación de Recursos Biológicos Alexander von Humboldt. 2015. Plantas vasculares que se han reportado en los bosques secos de Colombia. URL: http://ipt.sibcolombia.net/iavh/resource.do?r=bosquessecos_colombia_2013. Retrieved 09-2015.
173. APG III. 2009. An update of the Angiosperm Phylogeny Group classification for the orders and families of flowering plants. Botanical Journal of Linnean Society.161: 105-121.
174. Bernal, R., G. Galeano, A. Rodríguez, H. Sarmiento y M. Gutiérrez. 2013. Nombres Comunes de las Plantas
de Colombia.http://www.biovirtual.unal.edu.co/nombrescomunes/
175. Bernal, R., R.S. Gradstein & M. Celis. Manuscrito. Catálogo de las Plantas de Colombia. A ser publicado en Regnum Vegetabile.
176. Calderón, E.1998. Listas Rojas Preliminares de Plantas Vasculares de Colombia, incluyendo orquídeas. Instituto de Investigación de Recursos Biológicos Alexander von Humboldt. [on-line].
URL: http://www.humboldt.org.co/conservacion/plantas_amenazadas.htm

177.Calderón, E., G. Galeano & N. García (eds.). 2002. Libro Rojo de Plantas Fanerógamas de Colombia. Volumen 1: Chrysobalanaceae, Dichapetalaceae y Lecythidaceae. La serie Libros rojos de especies amenazadas de Colombia. Bogotá, Colombia. Instituto Alexander von Humboldt, Instituto de Ciencias Naturales-Universidad Nacional de Colombia, Ministerio del Medio Ambiente.
178.Calderón, E., G. Galeano &N. García (eds.). 2005. Libro Rojo de Plantas de Colombia. Volumen 2: Palmas, Frailejones y Zamias. Serie Libros Rojos de Especies Amenazadas de Colombia. Bogotá, Colombia. Instituto Alexander von Humboldt - Instituto de Ciencias Naturales de la Universidad Nacional de Colombia - Ministerio del Medio Ambiente, Vivienda y Desarrollo Territorial, 454 pp.
179.Calderón-Sáenz E. (ed.). 2007. Libro Rojo de Plantas de Colombia. Volumen 6: Orquídeas, Primera Parte. Serie Libros Rojos de Especies Amenazadas de Colombia. Bogotá, Colombia. Instituto Alexander von Humboldt - Ministerio de Ambiente, Vivienda y Desarrollo Territorial. 828 p.
180.Cárdenas, L. &N.R. Salinas(eds.). 2007. Libro Rojo de Plantas de Colombia. Volumen 4: Especies maderables amenazadas: Primera parte. Serie Libros Rojos de Especies Amenazadas de Colombia. Bogotá, Colombia. Instituto Amazónico de Investigaciones Científicas SINCHI - Ministerio del Medio Ambiente, Vivienda y Desarrollo Territorial. 323 pp.
181.García, N. (ed.). 2007. Libro Rojo de Plantas de Colombia. Volumen 5: Las magnoliáceas, las miristicáceas y las podocarpáceas. Serie Libros Rojos de Especies Amenazadas de Colombia. Bogotá, Colombia. Instituto Alexander von Humboldt - CORANTIOQUIA - Jardín Botánico Joaquín Antonio Uribe de Medellín - Instituto de Ciencias Naturales de la Universidad Nacional de Colombia – Ministerio de Ambiente, Vivienda y Desarrollo Territorial. 236 p.
182.García, N. & G. Galeano (eds.). 2006. Libro Rojo de Plantas de Colombia. Volumen 3: Las bromelias, las labiadas y las pasifloras. Serie Libros Rojos de Especies Amenazadas de Colombia. Bogotá, Colombia. Instituto Alexander von Humboldt - Instituto de Ciencias Naturales de la Universidad Nacional de Colombia - Ministerio del Medio Ambiente, Vivienda y Desarrollo Territorial.
183.Hoyos-Gómez, S. E., A. Idárraga, J. Betancur & A. Upegui. 2013. Costa y bosque: plantas del Chocó Darién-Caribe. Guía ilustrada. Corporación Fragmentos. Medellín, Colombia. 187 p.
184.Idárraga P., A. & R. Callejas P. 2011. Listado de las plantas vasculares del Departamento de Antioquia. Pp 127-923. En: A. Idárraga, R. del C. Ortíz, R. Callejas & M. Merello (eds). Flora de Antioquia: catálogo de las plantas vasculares. Vol. II. Listado de las plantas vasculares del departamento de Antioquia. Programa Expedición Antioquia-2013. Series Biodiversidad y Recursos Naturales. Universidad de Antioquia, Missouri Botanical Garden & Oficina de planeación departamental de la Gobernación de Antioquia, Editorial D’Vinni, Bogotá, Colombia.

185.Jardín Botánico de la Universidad de Caldas y Corpocaldas.2011. Informe del Taller Regional para la Conservación de Plantas amenazadas Eje Cafetero. Manizales, Mayo 26 y 27 de 2011.
186.Instituto de Ciencias Naturales, Facultad de Ciencias, Universidad Nacional de Colombia. 2004 (y continuamente actualizado). Colecciones en Línea. Publicado en Internet http://www.biovirtual.unal.edu.co [accesado el 14 Octubre 2013]. Instituto de Investigación de Recursos Biológicos Alexander von Humboldt.1998. El Bosque seco Tropical (Bs-T) en Colombia. Programa de Inventario de la Biodiversidad Grupo de Exploraciones y Monitoreo Ambiental GEMA.
187.Instituto de Investigación de Recursos Biológicos Alexander von Humboldt, Red Nacional de Jardines Botánicos de Colombia & Universidad de los Llanos. 2012. Memorias Encuentro Regional para la Priorización de Especies Vegetales en la Orinoquia. Noviembre 12 y 13 de 2012, Villavicencio. Convenio de Cooperación no. 12-12-067-173 CE
188.Instituto de Investigación de Recursos Biológicos Alexander von Humboldt, Red Nacional de Jardines Botánicos de Colombia & Jardín Botánico de la Quinta de San Pedro Alejandrino. 2013. Memorias Encuentro Regional para la Priorización de Especies Vegetales en el Caribe. Agosto 1y 2 de 2013, Santa Marta. Convenio de Cooperación no. 13-13-0086-112CE.
189.M. Rodríguez, G., K. Banda-R, S.P. Reyes & A.C. Estupiñán. 2012. Lista comentada de las plantas vasculares de bosques secos prioritarios para la conservación en los departamentos de Atlántico y Bolívar (Caribe Colombiano). Biota Colombiana, Volumen 13, Número 2, Especial Bosque Seco en Colombia. ISSN 0124-5376
190.Mendoza-C., H. 1999. Estructura y riqueza florística del bosque seco tropical en la región caribe y el valle del río Magdalena, Colombia. Caldasia 21 (1): 70-94. Bogotá.
191.The International Plant Names Index. 2012. Published on the Internet; http://www.ipni.org (accessed 14 October 2013).
192.The Plant List. 2010. Version 1. Published on the Internet; http://www.theplantlist.org/ (accessed 14 October 2013).
193.TROPICOS. 2013. Tropicos.org. Missouri Botanical Garden. Published on the Internet; http://www.tropicos.org(accessed 14 October 2013).
194.Tryon, R.M. & A.F. Tryon. 1982. Ferns and Allied Plants, with Special Reference to Tropical America. Berlín: Springer-Verlag.
195.Vargas, W. Los 2012. Los bosques secos del Valle de Cauca, Colombia: una aproximación a su flora actual. Biota Colombiana, Volumen 13, Número 2, Especial Bosque Seco en Colombia. ISSN 0124-5376
196.Catálogo de Plantas e Fungos do Brasil - versão 2010 (Volumes 1 and 2). URL: http://dspace.jbrj.gov.br/jspui/handle/doc/35
197.Flora Zambesiaca http://apps.kew.org/efloras/search.do

Tropicos Projects and efloras

The Tropicos database links over 1.33M scientific names with over 4.87M specimens and over 685K digital images. The data includes over 150K references from over 52.6K publications offered as a free service to the world's scientific community.
Project List: http://legacy.tropicos.org/ProjectList.aspx (including online floras from www.efloras.org), retrieved 06-2016.

198. Annotated Checklist of the Flowering Plants of Nepal, URL: http://www.efloras.org/flora_page.aspx?flora_id=110, retrieved 04-2016.
199. Flora of Chile: http://www.efloras.org/flora_page.aspx?flora_id=60
200. Flora of China: http://www.efloras.org/flora_page.aspx?flora_id=2
201. Flora of North America: http://www.efloras.org/flora_page.aspx?flora_id=1
202. Base de Datos de Minera Panamá: http://legacy.tropicos.org/Project/MPSA.
203. Base de Datos de Bolivia Catalogue: http://legacy.tropicos.org/Project/BC
204. Catálogo de las Plantas Vasculares de la Selva Central, PERÚ: http://legacy.tropicos.org/Project/SCP
205. Catalogue of New World Grasses: http://legacy.tropicos.org/Project/CNWG
206. Catalogue of the Plants of Madagascar: http://legacy.tropicos.org/Project/Madagascar
207. Catalogue of the Vascular Plants of Ecuador: http://legacy.tropicos.org/Project/CE
208. Catalogue of the Vascular Plants of the Department of Antioquia (Colombia): http://legacy.tropicos.org/Project/Catalogo-de-Antioquia
209. Flora of Azerbaijan: http://legacy.tropicos.org/Project/Azerbaijan
210. Flor of Missouri: http://www.efloras.org/flora_page.aspx?flora_id=11
211. Flora de Nicaragua: http://legacy.tropicos.org/Project/FN
212. Flora of Pakistan: http://legacy.tropicos.org/Project/Pakistan
213. Flora Palaestina: http://legacy.tropicos.org/Project/Palaestina
214. Flora of Panama (WFO): http://legacy.tropicos.org/Project/FOPWFO
215. Manual de Plantas de Costa Rica: http://legacy.tropicos.org/Project/Costa%20Rica
216. Madidi Checklist: http://legacy.tropicos.org/Project/MDICHK
217. Panama Checklist: http://legacy.tropicos.org/Project/PAC
218. Paraguay Checklist: http://legacy.tropicos.org/Project/Paraguay
219. Paramos de Antioquia: http://legacy.tropicos.org/ProjectList.aspx
220. Peru Checklist: http://legacy.tropicos.org/Project/PEC
221. Trees and shrubs of the Andes of Ecuador (eflora): http://www.efloras.org/flora_page.aspx?flora_id=201

African plant database(AFR)
The database currently comprises 198, 527 names of Aafrican plants with their nomenclatural statuts. Data capture, edition and broadcast are the product of a collaboration between the South African National Biodiversity Institute, the Conservatoire et Jardin botaniques de la Ville de Genève, Tela Botanica and the Missouri Botanical Garden . For more information: www.ville-ge.ch/musinfo/bd/cjb/africa/recherche.php

222. **Tropical Africa:** J.-P. LEBRUN & A. L. STORK (1991-2015). Enumération des plantes à fleurs d'Afrique tropicale et Tropical African Flowering Plants: Ecology and Distribution, vol. 1-7. Conservatoire et Jardin botaniques de la Ville de Genève.

223.**Southern Africa:** G. GERMISHUIZEN & N.L. MEYER, eds, (2003). Plants of Southern Africa: an annotated checklist Pretoria.
224.**North Africa:** DOBIGNARD, A. & C. CHATELAIN (2010-2013) Synonymic and bibliographic index of North Africa plants. vol. 1-5.
225.**Madagascar:** Catalogue of the Vascular Plants of Madagascar. Missouri Botanical Garden, St. Louis.

Other sources of African plant distributions

226.African Plant Database (version 3.4.0). Conservatoire et Jardin botaniques de la Ville de Genève and South African National Biodiversity Institute, Pretoria, "Retrieved 05-2016", from <http://www.ville-ge.ch/musinfo/bd/cjb/africa/>.
227.South African National Biodiversity Institute (SANBI): http://www.sanbi.org
228.South African Botanical Diversity Network (SABONET): http://www.sabonet.org.za
229.Tela Botanica http://www.tela-botanica.org
230.Catalogue des plantes de Madagascar http://www.efloras.org/madagascar
231.Checklist and distribution of the liverworts and hornworts of sub-Saharan Africa, including the East African Islands: http://www.oshea.demon.co.uk/tbr/tbrr3.htm
232.Checklist des mousses de l'Afrique sub-Saharienne (v.4): http://www.oshea.demon.co.uk/tbr/tbrr4.htm
233.Checklist des lichens d'Afrique: http://www.biologie.uni-hamburg.de/checklists/africa_l0.htm
234.MedCheckList http://ww2.bgbm.org/mcl/query.asp
235.Genera: Index Nominum Genericorum http://www.botany.si.edu/ing/
236.Global Compositae Checklist http://www.compositae.org/checklist
237.Digital library of Real Jardin Botanico, Madrid http://bibdigital.rjb.csic.es/ing/
238.Orchidaceae en Afrique centrale, http://www.orchid-africa.net/
239.Plants of the World online. http://plantsoftheworldonline.org/
240.Herbier de Gérard de Belair (Est Algérien) http://gdebelair.com
241.Biodiversité végétale du sud-ouest marocain http://www.teline.fr/fre
242.Flore du Sahara http://www.sahara-nature.com/flore_sahara.php
243.Plantes Afrique du Nord dessins https://commons.wikimedia.org/wiki/Category:Plantes_Afrique_du_Nord_dessins
244.African Plants - a photoguide, http://www.westafricanplants.senckenberg.de/root/index.php
245.Fleurs d'Afrique, Cirad France http://fleurs.cirad.fr/fleurs_d_afrique_tropicale
246.ALUKA, African Plants Initiative http://www.aluka.org

World Checklist of Selected Plant Families (WCSP)

WCSP is an international collaborative programme that provides the latest peer reviewed and published opinions on the accepted scientific names and synonyms of selected plant families. It contains all the scientific names of a particular plant, or the areas of the world in which it grows (distribution). The checklist includes 173 Seed Plant families (View list of

included families). Different families are in different stages of review as indicated in the family list. There are currently more than 155 contributors from 22 countries. For more informations: http://apps.kew.org/wcsp/home.do

247. Govaerts, R., Farjon, A. (2014). World Checklist of Pinaceae. Facilitated by the Royal Botanic Gardens, Kew. Published on the Internet; http //apps.kew.org/wcsp/ Retrieved 2014_8_1.
248. Govaerts, R., Farjon, A. (2014). World Checklist of Podocarpaceae. Facilitated by the Royal Botanic Gardens, Kew. Published on the Internet; http //apps.kew.org/wcsp/ Retrieved 2014_8_1.
249. Govaerts, R., Farjon, A. (2014). World Checklist of Sciadopityaceae. Facilitated by the Royal Botanic Gardens, Kew. Published on the Internet; http //apps.kew.org/wcsp/ Retrieved 2014_8_1.
250. Govaerts, R., Farjon, A. (2014). World Checklist of Taxaceae. Facilitated by the Royal Botanic Gardens, Kew. Published on the Internet; http //apps.kew.org/wcsp/ Retrieved 2014_8_1.
251. Govaerts, R., Farjon, A. (2014). World Checklist of Araucariaceae. Facilitated by the Royal Botanic Gardens, Kew. Published on the Internet; http //apps.kew.org/wcsp/ Retrieved 2014_8_1.
252. Govaerts, R., Farjon, A. (2014). World Checklist of Cupressaceae. Facilitated by the Royal Botanic Gardens, Kew. Published on the Internet; http //apps.kew.org/wcsp/ Retrieved 2014_8_1.
253. Govaerts, R., Leeuwenberg, A. (2014). World Checklist of Apocynaceae. Facilitated by the Royal Botanic Gardens, Kew. Published on the Internet; http //apps.kew.org/wcsp/ Retrieved 2014_8_1.
254. Govaerts, R., Paton, A., Y.Harvey, Y., Navarro, T. & M.del Rosario García Peña (2014). World Checklist of Lamiaceae. Facilitated by the Royal Botanic Gardens, Kew. Published on the Internet; http //apps.kew.org/wcsp/ Retrieved 2014_8_1.
255. Govaerts, R., Radcliffe-Smith, A. (2014). World Checklist of Pandaceae. Facilitated by the Royal Botanic Gardens, Kew. Published on the Internet; http //apps.kew.org/wcsp/ Retrieved 2014_8_1.
256. Govaerts, R., Radcliffe-Smith, A. (2014). World Checklist of Petrosaviaceae. Facilitated by the Royal Botanic Gardens, Kew. Published on the Internet; http //apps.kew.org/wcsp/ Retrieved 2014_8_1.
257. Govaerts, R., Briggs, B.G. & Linder, P. (2014). World Checklist of Restionaceae. Facilitated by the Royal Botanic Gardens, Kew. Published on the Internet; http //apps.kew.org/wcsp/ Retrieved 2014_8_1.
258. Govaerts, R., Zonneveld, B.J.M. & Zona, S.A. (2014). World Checklist of Asparagaceae. Facilitated by the Royal Botanic Gardens, Kew. Published on the Internet; http //apps.kew.org/wcsp/ Retrieved 2014_8_1.
259. Govaerts, R., Hong-Wa, C. & Phillipson, P. (2014). World Checklist of Sarcolaenaceae. Facilitated by the Royal Botanic Gardens, Kew. Published on the Internet; http //apps.kew.org/wcsp/ Retrieved 2014_8_1.

260.Govaerts, R., Dransfield, J., Zona, S.F, Hodel, D.R. & Henderson, A. (2014). World Checklist of Arecaceae. Facilitated by the Royal Botanic Gardens, Kew. Published on the Internet; http //apps.kew.org/wcsp/ Retrieved 2014_8_1.
261.Govaerts, R., Fernández Casas, F.J., Barker, C., Carter, S., Davies, S., Esser, H.-J., Gilbert, M., Hoffmann, P., Radcliffe-Smith, A., Steinmann, V., van Welzen, P. & Whitmoore, T. (2014). World Checklist of Euphorbiaceae. Facilitated by the Royal Botanic Gardens, Kew. Published on the Internet; http //apps.kew.org/wcsp/ Retrieved 2014_8_1.
262.Govaerts, R., Prance, G.T. & Sothers, C.A. (2014). World Checklist of Chrysobalanaceae. Facilitated by the Royal Botanic Gardens, Kew. Published on the Internet; http //apps.kew.org/wcsp/ Retrieved 2014_8_1.
263.Govaerts, R., Luther, H.E. & Grant, J. (2014). World Checklist of Bromeliaceae. Facilitated by the Royal Botanic Gardens, Kew. Published on the Internet; http //apps.kew.org/wcsp/ Retrieved 2014_8_1.
264.Govaerts, R., Esser, H.-J., Frodin, D.G., Lowry, P.P., Wen, J. (2014). World Checklist of Araliaceae. Facilitated by the Royal Botanic Gardens, Kew. Published on the Internet; http //apps.kew.org/wcsp/ Retrieved 2014_8_1.
265.Govaerts, R., Kennedy, H. (2014). World Checklist of Marantaceae. Facilitated by the Royal Botanic Gardens, Kew. Published on the Internet; http //apps.kew.org/wcsp/ Retrieved 2014_8_1.
266.Govaerts, R., Maas-van de Kamer, H. & Maas, P.J.M. (2014). World Checklist of Haemodoraceae. Facilitated by the Royal Botanic Gardens, Kew. Published on the Internet; http //apps.kew.org/wcsp/ Retrieved 2014_8_1.
267.Govaerts, R., Maas-van de Kamer, H. & Maas-van de Kamer, P. (2014). World Checklist of Costaceae. Facilitated by the Royal Botanic Gardens, Kew. Published on the Internet; http //apps.kew.org/wcsp/ Retrieved 2014_8_1.
268.Govaerts, R., Maas-van de Kamer, H. & Maas-van de Kamer, P. (2014). World Checklist of Triuridaceae. Facilitated by the Royal Botanic Gardens, Kew. Published on the Internet; http //apps.kew.org/wcsp/ Retrieved 2014_8_1.
269.Govaerts, R., Maas-van de Kamer, H. & Maas-van de Kamer, P. (2014). World Checklist of Cannaceae. Facilitated by the Royal Botanic Gardens, Kew. Published on the Internet; http //apps.kew.org/wcsp/ Retrieved 2014_8_1.
270.Govaerts, R., Häkkinen (2014). World Checklist of Musaceae. Facilitated by the Royal Botanic Gardens, Kew. Published on the Internet; http //apps.kew.org/wcsp/ Retrieved 2014_8_1.
271.Govaerts, R., Hofreiter, A. (2014). World Checklist of Alstroemeriaceae. Facilitated by the Royal Botanic Gardens, Kew. Published on the Internet; http //apps.kew.org/wcsp/ Retrieved 2014_8_1.
272.Govaerts, R., Bogner, J., Boos, J., Boyce, P., Cosgriff, B., Zhu, G., Thompson, S., Croat, T., Gonçalves, E., Grayum, M., Hay, A., Hetterscheid, W., Ittenbach, S., Landolt, E., Mayo, S., Murata, J., Nguyen, V.D., Sakuragui, C.M. & Singh, Y. (2014). World

Checklist of Araceae. Facilitated by the Royal Botanic Gardens, Kew. Published on the Internet; http //apps.kew.org/wcsp/ Retrieved 2014_8_1.
273. Govaerts, R., Jaszczerski, J.C. (2014). World Checklist of Mayacaceae. Facilitated by the Royal Botanic Gardens, Kew. Published on the Internet; http //apps.kew.org/wcsp/ Retrieved 2014_8_1.
274. Govaerts, R., Kress, J. (2014). World Checklist of Heliconiaceae. Facilitated by the Royal Botanic Gardens, Kew. Published on the Internet; http //apps.kew.org/wcsp/ Retrieved 2014_8_1.
275. Govaerts, R., Lock, J.M. (2014). World Checklist of Xyridaceae. Facilitated by the Royal Botanic Gardens, Kew. Published on the Internet; http //apps.kew.org/wcsp/ Retrieved 2014_8_1.
276. Govaerts, R., Barringer, K.A. (2014). World Checklist of Schlegeliaceae. Facilitated by the Royal Botanic Gardens, Kew. Published on the Internet; http //apps.kew.org/wcsp/ Retrieved 2014_8_1.
277. Govaerts, R., Hill, K.D. (2014). World Checklist of Cycadaceae. Facilitated by the Royal Botanic Gardens, Kew. Published on the Internet; http //apps.kew.org/wcsp/ Retrieved 2014_8_1.
278. Govaerts, R., Hill, K.D. (2014). World Checklist of Zamiaceae. Facilitated by the Royal Botanic Gardens, Kew. Published on the Internet; http //apps.kew.org/wcsp/ Retrieved 2014_8_1.
279. Govaerts, R., Persson, K. (2014). World Checklist of Colchicaceae. Facilitated by the Royal Botanic Gardens, Kew. Published on the Internet; http //apps.kew.org/wcsp/ Retrieved 2014_8_1.
280. Govaerts, R., Kington, S., Friesen, N., Fritsch, R., Snijman, D.A., Marcucci, R., Silverstone-Sopkin, P.A. & Brullo, S. (2014). World Checklist of Amaryllidaceae. Facilitated by the Royal Botanic Gardens, Kew. Published on the Internet; http //apps.kew.org/wcsp/ Retrieved 2014_8_1.
281. Govaerts, R., Lohmann, L.G. (2014). World Checklist of Bignoniaceae. Facilitated by the Royal Botanic Gardens, Kew. Published on the Internet; http //apps.kew.org/wcsp/ Retrieved 2014_8_1.
282. Govaerts, R., Newton, L. (2014). World Checklist of Xanthorrhoeaceae. Facilitated by the Royal Botanic Gardens, Kew. Published on the Internet; http //apps.kew.org/wcsp/ Retrieved 2014_8_1.
283. Govaerts, R., Cheek, M. (2014). World Checklist of Droseraceae. Facilitated by the Royal Botanic Gardens, Kew. Published on the Internet; http //apps.kew.org/wcsp/ Retrieved 2014_8_1.
284. Govaerts, R., Cheek, M. (2014). World Checklist of Drosophyllaceae. Facilitated by the Royal Botanic Gardens, Kew. Published on the Internet; http //apps.kew.org/wcsp/ Retrieved 2014_8_1.
285. Govaerts, R., Cheek, M. (2014). World Checklist of Ancistrocladaceae. Facilitated by the Royal Botanic Gardens, Kew. Published on the Internet; http //apps.kew.org/wcsp/ Retrieved 2014_8_1.

286.Govaerts, R., Sands, M.J.S. (2014). World Checklist of Begoniaceae. Facilitated by the Royal Botanic Gardens, Kew. Published on the Internet; http //apps.kew.org/wcsp/ Retrieved 2014_8_1.
287.Govaerts, R., Newman, M., Lock, J.M. (2014). World Checklist of Zingiberaceae. Facilitated by the Royal Botanic Gardens, Kew. Published on the Internet; http //apps.kew.org/wcsp/ Retrieved 2014_8_1.
288.Govaerts, R., Ruhsam, M., Andersson, L., Robbrecht, E., Bridson, D., Davis, A., Schanzer, I. & Sonké, B. (2014). World Checklist of Rubiaceae. Facilitated by the Royal Botanic Gardens, Kew. Published on the Internet; http //apps.kew.org/wcsp/ Retrieved 2014_8_1.
289.Govaerts, R., Sobral, M., Ashton, P., Barrie, F., Holst, B.K., Landrum, L.L., Matsumoto, K., Fernanda Mazine, F., Nic Lughadha, E., Proenca, C., Soares-Silva, L.H., Wilson, P.G. & Lucas, E. (2014). World Checklist of Myrtaceae. Facilitated by the Royal Botanic Gardens, Kew. Published on the Internet; http //apps.kew.org/wcsp/ Retrieved 2014_8_1.
290.Govaerts, R., Thomas, M. (2014). World Checklist of Ceratophyllaceae. Facilitated by the Royal Botanic Gardens, Kew. Published on the Internet; http //apps.kew.org/wcsp/ Retrieved 2014_8_1.
291.Govaerts, R., Jimenez-Mejias, P., Koopman, J., Simpson, D., Goetghebeur, P., Wilson, K., Egorova, T. & Bruhl, J. (2014). World Checklist of Cyperaceae. Facilitated by the Royal Botanic Gardens, Kew. Published on the Internet; http //apps.kew.org/wcsp/ Retrieved 2014_8_1.
292.Govaerts, R., Bernet, P., Kratochvil, K., Gerlach, G., Carr, G., Alrich, P., Pridgeon, A.M., Pfahl, J., Campacci, M.A., Holland Baptista, D., Tigges, H., Shaw, J., Cribb, P., Alex George, Kreuz, K. & Wood, J. (2014). World Checklist of Orchidaceae. Facilitated by the Royal Botanic Gardens, Kew. Published on the Internet; http //apps.kew.org/wcsp/ Retrieved 2014_8_1.
293.Govaerts, R., Berry, P.E. (2014). World Checklist of Rapateaceae. Facilitated by the Royal Botanic Gardens, Kew. Published on the Internet; http //apps.kew.org/wcsp/ Retrieved 2014_8_1.
294.Govaerts, R., Phillipson, P. (2014). World Checklist of Physenaceae. Facilitated by the Royal Botanic Gardens, Kew. Published on the Internet; http //apps.kew.org/wcsp/ Retrieved 2014_8_1.
295.Govaerts, R., Phillipson, P. (2014). World Checklist of Sphaerosepalaceae. Facilitated by the Royal Botanic Gardens, Kew. Published on the Internet; http //apps.kew.org/wcsp/ Retrieved 2014_8_1.
296.Govaerts, R., Green, P.S. (2014). World Checklist of Oleaceae. Facilitated by the Royal Botanic Gardens, Kew. Published on the Internet; http //apps.kew.org/wcsp/ Retrieved 2014_8_1.
297.Govaerts, R., Wilkin, P., Raz, L. & Téllez-Valdés, O. (2014). World Checklist of Dioscoreaceae. Facilitated by the Royal Botanic Gardens, Kew. Published on the Internet; http //apps.kew.org/wcsp/ Retrieved 2014_8_1.

298.Govaerts, R., Eriksson, R. (2014). World Checklist of Cyclanthaceae. Facilitated by the Royal Botanic Gardens, Kew. Published on the Internet; http //apps.kew.org/wcsp/ Retrieved 2014_8_1.
299.Govaerts, R., Eriksson, R. (2014). World Checklist of Basellaceae. Facilitated by the Royal Botanic Gardens, Kew. Published on the Internet; http //apps.kew.org/wcsp/ Retrieved 2014_8_1.
300.Govaerts, R., Faden, R. (2014). World Checklist of Commelinaceae. Facilitated by the Royal Botanic Gardens, Kew. Published on the Internet; http //apps.kew.org/wcsp/ Retrieved 2014_8_1.
301.Govaerts, R., Figlar, R., Nooteboom, H. & Spongberg, S. (2014). World Checklist of Magnoliaceae. Facilitated by the Royal Botanic Gardens, Kew. Published on the Internet; http //apps.kew.org/wcsp/ Retrieved 2014_8_1.
302.Govaerts, R., Saunders, R.M.K., Maas-van de Kamer, H., Maas-van de Kamer, P. & Zhang, D.X. (2014). World Checklist of Burmanniaceae. Facilitated by the Royal Botanic Gardens, Kew. Published on the Internet; http //apps.kew.org/wcsp/ Retrieved 2014_8_1.
303.Govaerts, R., Mori, S. (2014). World Checklist of Lecythidaceae. Facilitated by the Royal Botanic Gardens, Kew. Published on the Internet; http //apps.kew.org/wcsp/ Retrieved 2014_8_1.
304.Govaerts, R., Andrews, S., Coombes, A., Gilbert, M., Hunt, D., Nixon, K. & Thomas, M. (2014). World Checklist of Fagaceae. Facilitated by the Royal Botanic Gardens, Kew. Published on the Internet; http //apps.kew.org/wcsp/ Retrieved 2014_8_1.
305.Govaerts, R., Atkins, S. (2014). World Checklist of Verbenaceae. Facilitated by the Royal Botanic Gardens, Kew. Published on the Internet; http //apps.kew.org/wcsp/ Retrieved 2014_8_1.
306.Govaerts, R., Lammers, T.G. (2014). World Checklist of Campanulaceae. Facilitated by the Royal Botanic Gardens, Kew. Published on the Internet; http //apps.kew.org/wcsp/ Retrieved 2014_8_1.
307.Govaerts, R., Bopp, W. (2014). World Checklist of Betulaceae. Facilitated by the Royal Botanic Gardens, Kew. Published on the Internet; http //apps.kew.org/wcsp/ Retrieved 2014_8_1.
308.Govaerts, R., Harvey, Y., Jessup, L., Pennington, T.D. & Vink, W. (2014). World Checklist of Sapotaceae. Facilitated by the Royal Botanic Gardens, Kew. Published on the Internet; http //apps.kew.org/wcsp/ Retrieved 2014_8_1.
309.Govaerts, R. (2014). World Checklist of Acoraceae. Facilitated by the Royal Botanic Gardens, Kew. Published on the Internet; http //apps.kew.org/wcsp/ Retrieved 2014_8_1.
310.Govaerts, R. (2014). World Checklist of Aextoxicaceae. Facilitated by the Royal Botanic Gardens, Kew. Published on the Internet; http //apps.kew.org/wcsp/ Retrieved 2014_8_1.
311.Govaerts, R. (2014). World Checklist of Akaniaceae. Facilitated by the Royal Botanic Gardens, Kew. Published on the Internet; http //apps.kew.org/wcsp/ Retrieved 2014_8_1.
312.Govaerts, R. (2014). World Checklist of Alismataceae. Facilitated by the Royal Botanic Gardens, Kew. Published on the Internet; http //apps.kew.org/wcsp/ Retrieved 2014_8_1.

313.Govaerts, R. (2014). World Checklist of Alzateaceae. Facilitated by the Royal Botanic Gardens, Kew. Published on the Internet; http //apps.kew.org/wcsp/ Retrieved 2014_8_1.
314.Govaerts, R. (2014). World Checklist of Amborellaceae. Facilitated by the Royal Botanic Gardens, Kew. Published on the Internet; http //apps.kew.org/wcsp/ Retrieved 2014_8_1.
315.Govaerts, R. (2014). World Checklist of Anarthriaceae. Facilitated by the Royal Botanic Gardens, Kew. Published on the Internet; http //apps.kew.org/wcsp/ Retrieved 2014_8_1.
316.Govaerts, R. (2014). World Checklist of Aponogetonaceae. Facilitated by the Royal Botanic Gardens, Kew. Published on the Internet; http //apps.kew.org/wcsp/ Retrieved 2014_8_1.
317.Govaerts, R. (2014). World Checklist of Aralidiaceae. Facilitated by the Royal Botanic Gardens, Kew. Published on the Internet; http //apps.kew.org/wcsp/ Retrieved 2014_8_1.
318.Govaerts, R. (2014). World Checklist of Asteliaceae. Facilitated by the Royal Botanic Gardens, Kew. Published on the Internet; http //apps.kew.org/wcsp/ Retrieved 2014_8_1.
319.Govaerts, R. (2014). World Checklist of Asteropeiaceae. Facilitated by the Royal Botanic Gardens, Kew. Published on the Internet; http //apps.kew.org/wcsp/ Retrieved 2014_8_1.
320.Govaerts, R. (2014). World Checklist of Aucubaceae. Facilitated by the Royal Botanic Gardens, Kew. Published on the Internet; http //apps.kew.org/wcsp/ Retrieved 2014_8_1.
321.Govaerts, R. (2014). World Checklist of Austrobaileyaceae. Facilitated by the Royal Botanic Gardens, Kew. Published on the Internet; http //apps.kew.org/wcsp/ Retrieved 2014_8_1.
322.Govaerts, R. (2014). World Checklist of Avicenniaceae. Facilitated by the Royal Botanic Gardens, Kew. Published on the Internet; http //apps.kew.org/wcsp/ Retrieved 2014_8_1.
323.Govaerts, R. (2014). World Checklist of Balanopaceae. Facilitated by the Royal Botanic Gardens, Kew. Published on the Internet; http //apps.kew.org/wcsp/ Retrieved 2014_8_1.
324.Govaerts, R. (2014). World Checklist of Barbeuiaceae. Facilitated by the Royal Botanic Gardens, Kew. Published on the Internet; http //apps.kew.org/wcsp/ Retrieved 2014_8_1.
325.Govaerts, R. (2014). World Checklist of Barbeyaceae. Facilitated by the Royal Botanic Gardens, Kew. Published on the Internet; http //apps.kew.org/wcsp/ Retrieved 2014_8_1.
326.Govaerts, R. (2014). World Checklist of Bataceae. Facilitated by the Royal Botanic Gardens, Kew. Published on the Internet; http //apps.kew.org/wcsp/ Retrieved 2014_8_1.
327.Govaerts, R. (2014). World Checklist of Bixaceae. Facilitated by the Royal Botanic Gardens, Kew. Published on the Internet; http //apps.kew.org/wcsp/ Retrieved 2014_8_1.
328.Govaerts, R. (2014). World Checklist of Blandfordiaceae. Facilitated by the Royal Botanic Gardens, Kew. Published on the Internet; http //apps.kew.org/wcsp/ Retrieved 2014_8_1.
329.Govaerts, R. (2014). World Checklist of Boryaceae. Facilitated by the Royal Botanic Gardens, Kew. Published on the Internet; http //apps.kew.org/wcsp/ Retrieved 2014_8_1.
330.Govaerts, R. (2014). World Checklist of Bretschneideraceae. Facilitated by the Royal Botanic Gardens, Kew. Published on the Internet; http //apps.kew.org/wcsp/ Retrieved 2014_8_1.
331.Govaerts, R. (2014). World Checklist of Brunelliaceae. Facilitated by the Royal Botanic Gardens, Kew. Published on the Internet; http //apps.kew.org/wcsp/ Retrieved 2014_8_1.

332.Govaerts, R. (2014). World Checklist of Butomaceae. Facilitated by the Royal Botanic Gardens, Kew. Published on the Internet; http //apps.kew.org/wcsp/ Retrieved 2014_8_1.
333.Govaerts, R. (2014). World Checklist of Byblidaceae. Facilitated by the Royal Botanic Gardens, Kew. Published on the Internet; http //apps.kew.org/wcsp/ Retrieved 2014_8_1.
334.Govaerts, R. (2014). World Checklist of Cabombaceae. Facilitated by the Royal Botanic Gardens, Kew. Published on the Internet; http //apps.kew.org/wcsp/ Retrieved 2014_8_1.
335.Govaerts, R. (2014). World Checklist of Callitrichaceae. Facilitated by the Royal Botanic Gardens, Kew. Published on the Internet; http //apps.kew.org/wcsp/ Retrieved 2014_8_1.
336.Govaerts, R. (2014). World Checklist of Calycanthaceae. Facilitated by the Royal Botanic Gardens, Kew. Published on the Internet; http //apps.kew.org/wcsp/ Retrieved 2014_8_1.
337.Govaerts, R. (2014). World Checklist of Campynemataceae. Facilitated by the Royal Botanic Gardens, Kew. Published on the Internet; http //apps.kew.org/wcsp/ Retrieved 2014_8_1.
338.Govaerts, R. (2014). World Checklist of Canellaceae. Facilitated by the Royal Botanic Gardens, Kew. Published on the Internet; http //apps.kew.org/wcsp/ Retrieved 2014_8_1.
339.Govaerts, R. (2014). World Checklist of Cardiopteridaceae. Facilitated by the Royal Botanic Gardens, Kew. Published on the Internet; http //apps.kew.org/wcsp/ Retrieved 2014_8_1.
340.Govaerts, R. (2014). World Checklist of Caryocaraceae. Facilitated by the Royal Botanic Gardens, Kew. Published on the Internet; http //apps.kew.org/wcsp/ Retrieved 2014_8_1.
341.Govaerts, R. (2014). World Checklist of Casuarinaceae. Facilitated by the Royal Botanic Gardens, Kew. Published on the Internet; http //apps.kew.org/wcsp/ Retrieved 2014_8_1.
342.Govaerts, R. (2014). World Checklist of Centrolepidaceae. Facilitated by the Royal Botanic Gardens, Kew. Published on the Internet; http //apps.kew.org/wcsp/ Retrieved 2014_8_1.
343.Govaerts, R. (2014). World Checklist of Centroplacaceae. Facilitated by the Royal Botanic Gardens, Kew. Published on the Internet; http //apps.kew.org/wcsp/ Retrieved 2014_8_1.
344.Govaerts, R. (2014). World Checklist of Cephalotaceae. Facilitated by the Royal Botanic Gardens, Kew. Published on the Internet; http //apps.kew.org/wcsp/ Retrieved 2014_8_1.
345.Govaerts, R. (2014). World Checklist of Cercidiphyllaceae. Facilitated by the Royal Botanic Gardens, Kew. Published on the Internet; http //apps.kew.org/wcsp/ Retrieved 2014_8_1.
346.Govaerts, R. (2014). World Checklist of Circaeasteraceae. Facilitated by the Royal Botanic Gardens, Kew. Published on the Internet; http //apps.kew.org/wcsp/ Retrieved 2014_8_1.
347.Govaerts, R. (2014). World Checklist of Clethraceae. Facilitated by the Royal Botanic Gardens, Kew. Published on the Internet; http //apps.kew.org/wcsp/ Retrieved 2014_8_1.
348.Govaerts, R. (2014). World Checklist of Cochlospermaceae. Facilitated by the Royal Botanic Gardens, Kew. Published on the Internet; http //apps.kew.org/wcsp/ Retrieved 2014_8_1.

349.Govaerts, R. (2014). World Checklist of Columelliaceae. Facilitated by the Royal Botanic Gardens, Kew. Published on the Internet; http //apps.kew.org/wcsp/ Retrieved 2014_8_1.
350.Govaerts, R. (2014). World Checklist of Coriariaceae. Facilitated by the Royal Botanic Gardens, Kew. Published on the Internet; http //apps.kew.org/wcsp/ Retrieved 2014_8_1.
351.Govaerts, R. (2014). World Checklist of Cornaceae. Facilitated by the Royal Botanic Gardens, Kew. Published on the Internet; http //apps.kew.org/wcsp/ Retrieved 2014_8_1.
352.Govaerts, R. (2014). World Checklist of Corsiaceae. Facilitated by the Royal Botanic Gardens, Kew. Published on the Internet; http //apps.kew.org/wcsp/ Retrieved 2014_8_1.
353.Govaerts, R. (2014). World Checklist of Corynocarpaceae. Facilitated by the Royal Botanic Gardens, Kew. Published on the Internet; http //apps.kew.org/wcsp/ Retrieved 2014_8_1.
354.Govaerts, R. (2014). World Checklist of Crypteroniaceae. Facilitated by the Royal Botanic Gardens, Kew. Published on the Internet; http //apps.kew.org/wcsp/ Retrieved 2014_8_1.
355.Govaerts, R. (2014). World Checklist of Ctenolophonaceae. Facilitated by the Royal Botanic Gardens, Kew. Published on the Internet; http //apps.kew.org/wcsp/ Retrieved 2014_8_1.
356.Govaerts, R. (2014). World Checklist of Curtisiaceae. Facilitated by the Royal Botanic Gardens, Kew. Published on the Internet; http //apps.kew.org/wcsp/ Retrieved 2014_8_1.
357.Govaerts, R. (2014). World Checklist of Cymodoceaceae. Facilitated by the Royal Botanic Gardens, Kew. Published on the Internet; http //apps.kew.org/wcsp/ Retrieved 2014_8_1.
358.Govaerts, R. (2014). World Checklist of Cynomoriaceae. Facilitated by the Royal Botanic Gardens, Kew. Published on the Internet; http //apps.kew.org/wcsp/ Retrieved 2014_8_1.
359.Govaerts, R. (2014). World Checklist of Daphniphyllaceae. Facilitated by the Royal Botanic Gardens, Kew. Published on the Internet; http //apps.kew.org/wcsp/ Retrieved 2014_8_1.
360.Govaerts, R. (2014). World Checklist of Dasypogonaceae. Facilitated by the Royal Botanic Gardens, Kew. Published on the Internet; http //apps.kew.org/wcsp/ Retrieved 2014_8_1.
361.Govaerts, R. (2014). World Checklist of Degeneriaceae. Facilitated by the Royal Botanic Gardens, Kew. Published on the Internet; http //apps.kew.org/wcsp/ Retrieved 2014_8_1.
362.Govaerts, R. (2014). World Checklist of Didiereaceae. Facilitated by the Royal Botanic Gardens, Kew. Published on the Internet; http //apps.kew.org/wcsp/ Retrieved 2014_8_1.
363.Govaerts, R. (2014). World Checklist of Didymelaceae. Facilitated by the Royal Botanic Gardens, Kew. Published on the Internet; http //apps.kew.org/wcsp/ Retrieved 2014_8_1.
364.Govaerts, R. (2014). World Checklist of Dipentodontaceae. Facilitated by the Royal Botanic Gardens, Kew. Published on the Internet; http //apps.kew.org/wcsp/ Retrieved 2014_8_1.

365.Govaerts, R. (2014). World Checklist of Dipentodontaceae. Facilitated by the Royal Botanic Gardens, Kew. Published on the Internet; http //apps.kew.org/wcsp/ Retrieved 2014_8_1.
366.Govaerts, R. (2014). World Checklist of Doryanthaceae. Facilitated by the Royal Botanic Gardens, Kew. Published on the Internet; http //apps.kew.org/wcsp/ Retrieved 2014_8_1.
367.Govaerts, R. (2014). World Checklist of Ebenaceae. Facilitated by the Royal Botanic Gardens, Kew. Published on the Internet; http //apps.kew.org/wcsp/ Retrieved 2014_8_1.
368.Govaerts, R. (2014). World Checklist of Ecdeiocoleaceae. Facilitated by the Royal Botanic Gardens, Kew. Published on the Internet; http //apps.kew.org/wcsp/ Retrieved 2014_8_1.
369.Govaerts, R. (2014). World Checklist of Ephedraceae. Facilitated by the Royal Botanic Gardens, Kew. Published on the Internet; http //apps.kew.org/wcsp/ Retrieved 2014_8_1.
370.Govaerts, R. (2014). World Checklist of Eriocaulaceae. Facilitated by the Royal Botanic Gardens, Kew. Published on the Internet; http //apps.kew.org/wcsp/ Retrieved 2014_8_1.
371.Govaerts, R. (2014). World Checklist of Eupomatiaceae. Facilitated by the Royal Botanic Gardens, Kew. Published on the Internet; http //apps.kew.org/wcsp/ Retrieved 2014_8_1.
372.Govaerts, R. (2014). World Checklist of Flagellariaceae. Facilitated by the Royal Botanic Gardens, Kew. Published on the Internet; http //apps.kew.org/wcsp/ Retrieved 2014_8_1.
373.Govaerts, R. (2014). World Checklist of Garryaceae. Facilitated by the Royal Botanic Gardens, Kew. Published on the Internet; http //apps.kew.org/wcsp/ Retrieved 2014_8_1.
374.Govaerts, R. (2014). World Checklist of Ginkgoaceae. Facilitated by the Royal Botanic Gardens, Kew. Published on the Internet; http //apps.kew.org/wcsp/ Retrieved 2014_8_1.
375.Govaerts, R. (2014). World Checklist of Gnetaceae. Facilitated by the Royal Botanic Gardens, Kew. Published on the Internet; http //apps.kew.org/wcsp/ Retrieved 2014_8_1.
376.Govaerts, R. (2014). World Checklist of Gomortegaceae. Facilitated by the Royal Botanic Gardens, Kew. Published on the Internet; http //apps.kew.org/wcsp/ Retrieved 2014_8_1.
377.Govaerts, R. (2014). World Checklist of Gunneraceae. Facilitated by the Royal Botanic Gardens, Kew. Published on the Internet; http //apps.kew.org/wcsp/ Retrieved 2014_8_1.
378.Govaerts, R. (2014). World Checklist of Hanguanaceae. Facilitated by the Royal Botanic Gardens, Kew. Published on the Internet; http //apps.kew.org/wcsp/ Retrieved 2014_8_1.
379.Govaerts, R. (2014). World Checklist of Helwingiaceae. Facilitated by the Royal Botanic Gardens, Kew. Published on the Internet; http //apps.kew.org/wcsp/ Retrieved 2014_8_1.
380.Govaerts, R. (2014). World Checklist of Himantandraceae. Facilitated by the Royal Botanic Gardens, Kew. Published on the Internet; http //apps.kew.org/wcsp/ Retrieved 2014_8_1.
381.Govaerts, R. (2014). World Checklist of Hydatellaceae. Facilitated by the Royal Botanic Gardens, Kew. Published on the Internet; http //apps.kew.org/wcsp/ Retrieved 2014_8_1.
382.Govaerts, R. (2014). World Checklist of Hydrocharitaceae. Facilitated by the Royal Botanic Gardens, Kew. Published on the Internet; http //apps.kew.org/wcsp/ Retrieved 2014_8_1.

383.Govaerts, R. (2014). World Checklist of Hypoxidaceae. Facilitated by the Royal Botanic Gardens, Kew. Published on the Internet; http //apps.kew.org/wcsp/ Retrieved 2014_8_1.
384.Govaerts, R. (2014). World Checklist of Iridaceae. Facilitated by the Royal Botanic Gardens, Kew. Published on the Internet; http //apps.kew.org/wcsp/ Retrieved 2014_8_1.
385.Govaerts, R. (2014). World Checklist of Irvingiaceae. Facilitated by the Royal Botanic Gardens, Kew. Published on the Internet; http //apps.kew.org/wcsp/ Retrieved 2014_8_1.
386.Govaerts, R. (2014). World Checklist of Ixioliriaceae. Facilitated by the Royal Botanic Gardens, Kew. Published on the Internet; http //apps.kew.org/wcsp/ Retrieved 2014_8_1.
387.Govaerts, R. (2014). World Checklist of Joinvilleaceae. Facilitated by the Royal Botanic Gardens, Kew. Published on the Internet; http //apps.kew.org/wcsp/ Retrieved 2014_8_1.
388.Govaerts, R. (2014). World Checklist of Juncaceae (provided by I.O.P.I.). Facilitated by the Royal Botanic Gardens, Kew. Published on the Internet; http //apps.kew.org/wcsp/ Retrieved 2014_8_1.
389.Govaerts, R. (2014). World Checklist of Juncaginaceae. Facilitated by the Royal Botanic Gardens, Kew. Published on the Internet; http //apps.kew.org/wcsp/ Retrieved 2014_8_1.
390.Govaerts, R. (2014). World Checklist of Lanariaceae. Facilitated by the Royal Botanic Gardens, Kew. Published on the Internet; http //apps.kew.org/wcsp/ Retrieved 2014_8_1.
391.Govaerts, R. (2014). World Checklist of Liliaceae. Facilitated by the Royal Botanic Gardens, Kew. Published on the Internet; http //apps.kew.org/wcsp/ Retrieved 2014_8_1.
392.Govaerts, R. (2014). World Checklist of Limnocharitaceae. Facilitated by the Royal Botanic Gardens, Kew. Published on the Internet; http //apps.kew.org/wcsp/ Retrieved 2014_8_1.
393.Govaerts, R. (2014). World Checklist of Lowiaceae. Facilitated by the Royal Botanic Gardens, Kew. Published on the Internet; http //apps.kew.org/wcsp/ Retrieved 2014_8_1.
394.Govaerts, R. (2014). World Checklist of Luzuriagaceae. Facilitated by the Royal Botanic Gardens, Kew. Published on the Internet; http //apps.kew.org/wcsp/ Retrieved 2014_8_1.
395.Govaerts, R. (2014). World Checklist of Melanthiaceae. Facilitated by the Royal Botanic Gardens, Kew. Published on the Internet; http //apps.kew.org/wcsp/ Retrieved 2014_8_1.
396.Govaerts, R. (2014). World Checklist of Nartheciaceae. Facilitated by the Royal Botanic Gardens, Kew. Published on the Internet; http //apps.kew.org/wcsp/ Retrieved 2014_8_1.
397.Govaerts, R. (2014). World Checklist of Nothofagaceae. Facilitated by the Royal Botanic Gardens, Kew. Published on the Internet; http //apps.kew.org/wcsp/ Retrieved 2014_8_1.
398.Govaerts, R. (2014). World Checklist of Opiliaceae. Facilitated by the Royal Botanic Gardens, Kew. Published on the Internet; http //apps.kew.org/wcsp/ Retrieved 2014_8_1.
399.Govaerts, R. (2014). World Checklist of Pandanaceae. Facilitated by the Royal Botanic Gardens, Kew. Published on the Internet; http //apps.kew.org/wcsp/ Retrieved 2014_8_1.
400.Govaerts, R. (2014). World Checklist of Paracryphiaceae. Facilitated by the Royal Botanic Gardens, Kew. Published on the Internet; http //apps.kew.org/wcsp/ Retrieved 2014_8_1.
401.Govaerts, R. (2014). World Checklist of Philesiaceae. Facilitated by the Royal Botanic Gardens, Kew. Published on the Internet; http //apps.kew.org/wcsp/ Retrieved 2014_8_1.

402.Govaerts, R. (2014). World Checklist of Philydraceae. Facilitated by the Royal Botanic Gardens, Kew. Published on the Internet; http //apps.kew.org/wcsp/ Retrieved 2014_8_1.
403.Govaerts, R. (2014). World Checklist of Phrymaceae. Facilitated by the Royal Botanic Gardens, Kew. Published on the Internet; http //apps.kew.org/wcsp/ Retrieved 2014_8_1.
404.Govaerts, R. (2014). World Checklist of Phyllanthaceae. Facilitated by the Royal Botanic Gardens, Kew. Published on the Internet; http //apps.kew.org/wcsp/ Retrieved 2014_8_1.
405.Govaerts, R. (2014). World Checklist of Picrodendraceae. Facilitated by the Royal Botanic Gardens, Kew. Published on the Internet; http //apps.kew.org/wcsp/ Retrieved 2014_8_1.
406.Govaerts, R. (2014). World Checklist of Poaceae. Facilitated by the Royal Botanic Gardens, Kew. Published on the Internet; http //apps.kew.org/wcsp/ Retrieved 2014_8_1.
407.Govaerts, R. (2014). World Checklist of Pontederiaceae. Facilitated by the Royal Botanic Gardens, Kew. Published on the Internet; http //apps.kew.org/wcsp/ Retrieved 2014_8_1.
408.Govaerts, R. (2014). World Checklist of Posidoniaceae. Facilitated by the Royal Botanic Gardens, Kew. Published on the Internet; http //apps.kew.org/wcsp/ Retrieved 2014_8_1.
409.Govaerts, R. (2014). World Checklist of Potamogetonaceae (incomplete). Facilitated by the Royal Botanic Gardens, Kew. Published on the Internet; http //apps.kew.org/wcsp/ Retrieved 2014_8_1.
410.Govaerts, R. (2014). World Checklist of Putranjivaceae. Facilitated by the Royal Botanic Gardens, Kew. Published on the Internet; http //apps.kew.org/wcsp/ Retrieved 2014_8_1.
411.Govaerts, R. (2014). World Checklist of Ripogonaceae. Facilitated by the Royal Botanic Gardens, Kew. Published on the Internet; http //apps.kew.org/wcsp/ Retrieved 2014_8_1.
412.Govaerts, R. (2014). World Checklist of Ruppiaceae. Facilitated by the Royal Botanic Gardens, Kew. Published on the Internet; http //apps.kew.org/wcsp/ Retrieved 2014_8_1.
413.Govaerts, R. (2014). World Checklist of Saururaceae. Facilitated by the Royal Botanic Gardens, Kew. Published on the Internet; http //apps.kew.org/wcsp/ Retrieved 2014_8_1.
414.Govaerts, R. (2014). World Checklist of Scheuchzeriaceae. Facilitated by the Royal Botanic Gardens, Kew. Published on the Internet; http //apps.kew.org/wcsp/ Retrieved 2014_8_1.
415.Govaerts, R. (2014). World Checklist of Schisandraceae. Facilitated by the Royal Botanic Gardens, Kew. Published on the Internet; http //apps.kew.org/wcsp/ Retrieved 2014_8_1.
416.Govaerts, R. (2014). World Checklist of Smilacaceae. Facilitated by the Royal Botanic Gardens, Kew. Published on the Internet; http //apps.kew.org/wcsp/ Retrieved 2014_8_1.
417.Govaerts, R. (2014). World Checklist of Stemonaceae. Facilitated by the Royal Botanic Gardens, Kew. Published on the Internet; http //apps.kew.org/wcsp/ Retrieved 2014_8_1.
418.Govaerts, R. (2014). World Checklist of Stilbaceae. Facilitated by the Royal Botanic Gardens, Kew. Published on the Internet; http //apps.kew.org/wcsp/ Retrieved 2014_8_1.
419.Govaerts, R. (2014). World Checklist of Strelitziaceae. Facilitated by the Royal Botanic Gardens, Kew. Published on the Internet; http //apps.kew.org/wcsp/ Retrieved 2014_8_1.
420.Govaerts, R. (2014). World Checklist of Tecophilaeaceae. Facilitated by the Royal Botanic Gardens, Kew. Published on the Internet; http //apps.kew.org/wcsp/ Retrieved 2014_8_1.

421.Govaerts, R. (2014). World Checklist of Tetrachondraceae. Facilitated by the Royal Botanic Gardens, Kew. Published on the Internet; http //apps.kew.org/wcsp/ Retrieved 2014_8_1.
422.Govaerts, R. (2014). World Checklist of Thurniaceae. Facilitated by the Royal Botanic Gardens, Kew. Published on the Internet; http //apps.kew.org/wcsp/ Retrieved 2014_8_1.
423.Govaerts, R. (2014). World Checklist of Ticodendraceae. Facilitated by the Royal Botanic Gardens, Kew. Published on the Internet; http //apps.kew.org/wcsp/ Retrieved 2014_8_1.
424.Govaerts, R. (2014). World Checklist of Tofieldiaceae. Facilitated by the Royal Botanic Gardens, Kew. Published on the Internet; http //apps.kew.org/wcsp/ Retrieved 2014_8_1.
425.Govaerts, R. (2014). World Checklist of Typhaceae. Facilitated by the Royal Botanic Gardens, Kew. Published on the Internet; http //apps.kew.org/wcsp/ Retrieved 2014_8_1.
426.Govaerts, R. (2014). World Checklist of Velloziaceae. Facilitated by the Royal Botanic Gardens, Kew. Published on the Internet; http //apps.kew.org/wcsp/ Retrieved 2014_8_1.
427.Govaerts, R. (2014). World Checklist of Welwitschiaceae. Facilitated by the Royal Botanic Gardens, Kew. Published on the Internet; http //apps.kew.org/wcsp/ Retrieved 2014_8_1.
428.Govaerts, R. (2014). World Checklist of Xeronemataceae. Facilitated by the Royal Botanic Gardens, Kew. Published on the Internet; http //apps.kew.org/wcsp/ Retrieved 2014_8_1.
429.Govaerts, R. (2014). World Checklist of Zosteraceae. Facilitated by the Royal Botanic Gardens, Kew. Published on the Internet; http //apps.kew.org/wcsp/ Retrieved 2014_8_1.

The AgroAtlas Project

Interactive Agricultural Ecological Atlas of Russia and Neighboring Countries. Economic Plants and their Diseases, Pests and Weeds: The AgroAtlas Project began in 2003 and main collected economic plants and their decreases, pests and weeds in Russia and neiborhood countries. Each map represents a compilation of biological information, geographic distribution maps and environmental data, obtained from scientific research journals, scholarly publications, and biological collections of seeds, plants and insects. Each map is accompanied by a detailed description of the organism, a photograph or drawing and a list of references used. Available at: http://www.agroatlas.ru.

430.Agaev M. G., ed. 1988. Catalogue of the world collection of VIR. N. 468. Leningrad: VIR. 11-13 p. (In Russian)
431.Agaev M. G., ed. 1988. Main agricultural weeds in crops of the Leningrad Region. In: Catalogue of VIR world collection. N 468. Leningrad: VIR. 112 p. (In Russian)
432.Agaev M. G., ed. 1993. Useful weed plants in flora of the U.S.S.R. In: Catalogue of VIR world collection. N. 643. Leningrad: VIR. 160 p. (In Russian)
433.Anonymous. 1996. Weeds on sugar beet. Berlin: Hoehst Shering AgrEvo Gmbh. 479 p. (In Russian)
434.Artokhin K. S. 2004. Atlas Weed plants. Rostov-na-Donu. 144 p. (In Russian)

435.Basset, I.J. & Munro, D.B. 1986. The biology of Canadian weeds. 78. Solanum carolinense L. and Solanum rostratum Dunal. Canadian Journal of Plant Sciences. 66(10): 977-91.
436.Bassett, I. J. & Crompton, C. W. 1975. The biology of Canadian weeds. 11. Ambrosia artemisiifolia L. and A. psilostachya DC. Can. J. Plant Sci. 55: 463-476.
437.Bazdyrev G. I., Zotov L. I., Polin V. D. 2004. Weed plants and their control in modern agriculture. Moscow: MSKHA. 288 p. (In Russian)
438.Beilin I. G. 1967. Control of dodders and broomrapes. Moscow: Kolos. 88 p. (In Russian)
439.Belykh, A.G. 1974. Weed plants of Eastern Siberia and their control measures. Irkutsk: Irkutsk Agricultural Institute. p.25-7. (in Russian).
440.Beshanov A. V., Shilov G. E., Vydrina O. S. Weed control on fields of Non-Black Earth Region. Leningrad. 1983, pp. 18-19 (in Russian).
441.Bronshtein, Z.G. & Kabulov, D.T. 1961. Perspectives of biological method of Egyptian Broomrape control. In: Works of Samarkand State University. Samarkand: Samarkand State University, p. 73-89 (in Russian).
442.Buch T. G., Kachura N. N., Shvydkaya V. D., Andreeva E. R. 1981. Weeds of the Primorskii Territory and their control. Vladivostok: Dalnevostochnoe knizhnoe izdatelstvo. 256 pp. (In Russian)
443.Budantsev A. L., Lesiovskaya E. E., eds. 2001. Wild-growing useful plants of Russia. Saint Petersburg: SPHFA. 663 p. (In Russian)
444.Burygin V. A., Dzhangurazov F. H. 1975. Weed vegetation of the irrigated lands of Uzbekistan and biological bases of it control. In: Belov A.I., Burygin V. A., Kamilova F. G., eds. Weed vegetation of the irrigated lands of Central Asia. Proceedings of Tashkent agricultural institute. Tashkent: Tashkent agricultural institute. 1-7 p. (In Russian)
445.Butov A. Ya. 1958. Some regularity in distribution of species of the genus Cuscuta L. in Middle Asia. Works of V.I. Lenin Middle-Asian State University. Botany. Tashkent: SAGU. 57-61 p. (In Russian)
446.Chebotar, A.A., ed. 1989. Plants of steppes and limestone slopes, and weeds. Kishinev: Shtiintsa. 304 p. (in Russian).
447.Chepelev R. D. 1977. Weeds of Amur Region. Blagoveshchensk: Khabarovsk Publishing House. 72 p. (In Russian)
448.Cherepanov S. K. 1995. Vascular plants of Russia and adjacent states (of the former U.S.S.R). St. Petersburg: Mir I semya. 991 pp. (In Russian)
449.Chesalin G. A. 1975. Weeds and their control. Moscow: Kolos. 256 p. (In Russian)
450.Chikov P. S., ed. 1976. Atlas of areas and resources of herbs of the U.S.S.R. Moscow. 340 pp. (In Russian)
451.Deza M. I. 1989. Keys to weed plants of Kirghizia. Frunze, 204 pp. (in Russian). Diseases, weeds and pests of grain crops under Siberian condinion. Instruction manual. Krasnoobsk: SD RAAC. 1997. p. 46.
452.Dobrokhotov V. N. 1961. Seeds of weed plants. Moscow: Selkhozisdat, 414 p. (in Russian).

453.Dokuchaev V. M., ed. 1973. Green enemies. Weed plants of Stavropol' Territory and their control. Stavropol': Stavropol' Publishing House. 167 p. (In Russian)
454.Dorogostaiskaya E. V. 1972. Weed plants of the Far North of the U.S.S.R. Leningrad: Nauka. 172 pp. (In Russian)
455.Filatov A. M. 1983. Weed plants of Buryatia and their control. Irkutsk: Irkutsk SKHI. 62 p. (In Russian)
456.Fisyunov A. V. 1973. Dangerous weed. Zashchita rastenyi N.7: 45-46.
457.Fisyunov A. V. 1984. Handbook of weed control. Moscow: Kolos. 225 p.
458.Fisyunov A. V. 1984. Weed control reference book. Moscow: Kolos. 254 p. (In Russian)
459.Fisyunov A. V. 1984. Weeds. Moscow: Kolos. 320 pp. (In Russian)
460.Fisyunov A. V. 1984. Reference book on weed control. Moscow, Kolos. 255 p.
461.Fisyunov A. V., ed. 1984. Weed control reference book. Moscow: Kolos. 254 p. (In Russian)
462.Flora of the European part of the U.S.S.R. Vol. 1, Leningrad, 1974, p. 335 (in Russian).
463.Flora of the U.S.S.R. 1961. V.26. Ed.V.L.Komarov. Moscow-Leningrad, Ac.Sc.U.S.S.R. P.35-36.
464.Fomina Z. V. 1962. Weed plants of Buryatia and their control measures. Ulan-Ude: Buryatskoye knizhnoye izdatelstvo. 17 p. (In Russian)
465.Gaidashakin V. I., Makashvilli A.K, Yabrova V. S., Yaroshenko P. D. 1936. Weed plants of wet subtropics of the U.S.S.R and their control. N. 31. Sukhumi. 30p.
466.Geideman, T.S. & Nikolaeva, L.P. 1954. On Distribution of some quarantine weeds in Moldavian SSR. In: News of Moldavian branch of AS of the U.S.S.R. Kishinev: State publishing House of Moldova 1(15): p. 59-64 (in Russian).
467.Gismatov F. A., F. M. Khakov, R. V. Urazmetov. , 1967.Toward ecology of wild oats/ Scientific Conference on issues of biology, devoted to a 50-anniversary of the Great October Socialist revolution. — Ufa, p. 88-90.
468.Goloskokov V. P., ed. 1972. Illustrated keys to plants of Kazakhstan. V. 2. Alma-Ata: Nauka. 572 pp. (In Russian)
469.Golubintseva V. P. 1936. The specific weeds of Siberia. In: Reverdatto V.V., ed. Transactions of Biological Institute of Tomsk State University. V. 2. Tomsk: Tomsk State University. 170-227 p.
470.Grin'ko N.I., Titov A. Kh., Kvartin V. N., Semernikova A. I., Lapchenkov G. YA., Dyatlenko V. A. 1987. Weed plants and its control in the Rostov Region. Manual. Persianovka: Donskoi SKHI. 102 p. (In Russian)
471.Grossgeim A. A. 1949. Keys to plants of the Caucasus. Moscow: Sovetskaya nauka. 747 pp. (In Russian)
472.Grossgeim A. A. 1939. Flora of the Caucasus. V.1. Baku: AzFAN, p. 127 (in Russian).
473.Grossgeim A. A. 1949. Keys to plants of the Caucasus. Moscow: Sovetskaya nauka, 747 pp. (in Russian).
474.Grossgeim, A.A. 1945. Flora of the Caucasus. Baku: AN Azerb. SSR. V. 3: 32. (In Russian).

475.Gubanov I. A., Gubanov I. A., Kiseleva K. V., Novikov V. S., Tikhomirov V. N. 2002. The illustrated keys to plants of Middle Russia. Moscow: KMK. V. 1: 526. (In Russian)
476.Hulten E., Fries M. 1986. Atlas of North European Vascular Plants, North of the Tropic of Cancer. Konigstein. V. 1-3: 1172.
477.Jonsell, B., ed. 2000. Flora Nordica. Stockholm: Royal Swedish Academy of Sciences. V. 1: 254.
478.Jonsell, B., ed. 2000. Flora Nordica. V. 1. Lycopodiaceae-Polygonaceae. Stockholm: BTJ AB. 344 p.
479.Jonsell, B., ed. 2000. Flora Nordica. V. 1. Stockholm. 262-272 p. (In English)
480.Kabulov, D.T. & Mukumov Kh.M. 1965. About species composition of host-plants of Egyptian Broomrape (Orobanche aegyptiaca Pers.) in Middle Asia. Nauchnye doklady vysshei shkoly, Biologicheskie nauki (Moscow: Vysshaya shkola) 2: 111-116 (in Russian).
481.Karyagin I. I. ed. 1961. Flora of Azerbaijan. Baku: AN AzSSR. V.8. 690 pp. (in Russian).
482.Keller B. A., ed. 1934. Weed plants of the U.S.S.R. Leningrad: AN SSSR. V. 3: 67-69. (In Russian)
483.Keller B. A., ed. 1934. Weed plants of the U.S.S.R. Leningrad: AN SSSR. V. 2: 74-76. (In Russian)
484.Keller B. A., ed. 1934. Weed plants of the U.S.S.R. V. 1. Leningrad: AN SSSR. 324 p. (In Russian)
485.Keller B. A., ed. 1935. Weed plants of the U.S.S.R. V. 4. Moscow-Leningrad: AN SSSR. 416 p. (In Russian)
486.Keller, B.A., ed. 1934. Weed plants of the U.S.S.R. V. 2. Leningrad: AN SSSR. p.120. (in Russian).
487.Keller, B.A., ed. 1934. Weed plants of the U.S.S.R. V. 3. Leningrad: AN SSSR. 448 p. (in Russian).
488.Kharkevich S. S. (Ed.) 1992. Vascular plants of the Soviet Far East. Saint-Petersburg: Nauka. Vol. 6. 428 pp. (in Russian).
489.Kharkevich S. S., ed. 1991. Vascular plants of the Soviet Far East. V. 5. Saint Petersburg: Nauka. 392 pp. (In Russian)
490.Kharkevich, S.S., ed. 1987. Vascular plants of the Soviet Far East. V. 2. Leningrad: Nauka, 424 p. (in Russian).
491.Kharkhevich S. S., ed. 1988. Vascular plants of the Soviet Far East. Leningrad: Nauka. V. 3: 421. (In Russian)
492.Kolmakov P. P., Taskaeva A. G. 1985. Weed plants of Ural and their control measures. Chelyabinsk: uzhno-Uralskoye knizhnoye izdatelstvo. 19 p. (In Russian)
493.Komarov B. M. 1967. Keys to plants of Northern Tajikistan. Dushanbe: Donish. 400 pp. (In Russian)
494.Komarov V. L. & B. K. Shishkin, eds. 1936. Flora of the U.S.S.R. Moscow & Leningrad: AN SSSR. V. 6: 954. (In Russian)
495.Komarov V. L. (Ed.). 1934. Flora of the U.S.S.R. V.2. Leningrad: AN SSSR. P. 33 (in Russian).

496.Komarov V. L., ed. 1935. Flora of the U.S.S.R. Moscow-Leningrad: Publishing House Acad. Sci. U.S.S.R. V. 3: 501.
497.Komarov V. L., ed. 1936. Flora of the U.S.S.R. Moscow-Leningrad. V. 6: 386. Mal'tsev A.I. 1962. Weed vegetation of the U.S.S.R and its control. Leningrad-Moscow: Izd-vo sel'skokhozyaistvennoi literatury, zhurnalov i plakatov. 173 p. (In Russian)
498.Komarov V. L., ed. 1948. Flora of the U.S.S.R. V. 13. Moscow & Leningrad: AN SSSR, 588 p. (in Russian).
499.Komarov V. L., ed. 1958. Flora of the U.S.S.R. Moscow & Leningrad: AN SSSR. V. 23, 776 p. (in Russian).
500.Komarov, V. L., ed. 1959. Flora of the U.S.S.R. Moscow & Leningrad: AN SSSR. V. 25: 631 p. (in Russian).
501.Komarov, V.L & B. K. Shishkin, ed. 1936. Flora of the U.S.S.R. Moscow-Leningrad: Ac.Sc.U.S.S.R. V.6: 48.
502.Komarov, V.L. & Bush, N.A., eds. 1939. Flora of the U.S.S.R. V.8. Moscow-Leningrad: AN SSSR. 696 p. (in Russian).
503.Komarov, V.L. & Il'in, M.M., eds. 1934. Flora of the U.S.S.R. V. 1. Leningrad: AN SSSR. 302 p. (in Russian).
504.Komarov, V.L. & N. A. Bush, eds. 1939. Flora of the U.S.S.R. V. 8. Moscow-Leningrad: AN SSSR. 696 p. (In Russian)
505.Komarov, V.L. & Shishkin, B.K., eds. 1936. Flora of the U.S.S.R. V.6. Moscow-Leningrad: AN SSSR. p.95-6. (in Russian).
506.Komarov, V.L., ed. 1934. Flora of the U.S.S.R. V. 1. Moscow & Leningrad: AN SSSR. 302 p. (In Russian)
507.Komarov, V.L., ed. 1935. Flora of the U.S.S.R. Leningrad: Acad. Sci. U.S.S.R. V. 15: 462.
508.Komarov, V.L., ed. 1935. Flora of the U.S.S.R. V. 3. Leningrad: AN SSSR. 636 p. (In Russian)
509.Komarov, V.L., ed. 1936. Flora U.S.S.R. Moscow-Leningrad. V. 5: 649-650.
510.Komarov, V.L., ed. 1959. Flora of the U.S.S.R. V. 25. Moscow-Leningrad: AN SSSR. 557-558 p. (In Russian)
511.Komarov, V.L., ed. 1934-1964. Flora of the U.S.S.R. (in Russian).
512.Komarov, V.L., ed. 1936. Flora U.S.S.R. V. 5. Moscow; Leningrad: AN SSSR. 762 p. (in Russian).
513.Komarov, V.L., ed. 1959. Flora of the U.S.S.R. Moscow & Leningrad: AN SSSR. V. 25: 231. (In Russian)
514.Komarov, V.L., ed. 1959. Flora of the U.S.S.R. Moscow & Leningrad: AN SSSR. V. 26. (In Russian)
515.Korchagina V. A., Penchukov V. M. Morozov N.A., Smashevskaya G. A., Kolomiitsev F. B., Trubeeva A. I., Baranova M. M. 1972. Control of weeds in the Far East. Khabarovsk: Khabarovskoe Publishing House. 18 p. (In Russian)

516.Koroljeva IE, Vilchevskaya EV, Ruhovich DI. 2003. Digital Arable Land Map. Laboratory of Soil Information of the Dokuchaev Soil Institute, Moscow, Russia [Based on: Yanvareva LF. (ed.), Martynjuk KN.,
517.Kisileva NM. 1989. Map of Land Use, Faculty of Geography, Moscow State University, Moscow, Russia.].
518.Korovina O. N., ed. 1982. Weed plants of the Non-Chernozem Zone of Russia. In: Katalog mirovoi kollektsii VIR. Issue 338. Leningrad: VIR. 117 pp. (In Russian)
519.Korsmo E. 1933. Weed plants of modern farming. Moscow-Leningrad: State Publishing House of Kolchoz & Sovchoz Literature. 416 p. (In Russian)
520.Kostina V. A. 2001. Additions to the flora of Murmansk Region. Botanicheskii zhurnal, 86(10): 101-105. (In Russian)
521.Kott S. A. 1955. Weed plants and their control. Edition 2. Moscow: Selkhozgiz. 384 pp. (In Russian)
522.Krasavin V. D. 1989. Control of field-weed forms of millet. Vestnik sel'skokhozyaistvennoi nauki, 11: 154-155. (In Russian)
523.Krasnoborov I. M. & L. I. Malyshev, eds. 1992. Flora of Siberia. Novisibirsk: Nauka. V. 5: 312. (In Russian)
524.Krasnoborov I. M. (Ed.) 1997. Flora of Siberia. Novosibirsk: Nauka. Vol. 13. 472 pp. (in Russian).
525.Krasnoborov I. M., ed. 1984. Keys to plants of Tuva ASSR. Novosibirsk: Nauka. 336 pp. (In Russian)
526.Kuminov A. V., ed. 1973. Keys to plants of the Novosibirsk Region. Novosibirsk: Nauka. 368 pp. (In Russian)
527.Lenkov, P.V. 1932. Seeds of field weeds from European part of the U.S.S.R. Moscow-Leningrad: State Publishing House of Kolkhoz & Sovkhoz Literature. 320 p. (in Russian).
528.Maevskii P. F. 1954. Flora of middle belt of the European part of the U.S.S.R. Moscow & Leningrad: Selkhozgiz. 912 pp. (In Russian)
529.Maisuryan N. A., Atabekova A. I. 1978. Keys to seeds and fruits of weed plants. Moscow: Kolos. Edition 2. 288 pp. (in Russian)
530.Vasilchenko I.T. 1979. Keys to shoots of weed plants. Leningrad: Kolos, Edit. 2. 344 p. (in Russian).
531.Makarova V. A. 1955. Control of weeds. Rostov-na-Donu: Rostovskoe knizhnoe izdatel'stvo. P. 25-26 (in Russian).
532.Mal'tsev A.I. 1939. Atlas of major species of weed plants of the U.S.S.R. V. 2. Moscow-Leningrad: Selkhozgiz. 88 p. (In Russian)
533.Maltsev A. I. 1933. Weed vegetation in the U.S.S.R: A manual. 2 edition. Moscow: Kolhoz i sovkhoz literature. 296 p. (In Russian)
534.Maltsev A. I. 1937. Atlas of the main weed species of the U.S.S.R. V. 1. Moscow & Leningrad: Selkhozgiz. 168 p. (In Russian)
535.Maltsev A. I. 1939. Atlas of main weeds of the U.S.S.R. Moscow-Leningrad: Selkhozgiz. Part 2. 88 pp. (In Russian)

536.Maltsev A. I. 1962. Weed vegetation of the U.S.S.R and it control. Leningrad-Moscow: Publishing Office on Agricultural Literature, Journals and Posters. 172 p.
537.Maltsev A. I. Weed of a plant of the U.S.S.R / under educ. acad. B. A. Keller, etc. M. — L., 1935. T. 4. 414 p.
538.Mal'tsev A.I., 1930. Wild oats and cultivated oats. Leningrad, 522 pp.
539.Malyshev, L.I. & G. A. Peshkova, eds. 1979. Flora of the Central Siberia. V. 1. Novosibirsk. 536 pp. (In Russian)
540.Markova, S.A. 1971. Effect of physiological medium on growth and development of wormseed wallflower (Erysimum cheiranthoides L.). Bulletin MOIP. Sect. biol. 76(5): 86-95. (in Russian).
541.Maryushkina V. J. 1986. Ambrosia artemisiifolia and bases of its biological control. Kiev: Naukova dumka. 120 p. (in Russian).
542.McVean D. N. 1966. Ecology of Chondrilla juncea L. in south-eastern Australia. Journal of Ecology 54(2): 345-365. (In English)
543.Melnichuk O. S., Kovalivska G. M. 1972. Atlas of the most widespread weeds of the Ukraine. Kiev: Urozhay. 31 p. (In Ukrainian)
544.Minyaev N. A., ed. 1970. Synopsis of flora of Pskov Region. Leningrad: Leningrad University. 176 p. (in Russian).
545.Mordovets A. A. 1964. Heart-podded hoary cress (Cardaria draba (L.) Desv.) — dangerous agricultural weed in arid steppe. Botanicheskii zhurnal, 49(7): 1057-60. (In Russian)
546.Moskalenko, G.P. 2001. Quarantine weeds of Russia. Moscow: Rosgoskarantin. 280 pp. (in Russian).
547.Nikitin V. V. 1957. Weed vegetation of Turkmenia. Ashkhabad: AN Turkmen SSR. 581 pp. (In Russian)
548.Nikitin V. V. 1983. Weed plants of the U.S.S.R flora. Leningrad: Nauka. 454 pp. (In Russian)
549.Ovesnov S. A. 1997. Synopsis of flora of the Perm Region. Perm: Publishing house of the Perm University. 252 pp. (In Russian)
550.Peshkova, G.I., ed. 1996. Flora of Siberia. V. 10. Novosibirsk: Nauka. 254 pp. (In Russian)
551.Pilyugin N. 1953. Dodder and its control. Yaroslavl: Kolhoz i sovkhoz literature. 19 p. (In Russian)
552.Popov M. G., Busik V. V. 1966. Abstract of flora of coasts of Lake Baikal. Moscow-Leningrad: Nauka. 336 pp. (In Russian)
553.Popov, M.G. 1957. Flora of Central Siberia. Moscow-Leningrad: Acad. Sci. U.S.S.R. V. 1: 454. Prokudin, Yu.N., ed. 1987. Keys to vascular plants of Ukraine. Kiev: Naukova dumka, 547 pp. (In Russian)
554.Ramenskii L. G., Tsatsenkin I. A., Chizhikov O. N., Antipin N. A. 1956. Ecological evaluation of the fodder lands by vegetation cover. Moscow: Selkohzgiz. 472 pp. (In Russian)

555.Rubtsov N. I., ed. 1971. Wild useful plants of Crimea (short guide). Transactions of Nikitskii Botanical Garden. V. XLIX. Yalta: Nikitskii Botanical Garden. 280 p. (In Russian)
556.Rubtsov N. I., ed. 1972. Keys to vascular plants of the Crimea. Leningrad: Nauka. 552 pp. (In Russian)
557.Sal’nikova A.F., Zakharkin F. G. 1953. Main weed plants in the Far East and their control. Khabarovsk: Khabarovsk Publishing House. 56 p. (In Russian)
558.Samersov V. F., Padenov K. P., Soroka S. V. 2000. Weediness of crops in Byelorussia and methods of its reduction. Zashchita i karantin rastenii, 3: 20-22. (In Russian)
559.Shatalov T. A., ed. 1987. Weed plants and their control in Rostov Region. Rostov-na-Donu: Persianovka. 102 p. (In Russian)
560.Shishkin B. K. 1936. Flora of the U.S.S.R. Moscow-Leningrad: Ac. Sc. U.S.S.R. V. 6: 364.
561.Shishkin B. K. 1963. Botanical atlas. Moscow-Leningrad: publishing house of agricultural literature, journals and posters. 148 p.
562.Shishkin B. K. (Ed.). 1963. The botanical atlas. Moscow & Leningrad: Izdatel’stvo sel’skokhozyaistvennoi literatury i plakatov, p. 313 (in Russian).
563.Shishkin B. K., Bobrov E. G., eds. 1949. Flora of the U.S.S.R. Moscow & Leningrad: AN SSSR. V. 15: 742. (In Russian)
564.Shishkin B. K., ed. 1937. Flora of the U.S.S.R. Moscow-Leningrad: AN SSSR. V. 7: 713. (In Russian)
565.Shishkin, B.K. & Bobrov E. G., eds. 1961. Flora of the U.S.S.R. V.26. Moscow & Leningrad: AN SSSR. 940 p. (In Russian)
566.Shishkin, B.K. & Bobrov, E.G., ed. 1955. Flora of the U.S.S.R. V.22. Moscow-Leningrad: AN SSSR. 861 p. (in Russian).
567.Shishkin, B.K. & Bobrov, E.G., eds. 1948. Flora U.S.S.R. V. 13. Moscow; Leningrad: AN SSSR. 588 p. (in Russian).
568.Shishkin, B.K. & E. G. Bobrov, eds. 1949. Flora of the U.S.S.R. V. 14. Leningrad - Moscow: AN SSSR. 790 pp. (In Russian)
569.Shishkin, B.K. & S. V. Yusepchuk, eds. 1954. Flora of the U.S.S.R. V. 20. Moscow-Leningrad: AN SSSR. 556 p. (In Russian)
570.Shishkin, B.K., ed. 1950. Flora of the U.S.S.R. V. 16. Moscow-Leningrad: AN SSSR. 648 p. (In Russian)
571.Shishkin, B.K., ed. 1953. Flora of the U.S.S.R. V. 19. Moscow-Leningrad: AN SSSR. 752 p. (In Russian)
572.Shishkin, B.K., ed. 1954. Flora of the U.S.S.R. V. 21. Moscow-Leningrad: AN SSSR. 704 p. (In Russian)
573.Shishkin, B.K., ed. 1957. Flora of the Leningrad Region. V. 2. Leningrad: Leningrad University. 240 p. (In Russian)
574.Shishkin, B.K., ed. 1965. Flora of the Leningrad Region. V. 4. Leningrad: LGU. 356 p. (In Russian)

575.Shishkin, B.K., ed. 1958. Flora of the U.S.S.R. V. 23. Moscow; Leningrad: AN SSSR. 776 p. (in Russian).
576.Shishkin, B.K., ed. 1945. Flora U.S.S.R. V. 11. Moscow; Leningrad: AN SSSR. 432 p. (in Russian).
577.Shlyakova E. V. 1982. Keys to field weed plants of Non-Chernozem zone. Leningrad: Kolos. 208 pp. (In Russian)
578.Simonov, I. 1969. Weeds and their control. Sverdlovsk: Sredne-Ural`skoe Publishing House. 132 p. (in Russian).
579.Skorokhod V. 1951. Weeds of Donets Basin and their control. Makeevka: Stalin Publishing House. 72 pp. (In Russian)
580.Takhtadzhyan, A.L. & Fedorov, A.A. 1972. Flora of Erevan. Leningrad: Nauka. 396 pp. (in Russian).
581.Tanskii V. I., Levitin M. M., Ishkova T. I., Kondratenko V. I. 1998. Phytosanitary diagnostics in integrated managemant of cereals. Novozhilov K. V., editor. Compendium of Methodical Recommendations in Plant Protection. Saint Petersburg: VIZR. 5-55 p. (In Russian)
582.Novozhilov K. V., ed. Compendium of methodical recommendations in plant protection. St. Petersburg: VIZR. 5-55 p. (In Russian)
583.Tcherepanov S. K. Vascular of a plant of Russia and the adjacent states (within the limits of the former U.S.S.R). SPb, 1995. 991 p.
584.Tikhonova Z. E. 1937. Weeds and their control measures. Gorkii: Gorkovskij Region Publishing House. 97 p.
585.Tolmachev A. I., ed. 1974. Keys to higher plants of Sakhalin and Kurile islands. Leningrad: Nauka. 372 p. (In Russian)
586.Tolmachev A. I., ed. 1974. Keys to higher plants of Yakutia. Novosibirsk: Nauka. 544 pp. (In Russian)
587.Tolmachev, A.I., ed. 1974. Flora of North-East of the European part of the U.S.S.R. V. 1. Leningrad: Nauka. 276 pp. (In Russian)
588.Tolmachev, A.I., ed. 1976. Flora of North East of the European part of the U.S.S.R. Leningrad: Nauka. V. 2: 316 pp. (In Russian)
589.Tolmachev, A.I., ed. 1977. Flora of North-East of the European part of the U.S.S.R. Leningrad: Nauka. V. 4: 312. (In Russian)
590.Tshesalin G. A. 1975. Weeds and their control measures. Moscow: Kolos. 256 p.
591.Tsvelev N. N. 2000. A key to vascular plants of Northwestern Russia (Leningrad, Pskov and Novgorod Regions). Saint Petersburg. 352 p. (In Russian)
592.Tsvelev N. N. 1976. Cereals of the U.S.S.R. Leningrad: Nauka, 788 p. (In Russian) Tsvelev, N.N. 2000. Keys to the vascular plants of North-West Russia (Leningrad, Pskov and Novgorod Regions). Saint-Petersburg. 781 pp. (in Russian).
593.Ul'yanova T.N. 1998. Weed plants in flora of Russia and other countries of CIS. Saint Petersburg. 343 p. (In Russian)
594.Vasilchenko I. T., Pidotti O. A. 1975. Identification book on weed plants of regions of irrigated agriculture. Leningrad: Kolos. 376 p. (In Russian)

595.Vasileva L. I. & Matsenko A. E. 1964. Keys to weed plants of the Virgin Lands. Moscow & Leningrad: Nauka, 132 p. (In Russian)
596.Vershinin Yu.A., Fadeeva E. F. 2002. Common Millet in crops of grain cultures. Zashchita i karantin rastenii, 2: 47. (In Russian)
597.Veselovs'kii I.V., Lisenko A. K., Man'ko Yu.P. 1988. Atlas-synopsis of weeds. Kyiv: Urozhai. 72 p. (In Ukrainian)
598.Veselovskii, I.V., Lysenko, A. K., & Man'ko, Yu. P. 1988. The atlas and identification book on weeds. Kiev: Urozhai, 72 p. (in Ukrainian).
599.Visyulina O. D., ed. 1970. Weeds of Ukraine (reference-identification guide). Kiev: Naukova Dumka. 508 p. (In Ukrainian)
600.Vizgin, V.A., ed. 1961. Instructions on the Showy Sunflower control. Moscow: Sel'khozizdat. 16 pp. (In Russian)
601.Volkov A. N. 1935. Areas of distribution of the major weed plants in the U.S.S.R, Moscow & Leningrad. 152 p. (in Russian).
602.Vorobev D. P. et al. 1966. Keys to plants of Primorskii Territory and Amur Region. Moscow & Leningrad: Nauka, 492 pp. (in Russian).
603.Wapshere A. J., Hasan S., Wahba W. K., Caresche L. 1974. The ecology of Chondrilla juncea in the western Mediterranean. Journal of Applied Ecology. 11(2): 783-799. (In English)
604.Weeds and their control in the Rostov region. The textbook. 1987. Persianovka, p. 7-8.
605.Weeds in sugar beet. 1996. Berlin, Hoehst Schering AgrEvo GmbH, p. 374.
606.Wild bishop. 1999-2004. Weed database. Agricultural portal «Agro Mage»:
607.Yanchurkina A. A. 1975. New species of weeds in Kuibyshev region. In: Suburov V.V., editor. Species and introspecific classification of plants. Proc. of Leningrad Agricultural Institute. Leningrad-Pushkin: Leningrad Agricultural Institute. V. 267: 7-13.
608.Zakharenko V. A., Zakharenko A. V. 2004. Weed control. Zashchita i karantin rastenii, 4: 62-142. (In Russian)
609.Zhukovskii P. M. 1950. Cultural plants and their relatives. Moscow: Sovetskaya nauka, p. 500 (in Russian).
610.Zotova A. P. 1971. Weed plants and their control. Leningrad: Lenizdat. 86, 88 p. (In Russian)

Euro+Med Plantbase

integrates and critically evaluates information from Flora Europaea, Med-Checklist, the Flora of Macaronesia, and from regional and national floras and checklists from the area as well as additional taxonomic and floristic literature. This is complemented by the European taxa of several families taken from the World Checklist of Selected Plant Families and of the Leguminosae from the International Legume Database and Information Service ILDIS. By May 2016 it provides access to 194 plant families, corresponding to ca. 95 % of the European flora of vascular plants. For more informations: http://ww2.bgbm.org/EuroPlusMed/.

611.Euro+Med (2006-): Euro+Med PlantBase - the information resource for Euro-Mediterranean plant diversity. Published on the Internet http://ww2.bgbm.org/EuroPlusMed/ [accessed 05-2016].

USDA PLANTS Database

The PLANTS Database provides standardized information about the vascular plants, mosses, liverworts, hornworts, and lichens of the U.S. and its territories. For more information: https://plants.usda.gov/java/.

612. Complete PLANTS Checklist: https://plants.usda.gov/dl_all.html. Retrieved 03-2016.

India Biodiversity Portal

A unique repository of information on India's biodiversity. The Portal aims to provide open and free access to biodiversity information.

613.India Biodiversity Portal. https://indiabiodiversity.org/. Retrieved 04-2016.
614.IBIS-Flora Angiosperm Flora of India. https://flora.indianbiodiversity.org/. Retrieved 04-2016.

International Legume Database and Information Service (ILDIS)

ILDIS is an international project which maintains a database of plants in the family Fabaceae (Leguminosae) and provides services to scientists and other people interested in these plants, including this web-site for access to the database.

615.ILDIS: https://www.ildis.org/. Retrieved 12-2015.

National Specimen Information Infrasstructure (NSII)

*NSII The online sharing infrastructure has been building since 2003 including digital information of plant specimen, animal specimen, Fossil and rock specimen, Polar resources. R*etrieved 07-2015.

616.NSII: http://www.nsii.org.cn/2017/

Australia's virtual herbarium(AVH)

Herbarium specimens are important resource for research on the Australian flora and provide a permanent record of the occurrence of a species at a particular place and time. Australia's herbaria provides over seven million plant, algae and fungi specimens in Australia. For more information: ***http //avh.chah.org.au/***

617.The Council of Heads of Australasian Herbaria (1999-) Australia's Virtual Herbarium www.chah.gov.au/avh For more information: http://collections.ala.org.au/public/show/dr376.
618.Queensland Herbarium Records provided by Queensland Herbarium , accessed through ALA website. For more information: http://collections.ala.org.au/public/show/co49
619.State Herbarium of South Australia Records provided by State Herbarium of South Australia, accessed through ALA website. For more information: http://collections.ala.org.au/public/show/co48

620.Australian Tropical Herbarium Records provided by Australian Tropical Herbarium, accessed through ALA website. For more information: http://collections.ala.org.au/public/show/co17
621.Australian Tropical Herbarium Records provided by Australian Tropical Herbarium, accessed through ALA website. For more information: http://collections.ala.org.au/public/show/in67
622.University of New England Records provided by University of New England, accessed through ALA website. For more information: http://collections.ala.org.au/public/show/in65
623.The Royal Botanic Gardens & Domain Trust Records provided by The Royal Botanic Gardens & Domain Trust, accessed through ALA website. For more information: http://collections.ala.org.au/public/show/in50
624.Department of Land Resource Management Records provided by Department of Land Resource Management, accessed through ALA website. For more information: http://collections.ala.org.au/public/show/in87
625.Australia's Virtual Herbarium Records provided by Australia's Virtual Herbarium, accessed through ALA website. For more information: http://collections.ala.org.au/public/show/dp36
626.Tasmanian Museum and Art Gallery Records provided by Tasmanian Museum and Art Gallery, accessed through ALA website. For more information: http://collections.ala.org.au/public/show/in25
627.Tasmanian Herbarium Records provided by Tasmanian Herbarium, accessed through ALA website. For more information: http://collections.ala.org.au/public/show/co60
628.National Herbarium of New South Wales Records provided by National Herbarium of New South Wales, accessed through ALA website. For more information: http://collections.ala.org.au/public/show/co54
629.Department of Environment and Resource Management Records provided by Department of Environment and Resource Management, accessed through ALA website. For more information: http://collections.ala.org.au/public/show/in42
630.Australian National Herbarium Records provided by Australian National Herbarium, accessed through ALA website. For more information: http://collections.ala.org.au/public/show/co12
631.Department of Environment, Water and Natural Resources Records provided by Department of Environment, Water and Natural Resources, accessed through ALA website. For more information: http://collections.ala.org.au/public/show/in41
632.Northern Territory Herbarium Records provided by Northern Territory Herbarium, accessed through ALA website. For more information: http://collections.ala.org.au/public/show/co25
633.N.C.W. Beadle Herbarium Records provided by N.C.W. Beadle Herbarium, accessed through ALA website. For more information: http://collections.ala.org.au/public/show/co65
634.

635. The Council of Heads of Australasian Herbaria (1999-) Australia's Virtual Herbarium www.chah.gov.au/avh [Accessed <date of access>] For more information: http://collections.ala.org.au/public/show/dr376"
636. Royal Botanic Gardens Melbourne Records provided by Royal Botanic Gardens Melbourne, accessed through ALA website. For more information: http://collections.ala.org.au/public/show/in21
637. Centre for Australian National Biodiversity Research Records provided by Centre for Australian National Biodiversity Research, accessed through ALA website. For more information: http://collections.ala.org.au/public/show/in5
638. Western Australia, Department of Parks and Wildlife Records provided by Western Australia, Department of Parks and Wildlife, accessed through ALA website. For more information: http://collections.ala.org.au/public/show/in33
639. Western Australian Herbarium Records provided by Western Australian Herbarium, accessed through ALA website. For more information: http://collections.ala.org.au/public/show/co75
640. National Herbarium of Victoria Records provided by National Herbarium of Victoria, accessed through ALA website. For more information: http://collections.ala.org.au/public/show/co55
641. Queensland Herbarium Records provided by Queensland Herbarium , accessed through ALA website. For more information: http://collections.ala.org.au/public/show/co49
642. State Herbarium of South Australia Records provided by State Herbarium of South Australia, accessed through ALA website. For more information: http://collections.ala.org.au/public/show/co48
643. Australian Tropical Herbarium Records provided by Australian Tropical Herbarium, accessed through ALA website. For more information: http://collections.ala.org.au/public/show/co17
644. Australian Tropical Herbarium Records provided by Australian Tropical Herbarium, accessed through ALA website. For more information: http://collections.ala.org.au/public/show/in67
645. University of New England Records provided by University of New England, accessed through ALA website. For more information: http://collections.ala.org.au/public/show/in65
646. The Royal Botanic Gardens & Domain Trust Records provided by The Royal Botanic Gardens & Domain Trust, accessed through ALA website. For more information: http://collections.ala.org.au/public/show/in50
647. Department of Land Resource Management Records provided by Department of Land Resource Management, accessed through ALA website. For more information: http://collections.ala.org.au/public/show/in87
648. Australia's Virtual Herbarium Records provided by Australia's Virtual Herbarium, accessed through ALA website. For more information: http://collections.ala.org.au/public/show/dp36

649.Tasmanian Museum and Art Gallery Records provided by Tasmanian Museum and Art Gallery, accessed through ALA website. For more information: http://collections.ala.org.au/public/show/in25
650.Tasmanian Herbarium Records provided by Tasmanian Herbarium, accessed through ALA website. For more information: http://collections.ala.org.au/public/show/co60
651.National Herbarium of New South Wales Records provided by National Herbarium of New South Wales, accessed through ALA website. For more information: http://collections.ala.org.au/public/show/co54
652.Department of Environment and Resource Management Records provided by Department of Environment and Resource Management, accessed through ALA website. For more information: http://collections.ala.org.au/public/show/in42
653.Australian National Herbarium Records provided by Australian National Herbarium, accessed through ALA website. For more information: http://collections.ala.org.au/public/show/co12
654.Department of Environment, Water and Natural Resources Records provided by Department of Environment, Water and Natural Resources, accessed through ALA website. For more information: http://collections.ala.org.au/public/show/in41
655.Northern Territory Herbarium Records provided by Northern Territory Herbarium, accessed through ALA website. For more information: http://collections.ala.org.au/public/show/co25
656.N.C.W. Beadle Herbarium Records provided by N.C.W. Beadle Herbarium, accessed through ALA website. For more information: http://collections.ala.org.au/public/show/co65
657.Royal Botanic Gardens Melbourne Records provided by Royal Botanic Gardens Melbourne, accessed through ALA website. For more information: http://collections.ala.org.au/public/show/in21
658.Western Australia, Department of Parks and Wildlife Records provided by Western Australia, Department of Parks and Wildlife, accessed through ALA website. For more information: http://collections.ala.org.au/public/show/in33
659.Centre for Australian National Biodiversity Research Records provided by Centre for Australian National Biodiversity Research, accessed through ALA website. For more information: http://collections.ala.org.au/public/show/in5
660.Western Australian Herbarium Records provided by Western Australian Herbarium, accessed through ALA website. For more information: http://collections.ala.org.au/public/show/co75
661.National Herbarium of Victoria Records provided by National Herbarium of Victoria, accessed through ALA website. For more information: http://collections.ala.org.au/public/show/co55
662.Queensland Herbarium Records provided by Queensland Herbarium, accessed through ALA website. For more information: http://collections.ala.org.au/public/show/co49

663.State Herbarium of South Australia Records provided by State Herbarium of South Australia, accessed through ALA website. For more information: http://collections.ala.org.au/public/show/co48
664.Australian Tropical Herbarium Records provided by Australian Tropical Herbarium, accessed through ALA website. For more information: http://collections.ala.org.au/public/show/co17
665.Australian Tropical Herbarium Records provided by Australian Tropical Herbarium, accessed through ALA website. For more information: http://collections.ala.org.au/public/show/in67
666.University of New England Records provided by University of New England, accessed through ALA website. For more information: http://collections.ala.org.au/public/show/in65
667.The Royal Botanic Gardens & Domain Trust Records provided by The Royal Botanic Gardens & Domain Trust, accessed through ALA website. For more information: http://collections.ala.org.au/public/show/in50
668.Department of Land Resource Management Records provided by Department of Land Resource Management, accessed through ALA website. For more information: http://collections.ala.org.au/public/show/in87
669.Australia's Virtual Herbarium Records provided by Australia's Virtual Herbarium, accessed through ALA website. For more information: http://collections.ala.org.au/public/show/dp36
670.Tasmanian Museum and Art Gallery Records provided by Tasmanian Museum and Art Gallery, accessed through ALA website. For more information: http://collections.ala.org.au/public/show/in25
671.Tasmanian Herbarium Records provided by Tasmanian Herbarium, accessed through ALA website. For more information: http://collections.ala.org.au/public/show/co60
672.National Herbarium of New South Wales Records provided by National Herbarium of New South Wales, accessed through ALA website. For more information: http://collections.ala.org.au/public/show/co54
673.Department of Environment and Resource Management Records provided by Department of Environment and Resource Management, accessed through ALA website. For more information: http://collections.ala.org.au/public/show/in42
674.Australian National Herbarium Records provided by Australian National Herbarium, accessed through ALA website. For more information: http://collections.ala.org.au/public/show/co12
675.Department of Environment, Water and Natural Resources Records provided by Department of Environment, Water and Natural Resources, accessed through ALA website. For more information: http://collections.ala.org.au/public/show/in41
676.Northern Territory Herbarium Records provided by Northern Territory Herbarium, accessed through ALA website. For more information: http://collections.ala.org.au/public/show/co25

677.N.C.W. Beadle Herbarium Records provided by N.C.W. Beadle Herbarium, accessed through ALA website. For more information: http://collections.ala.org.au/public/show/co65
678.Royal Botanic Gardens Melbourne Records provided by Royal Botanic Gardens Melbourne, accessed through ALA website. For more information: http://collections.ala.org.au/public/show/in21
679.Western Australia, Department of Parks and Wildlife Records provided by Western Australia, Department of Parks and Wildlife, accessed through ALA website. For more information: http://collections.ala.org.au/public/show/in33
680.Centre for Australian National Biodiversity Research Records provided by Centre for Australian National Biodiversity Research, accessed through ALA website. For more information: http://collections.ala.org.au/public/show/in5
681.Western Australian Herbarium Records provided by Western Australian Herbarium, accessed through ALA website. For more information: http://collections.ala.org.au/public/show/co75
682.National Herbarium of Victoria Records provided by National Herbarium of Victoria, accessed through ALA website. For more information: http://collections.ala.org.au/public/show/co55
683.Queensland Herbarium Records provided by Queensland Herbarium , accessed through ALA website. For more information: http://collections.ala.org.au/public/show/co49
684.State Herbarium of South Australia Records provided by State Herbarium of South Australia, accessed through ALA website. For more information: http://collections.ala.org.au/public/show/co48
685.Australian Tropical Herbarium Records provided by Australian Tropical Herbarium, accessed through ALA website. For more information: http://collections.ala.org.au/public/show/co17
686.Australian Tropical Herbarium Records provided by Australian Tropical Herbarium, accessed through ALA website. For more information: http://collections.ala.org.au/public/show/in67
687.University of New England Records provided by University of New England, accessed through ALA website. For more information: http://collections.ala.org.au/public/show/in65
688.The Royal Botanic Gardens & Domain Trust Records provided by The Royal Botanic Gardens & Domain Trust, accessed through ALA website. For more information: http://collections.ala.org.au/public/show/in50
689.Department of Land Resource Management Records provided by Department of Land Resource Management, accessed through ALA website. For more information: http://collections.ala.org.au/public/show/in87
690.Australia's Virtual Herbarium Records provided by Australia's Virtual Herbarium, accessed through ALA website. For more information: http://collections.ala.org.au/public/show/dp36

691. Tasmanian Museum and Art Gallery Records provided by Tasmanian Museum and Art Gallery, accessed through ALA website. For more information: http://collections.ala.org.au/public/show/in25
692. Tasmanian Herbarium Records provided by Tasmanian Herbarium, accessed through ALA website. For more information: http://collections.ala.org.au/public/show/co60
693. National Herbarium of New South Wales Records provided by National Herbarium of New South Wales, accessed through ALA website. For more information: http://collections.ala.org.au/public/show/co54
694. Department of Environment and Resource Management Records provided by Department of Environment and Resource Management, accessed through ALA website. For more information: http://collections.ala.org.au/public/show/in42
695. Australian National Herbarium Records provided by Australian National Herbarium, accessed through ALA website. For more information: http://collections.ala.org.au/public/show/co12
696. Department of Environment, Water and Natural Resources Records provided by Department of Environment, Water and Natural Resources, accessed through ALA website. For more information: http://collections.ala.org.au/public/show/in41
697. Northern Territory Herbarium Records provided by Northern Territory Herbarium, accessed through ALA website. For more information: http://collections.ala.org.au/public/show/co25
698. N.C.W. Beadle Herbarium Records provided by N.C.W. Beadle Herbarium, accessed through ALA website. For more information: http://collections.ala.org.au/public/show/co65
699. The Council of Heads of Australasian Herbaria (1999-) Australia's Virtual Herbarium www.chah.gov.au/avh [Accessed <date of access>] For more information: http://collections.ala.org.au/public/show/dr376"
700. Royal Botanic Gardens Melbourne Records provided by Royal Botanic Gardens Melbourne, accessed through ALA website. For more information: http://collections.ala.org.au/public/show/in21
701. Western Australia, Department of Parks and Wildlife Records provided by Western Australia, Department of Parks and Wildlife, accessed through ALA website. For more information: http://collections.ala.org.au/public/show/in33
702. Centre for Australian National Biodiversity Research Records provided by Centre for Australian National Biodiversity Research, accessed through ALA website. For more information: http://collections.ala.org.au/public/show/in5
703. Western Australian Herbarium Records provided by Western Australian Herbarium, accessed through ALA website. For more information: http://collections.ala.org.au/public/show/co75
704. National Herbarium of Victoria Records provided by National Herbarium of Victoria, accessed through ALA website. For more information: http://collections.ala.org.au/public/show/co55

705. Queensland Herbarium Records provided by Queensland Herbarium , accessed through ALA website. For more information: http://collections.ala.org.au/public/show/co49
706. State Herbarium of South Australia Records provided by State Herbarium of South Australia, accessed through ALA website. For more information: http://collections.ala.org.au/public/show/co48
707. Australian Tropical Herbarium Records provided by Australian Tropical Herbarium, accessed through ALA website. For more information: http://collections.ala.org.au/public/show/co17
708. Australian Tropical Herbarium Records provided by Australian Tropical Herbarium, accessed through ALA website. For more information: http://collections.ala.org.au/public/show/in67
709. University of New England Records provided by University of New England, accessed through ALA website. For more information: http://collections.ala.org.au/public/show/in65
710. The Royal Botanic Gardens & Domain Trust Records provided by The Royal Botanic Gardens & Domain Trust, accessed through ALA website. For more information: http://collections.ala.org.au/public/show/in50
711. Department of Land Resource Management Records provided by Department of Land Resource Management, accessed through ALA website. For more information: http://collections.ala.org.au/public/show/in87
712. Australia's Virtual Herbarium Records provided by Australia's Virtual Herbarium, accessed through ALA website. For more information: http://collections.ala.org.au/public/show/dp36
713. Tasmanian Museum and Art Gallery Records provided by Tasmanian Museum and Art Gallery, accessed through ALA website. For more information: http://collections.ala.org.au/public/show/in25
714. Tasmanian Herbarium Records provided by Tasmanian Herbarium, accessed through ALA website. For more information: http://collections.ala.org.au/public/show/co60
715. National Herbarium of New South Wales Records provided by National Herbarium of New South Wales, accessed through ALA website. For more information: http://collections.ala.org.au/public/show/co54
716. Department of Environment and Resource Management Records provided by Department of Environment and Resource Management, accessed through ALA website. For more information: http://collections.ala.org.au/public/show/in42
717. Australian National Herbarium Records provided by Australian National Herbarium, accessed through ALA website. For more information: http://collections.ala.org.au/public/show/co12
718. Department of Environment, Water and Natural Resources Records provided by Department of Environment, Water and Natural Resources, accessed through ALA website. For more information: http://collections.ala.org.au/public/show/in41

719. Northern Territory Herbarium Records provided by Northern Territory Herbarium, accessed through ALA website. For more information: http://collections.ala.org.au/public/show/co25
720. N.C.W. Beadle Herbarium Records provided by N.C.W. Beadle Herbarium, accessed through ALA website. For more information: http://collections.ala.org.au/public/show/co65
721. The Council of Heads of Australasian Herbaria (1999-) Australia's Virtual Herbarium www.chah.gov.au/avh, For more information: http://collections.ala.org.au/public/show/dr376"
722. Royal Botanic Gardens Melbourne Records provided by Royal Botanic Gardens Melbourne, accessed through ALA website. For more information: http://collections.ala.org.au/public/show/in21
723. Western Australia, Department of Parks and Wildlife Records provided by Western Australia, Department of Parks and Wildlife, accessed through ALA website. For more information: http://collections.ala.org.au/public/show/in33
724. Centre for Australian National Biodiversity Research Records provided by Centre for Australian National Biodiversity Research, accessed through ALA website. For more information: http://collections.ala.org.au/public/show/in5
725. Western Australian Herbarium Records provided by Western Australian Herbarium, accessed through ALA website. For more information: http://collections.ala.org.au/public/show/co75
726. National Herbarium of Victoria Records provided by National Herbarium of Victoria, accessed through ALA website. For more information: http://collections.ala.org.au/public/show/co55
727. Queensland Herbarium Records provided by Queensland Herbarium , accessed through ALA website. For more information: http://collections.ala.org.au/public/show/co49
728. State Herbarium of South Australia Records provided by State Herbarium of South Australia, accessed through ALA website. For more information: http://collections.ala.org.au/public/show/co48
729. Australian Tropical Herbarium Records provided by Australian Tropical Herbarium, accessed through ALA website. For more information: http://collections.ala.org.au/public/show/co17
730. Australian Tropical Herbarium Records provided by Australian Tropical Herbarium, accessed through ALA website. For more information: http://collections.ala.org.au/public/show/in67
731. University of New England Records provided by University of New England, accessed through ALA website. For more information: http://collections.ala.org.au/public/show/in65
732. The Royal Botanic Gardens & Domain Trust Records provided by The Royal Botanic Gardens & Domain Trust, accessed through ALA website. For more information: http://collections.ala.org.au/public/show/in50

733. Department of Land Resource Management Records provided by Department of Land Resource Management, accessed through ALA website. For more information: http://collections.ala.org.au/public/show/in87
734. Australia's Virtual Herbarium Records provided by Australia's Virtual Herbarium, accessed through ALA website. For more information: http://collections.ala.org.au/public/show/dp36
735. Tasmanian Museum and Art Gallery Records provided by Tasmanian Museum and Art Gallery, accessed through ALA website. For more information: http://collections.ala.org.au/public/show/in25
736. Tasmanian Herbarium Records provided by Tasmanian Herbarium, accessed through ALA website. For more information: http://collections.ala.org.au/public/show/co60
737. National Herbarium of New South Wales Records provided by National Herbarium of New South Wales, accessed through ALA website. For more information: http://collections.ala.org.au/public/show/co54
738. Department of Environment and Resource Management Records provided by Department of Environment and Resource Management, accessed through ALA website. For more information: http://collections.ala.org.au/public/show/in42
739. Australian National Herbarium Records provided by Australian National Herbarium, accessed through ALA website. For more information: http://collections.ala.org.au/public/show/co12
740. Department of Environment, Water and Natural Resources Records provided by Department of Environment, Water and Natural Resources, accessed through ALA website. For more information: http://collections.ala.org.au/public/show/in41
741. Northern Territory Herbarium Records provided by Northern Territory Herbarium, accessed through ALA website. For more information: http://collections.ala.org.au/public/show/co25
742. N.C.W. Beadle Herbarium Records provided by N.C.W. Beadle Herbarium, accessed through ALA website. For more information: http://collections.ala.org.au/public/show/co65
743. The Council of Heads of Australasian Herbaria (1999-) Australia's Virtual Herbarium www.chah.gov.au/avh [Accessed <date of access>] For more information: http://collections.ala.org.au/public/show/dr376"
744. Royal Botanic Gardens Melbourne Records provided by Royal Botanic Gardens Melbourne, accessed through ALA website. For more information: http://collections.ala.org.au/public/show/in21
745. Western Australia, Department of Parks and Wildlife Records provided by Western Australia, Department of Parks and Wildlife, accessed through ALA website. For more information: http://collections.ala.org.au/public/show/in33
746. Centre for Australian National Biodiversity Research Records provided by Centre for Australian National Biodiversity Research, accessed through ALA website. For more information: http://collections.ala.org.au/public/show/in5

747. Western Australian Herbarium Records provided by Western Australian Herbarium, accessed through ALA website. For more information: http://collections.ala.org.au/public/show/co75
748. National Herbarium of Victoria Records provided by National Herbarium of Victoria, accessed through ALA website. For more information: http://collections.ala.org.au/public/show/co55
749. Queensland Herbarium Records provided by Queensland Herbarium , accessed through ALA website. For more information: http://collections.ala.org.au/public/show/co49
750. State Herbarium of South Australia Records provided by State Herbarium of South Australia, accessed through ALA website. For more information: http://collections.ala.org.au/public/show/co48
751. Australian Tropical Herbarium Records provided by Australian Tropical Herbarium, accessed through ALA website. For more information: http://collections.ala.org.au/public/show/co17
752. Australian Tropical Herbarium Records provided by Australian Tropical Herbarium, accessed through ALA website. For more information: http://collections.ala.org.au/public/show/in67
753. University of New England Records provided by University of New England, accessed through ALA website. For more information: http://collections.ala.org.au/public/show/in65
754. The Royal Botanic Gardens & Domain Trust Records provided by The Royal Botanic Gardens & Domain Trust, accessed through ALA website. For more information: http://collections.ala.org.au/public/show/in50
755. Department of Land Resource Management Records provided by Department of Land Resource Management, accessed through ALA website. For more information: http://collections.ala.org.au/public/show/in87
756. Australia's Virtual Herbarium Records provided by Australia's Virtual Herbarium, accessed through ALA website. For more information: http://collections.ala.org.au/public/show/dp36
757. Tasmanian Museum and Art Gallery Records provided by Tasmanian Museum and Art Gallery, accessed through ALA website. For more information: http://collections.ala.org.au/public/show/in25
758. Tasmanian Herbarium Records provided by Tasmanian Herbarium, accessed through ALA website. For more information: http://collections.ala.org.au/public/show/co60
759. National Herbarium of New South Wales Records provided by National Herbarium of New South Wales, accessed through ALA website. For more information: http://collections.ala.org.au/public/show/co54
760. Department of Environment and Resource Management Records provided by Department of Environment and Resource Management, accessed through ALA website. For more information: http://collections.ala.org.au/public/show/in42

761.Department of Environment, Water and Natural Resources Records provided by Department of Environment, Water and Natural Resources, accessed through ALA website. For more information: http://collections.ala.org.au/public/show/in41
762.Australian National Herbarium Records provided by Australian National Herbarium, accessed through ALA website. For more information: http://collections.ala.org.au/public/show/co12
763.Northern Territory Herbarium Records provided by Northern Territory Herbarium, accessed through ALA website. For more information: http://collections.ala.org.au/public/show/co25
764.N.C.W. Beadle Herbarium Records provided by N.C.W. Beadle Herbarium, accessed through ALA website. For more information: http://collections.ala.org.au/public/show/co65
765.The Council of Heads of Australasian Herbaria (1999-) Australia's Virtual Herbarium www.chah.gov.au/avh [Accessed <date of access>] For more information: http://collections.ala.org.au/public/show/dr376"
766.Royal Botanic Gardens Melbourne Records provided by Royal Botanic Gardens Melbourne, accessed through ALA website. For more information: http://collections.ala.org.au/public/show/in21
767.Western Australia, Department of Parks and Wildlife Records provided by Western Australia, Department of Parks and Wildlife, accessed through ALA website. For more information: http://collections.ala.org.au/public/show/in33
768.Centre for Australian National Biodiversity Research Records provided by Centre for Australian National Biodiversity Research, accessed through ALA website. For more information: http://collections.ala.org.au/public/show/in5
769.Western Australian Herbarium Records provided by Western Australian Herbarium, accessed through ALA website. For more information: http://collections.ala.org.au/public/show/co75
770.National Herbarium of Victoria Records provided by National Herbarium of Victoria, accessed through ALA website. For more information: http://collections.ala.org.au/public/show/co55
771.Queensland Herbarium Records provided by Queensland Herbarium , accessed through ALA website. For more information: http://collections.ala.org.au/public/show/co49
772.State Herbarium of South Australia Records provided by State Herbarium of South Australia, accessed through ALA website. For more information: http://collections.ala.org.au/public/show/co48
773.Australian Tropical Herbarium Records provided by Australian Tropical Herbarium, accessed through ALA website. For more information: http://collections.ala.org.au/public/show/co17
774.Australian Tropical Herbarium Records provided by Australian Tropical Herbarium, accessed through ALA website. For more information: http://collections.ala.org.au/public/show/in67

775.University of New England Records provided by University of New England, accessed through ALA website. For more information: http://collections.ala.org.au/public/show/in65
776.The Royal Botanic Gardens & Domain Trust Records provided by The Royal Botanic Gardens & Domain Trust, accessed through ALA website. For more information: http://collections.ala.org.au/public/show/in50
777.Department of Land Resource Management Records provided by Department of Land Resource Management, accessed through ALA website. For more information: http://collections.ala.org.au/public/show/in87
778.Australia's Virtual Herbarium Records provided by Australia's Virtual Herbarium, accessed through ALA website. For more information: http://collections.ala.org.au/public/show/dp36
779.Tasmanian Museum and Art Gallery Records provided by Tasmanian Museum and Art Gallery, accessed through ALA website. For more information: http://collections.ala.org.au/public/show/in25
780.Tasmanian Herbarium Records provided by Tasmanian Herbarium, accessed through ALA website. For more information: http://collections.ala.org.au/public/show/co60
781.National Herbarium of New South Wales Records provided by National Herbarium of New South Wales, accessed through ALA website. For more information: http://collections.ala.org.au/public/show/co54
782.Department of Environment and Resource Management Records provided by Department of Environment and Resource Management, accessed through ALA website. For more information: http://collections.ala.org.au/public/show/in42
783.Australian National Herbarium Records provided by Australian National Herbarium, accessed through ALA website. For more information: http://collections.ala.org.au/public/show/co12
784.Department of Environment, Water and Natural Resources Records provided by Department of Environment, Water and Natural Resources, accessed through ALA website. For more information: http://collections.ala.org.au/public/show/in41
785.Northern Territory Herbarium Records provided by Northern Territory Herbarium, accessed through ALA website. For more information: http://collections.ala.org.au/public/show/co25
786.N.C.W. Beadle Herbarium Records provided by N.C.W. Beadle Herbarium, accessed through ALA website. For more information: http://collections.ala.org.au/public/show/co65
787.The Council of Heads of Australasian Herbaria (1999-) Australia's Virtual Herbarium www.chah.gov.au/avh [Accessed <date of access>] For more information: http://collections.ala.org.au/public/show/dr376"
788.Royal Botanic Gardens Melbourne Records provided by Royal Botanic Gardens Melbourne, accessed through ALA website. For more information: http://collections.ala.org.au/public/show/in21

789. Western Australia, Department of Parks and Wildlife Records provided by Western Australia, Department of Parks and Wildlife, accessed through ALA website. For more information: http://collections.ala.org.au/public/show/in33
790. Centre for Australian National Biodiversity Research Records provided by Centre for Australian National Biodiversity Research, accessed through ALA website. For more information: http://collections.ala.org.au/public/show/in5
791. Western Australian Herbarium Records provided by Western Australian Herbarium, accessed through ALA website. For more information: http://collections.ala.org.au/public/show/co75
792. National Herbarium of Victoria Records provided by National Herbarium of Victoria, accessed through ALA website. For more information: http://collections.ala.org.au/public/show/co55
793. Queensland Herbarium Records provided by Queensland Herbarium , accessed through ALA website. For more information: http://collections.ala.org.au/public/show/co49
794. State Herbarium of South Australia Records provided by State Herbarium of South Australia, accessed through ALA website. For more information: http://collections.ala.org.au/public/show/co48
795. Australian Tropical Herbarium Records provided by Australian Tropical Herbarium, accessed through ALA website. For more information: http://collections.ala.org.au/public/show/co17
796. Australian Tropical Herbarium Records provided by Australian Tropical Herbarium, accessed through ALA website. For more information: http://collections.ala.org.au/public/show/in67
797. University of New England Records provided by University of New England, accessed through ALA website. For more information: http://collections.ala.org.au/public/show/in65
798. The Royal Botanic Gardens & Domain Trust Records provided by The Royal Botanic Gardens & Domain Trust, accessed through ALA website. For more information: http://collections.ala.org.au/public/show/in50
799. Department of Land Resource Management Records provided by Department of Land Resource Management, accessed through ALA website. For more information: http://collections.ala.org.au/public/show/in87
800. Australia's Virtual Herbarium Records provided by Australia's Virtual Herbarium, accessed through ALA website. For more information: http://collections.ala.org.au/public/show/dp36
801. Tasmanian Museum and Art Gallery Records provided by Tasmanian Museum and Art Gallery, accessed through ALA website. For more information: http://collections.ala.org.au/public/show/in25
802. Tasmanian Herbarium Records provided by Tasmanian Herbarium, accessed through ALA website. For more information: http://collections.ala.org.au/public/show/co60

803.National Herbarium of New South Wales Records provided by National Herbarium of New South Wales, accessed through ALA website. For more information: http://collections.ala.org.au/public/show/co54
804.Department of Environment and Resource Management Records provided by Department of Environment and Resource Management, accessed through ALA website. For more information: http://collections.ala.org.au/public/show/in42
805.Australian National Herbarium Records provided by Australian National Herbarium, accessed through ALA website. For more information: http://collections.ala.org.au/public/show/co12
806.Department of Environment, Water and Natural Resources Records provided by Department of Environment, Water and Natural Resources, accessed through ALA website. For more information: http://collections.ala.org.au/public/show/in41
807.Northern Territory Herbarium Records provided by Northern Territory Herbarium, accessed through ALA website. For more information: http://collections.ala.org.au/public/show/co25
808.N.C.W. Beadle Herbarium Records provided by N.C.W. Beadle Herbarium, accessed through ALA website. For more information: http://collections.ala.org.au/public/show/co65
809.The Council of Heads of Australasian Herbaria (1999-) Australia's Virtual Herbarium www.chah.gov.au/avh For more information: http://collections.ala.org.au/public/show/dr376"
810.Royal Botanic Gardens Melbourne Records provided by Royal Botanic Gardens Melbourne, accessed through ALA website. For more information: http://collections.ala.org.au/public/show/in21
811.Western Australia, Department of Parks and Wildlife Records provided by Western Australia, Department of Parks and Wildlife, accessed through ALA website. For more information: http://collections.ala.org.au/public/show/in33
812.Centre for Australian National Biodiversity Research Records provided by Centre for Australian National Biodiversity Research, accessed through ALA website. For more information: http://collections.ala.org.au/public/show/in5
813.Western Australian Herbarium Records provided by Western Australian Herbarium, accessed through ALA website. For more information: http://collections.ala.org.au/public/show/co75
814.National Herbarium of Victoria Records provided by National Herbarium of Victoria, accessed through ALA website. For more information: http://collections.ala.org.au/public/show/co55
815.Queensland Herbarium Records provided by Queensland Herbarium, accessed through ALA website. For more information: http://collections.ala.org.au/public/show/co49
816.State Herbarium of South Australia Records provided by State Herbarium of South Australia, accessed through ALA website. For more information: http://collections.ala.org.au/public/show/co48

817. Australian Tropical Herbarium Records provided by Australian Tropical Herbarium, accessed through ALA website. For more information: http://collections.ala.org.au/public/show/co17
818. Australian Tropical Herbarium Records provided by Australian Tropical Herbarium, accessed through ALA website. For more information: http://collections.ala.org.au/public/show/in67
819. University of New England Records provided by University of New England, accessed through ALA website. For more information: http://collections.ala.org.au/public/show/in65
820. The Royal Botanic Gardens & Domain Trust Records provided by The Royal Botanic Gardens & Domain Trust, accessed through ALA website. For more information: http://collections.ala.org.au/public/show/in50
821. Department of Land Resource Management Records provided by Department of Land Resource Management, accessed through ALA website. For more information: http://collections.ala.org.au/public/show/in87
822. Australia's Virtual Herbarium Records provided by Australia's Virtual Herbarium, accessed through ALA website. For more information: http://collections.ala.org.au/public/show/dp36
823. Tasmanian Museum and Art Gallery Records provided by Tasmanian Museum and Art Gallery, accessed through ALA website. For more information: http://collections.ala.org.au/public/show/in25
824. Tasmanian Herbarium Records provided by Tasmanian Herbarium, accessed through ALA website. For more information: http://collections.ala.org.au/public/show/co60
825. National Herbarium of New South Wales Records provided by National Herbarium of New South Wales, accessed through ALA website. For more information: http://collections.ala.org.au/public/show/co54
826. Department of Environment and Resource Management Records provided by Department of Environment and Resource Management, accessed through ALA website. For more information: http://collections.ala.org.au/public/show/in42
827. Australian National Herbarium Records provided by Australian National Herbarium, accessed through ALA website. For more information: http://collections.ala.org.au/public/show/co12
828. Department of Environment, Water and Natural Resources Records provided by Department of Environment, Water and Natural Resources, accessed through ALA website. For more information: http://collections.ala.org.au/public/show/in41
829. Northern Territory Herbarium Records provided by Northern Territory Herbarium, accessed through ALA website. For more information: http://collections.ala.org.au/public/show/co25
830. N.C.W. Beadle Herbarium Records provided by N.C.W. Beadle Herbarium, accessed through ALA website. For more information: http://collections.ala.org.au/public/show/co65

831.The Council of Heads of Australasian Herbaria (1999-) Australia's Virtual Herbarium www.chah.gov.au/avh [Accessed <date of access>] For more information: http://collections.ala.org.au/public/show/dr376"
832.Royal Botanic Gardens Melbourne Records provided by Royal Botanic Gardens Melbourne, accessed through ALA website. For more information: http://collections.ala.org.au/public/show/in21
833.Centre for Australian National Biodiversity Research Records provided by Centre for Australian National Biodiversity Research, accessed through ALA website. For more information: http://collections.ala.org.au/public/show/in5
834.Western Australia, Department of Parks and Wildlife Records provided by Western Australia, Department of Parks and Wildlife, accessed through ALA website. For more information: http://collections.ala.org.au/public/show/in33
835.Western Australian Herbarium Records provided by Western Australian Herbarium, accessed through ALA website. For more information: http://collections.ala.org.au/public/show/co75
836.National Herbarium of Victoria Records provided by National Herbarium of Victoria, accessed through ALA website. For more information: http://collections.ala.org.au/public/show/co55

The Global Biodiversity Information Facility (GBIF)

GBIF is an international open data infrastructure, funded by governments and contains 620, 573, 159 occurrences, 1, 643, 948 species, 29, 040 datasets and 812 data publishers currently. We retrieved 120, 000, 000 occurrences data for Angiosperm on 05-2014. For more information: www.gbif.org. *Below are the list of data sources.*

837.Levy Tacher, S. I. 1999. Contribución al conocimiento de la flora útil de la selva Lacandona. Conservation International México A.C. Bases de datos SNIB2010-CONABIO. Proyecto No. M002. México, D.F.
838.Jagiellonian University, Institute of Zoology: Weevils of Wales and England
839.GEO-Tag der Artenvielfalt: Spandau HBO
840.UK National Biodiversity Network: Glasgow Museums BRC - The Changing Flora of Glasgow: Orchid Dataset
841.GEO-Tag der Artenvielfalt: Rotes Steigle (Panzerübungplatz Böblingen)
842.Dickoré B. The Himalayan Uplands Plant database (HUP Version 1). Global Mountain Biodiversity Assessment GMBA
843.GEO-Tag der Artenvielfalt: Wälder bei Nordkirchen
844.Téllez Valdés, O. y J. Martínez. 2000. Base de datos de la flora de la Reserva de la Biosfera Chamela-Cuixmala, Jalisco, México. Universidad Nacional Autónoma de México. Instituto de Biología. Bases de datos SNIB2010-CONABIO proyecto No. L289. México, D.F.
845.GEO-Tag der Artenvielfalt: Feriendorf des Kreises Gedern (Ober-Seemen)
846.Centre d'estudis de la neu i de la muntanya d'Andorra (CENMA), Institut d'Estudis Andorrans: Fongs d'Andorra

847.Guardia, R. et al. (2007). Bases de dades de l'Herbari BCN http://www.ub.es/cedocbiv/bancdade.htm
848.Mwanga Mwanga I, Mergen P, Theeten F (2013) Herbarium Specimens of LW, CRSN, RMCA
849.GEO-Tag der Artenvielfalt: Dreilinden Gymnasium-Schulgelände
850.National Museum of Nature and Science, Japan: Herbarium Specimens of Tokushima Prefectural Museum, Japan
851.University of British Columbia Herbarium (UBC). http://www.biodiversity.ubc.ca/museum/herbarium/database.html. (consulted on [date]), http://www.biodiversity.ubc.ca/museum/herbarium/database.html
852.GEO-Tag der Artenvielfalt: Hochschule Zittau/Görlitz
853.UK National Biodiversity Network: Countryside Council for Wales - Phase 2 Lowland Grassland Survey of Wales
854.Schiebel, R et al. (2004): Coccolith counts of multinet M32/5_MSN979. doi:10.1594/PANGAEA.128640, In Supplement to: Schiebel, Ralf; Zeltner, A; Treppke, Ute F; Waniek, Joanna J; Bollmann, Jörg; Rixen, Tim; Hemleben, Christoph (2004): Distribution of diatoms, coccolithophores and planktic foraminifers along a trophic gradient during SW monsoon in the Arabian Sea. Marine Micropaleontology, 51(3-4), 345-371, doi:10.1016/j.marmicro.2004.02.001
855.UK National Biodiversity Network: Countryside Council for Wales - Pembrokeshire Marine Species Atlas
856.GEO-Tag der Artenvielfalt: Geschützter Landschaftsbestandteil - GLB "Troppach"
857.GEO-Tag der Artenvielfalt: Erzental (Oberotterbach)
858.Eguiarte Fruns, L. E. y G. R. Furnier. 1997. Niveles y patrones de variación genética del género Abies en México. Universidad Nacional Autónoma de México. Instituto de Ecología. Bases de datos SNIB2010-CONABIO proyecto No. B138. México, D.F.
859.GEO-Tag der Artenvielfalt: Landschaftspark St.Leonhard-Deisendorf
860.Head, MJ et al. (1989): Stratigraphic distribution of marine palynomorph species recorded for the Miocene of ODP Hole 105-645E (Table 1). doi:10.1594/PANGAEA.743927, Supplement to: Head, Martin J; Norris, Geoffrey; Mudie, Peta J (1989): Palynology and dinocyst stratigraphy of the Miocene in ODP Leg 105, Hole 645E, Baffin Bay. In: Srivastava, SP; Arthur, M; Clement, B; et al. (eds.), Proceedings of the Ocean Drilling Program, Scientific Results, College Station, TX (Ocean Drilling Program), 105, 467-514, doi:10.2973/odp.proc.sr.105.137.1989
861.GEO-Tag der Artenvielfalt: Natur erleben rund um den Seminarbauernhof Gut Hohenberg
862.GEO-Tag der Artenvielfalt: Schulhof Montessori Schule (Rotenburg / Wümme)
863.Siqueiros Beltrones, D. A. 1999. Estructura y variación geográfica de las asociaciones de diatomeas bentónicas de la Península de Baja California; Bahía de La Paz. Universidad Autónoma de Baja California Sur. Bases de datos SNIB2010-CONABIO proyecto No. H031. México D. F.
864.GEO-Tag der Artenvielfalt: Schulzentrum "Parc Hosingen"

865.GBIF-Spain: Herbario del Jardín Botánico-Histórico La Concepción: HBC
866.Institute of Botany, University of Hohenheim: Visual Plants (144.41.33.158) - Private collection of Florian Werner
867.GEO-Tag der Artenvielfalt: Deponie Klausdorf
868.GEO-Tag der Artenvielfalt: Wie viel Natur gibt es im Park?
869.Gyeryonsan Natural History Museum: Fossil (GNHM-FO)
870.GEO-Tag der Artenvielfalt: Gronau - auf der Suche nach dem Neunauge
871.GEO-Tag der Artenvielfalt: Zoo Frankfurt
872.National Institute of Genetics, ROIS: Herbarium Specimens of Museum of Nature and Human Activities, Hyogo Pref., Japan
873.Real Jardín Botánico (CSIC): Real Jardin Botanico: Dibujos de la Real Expedición Botánica del Nuevo Reino de Granada (1783-1816), dirigida por J.C. Mutis
874.Herbarium specimens of Bamboo collection Prafrance Générargue (BAMBO): Herbarium specimens
875.Oleoducto Bicentenario (2013). Rescate De Epífitas Oleoducto Bicentenario, Tramo Araguaney - Banadía (Siembra) 944. Registros, aportados por Alejandro Calderón (Publicador, Proveedor de los Metadatos, Proveedor de Contenido, Creador del Recurso). En linea, http://ipt.sibcolombia.net/sib/resource.do?r=epifitas_siembra, publicado el 08/05/2013.
876.GEO-Tag der Artenvielfalt: Fluss - Vielfalt
877.Villanueva Gutiérrez, R. Subproyecto Néctar: En: Pozo de la Tijera, M del C y S. Calmé. 2005. Uso y monitoreo de los recursos naturales en el Corredor Biológico Mesoamericano (áreas focales Xpujil-Zoh Laguna y Carrillo Puerto). El Colegio de la Frontera Sur. Unidad Chetumal. Bases de datos SNIB2010-CONABIO proyecto No.BJ002. México, D.F
878.Hannah, MJ et al. (2000): Marine palynomorph abundance estimates of sediment core CRP-2A (Fig. 2). doi:10.1594/PANGAEA.545144, Supplement to: Hannah, Mike J; Wilson, Gary S; Wrenn, John (2000): Oligocene and Miocene marine palynomorphs from CRP-2/2A, Victoria Land Basin, Antarctica. Terra Antartica, 7(4), 503-511, hdl:10013/epic.28256.d001
879.SPN - Service du Patrimoine naturel, Muséum national d'Histoire naturelle, Paris: Inventaire National du Patrimoine Naturel (I215) : Atlas de la flore de Lorraine
880.Museum and Institute of Zoology, Polish Academy of Sciences: Mollusca Collection
881.GEO-Tag der Artenvielfalt: Rechts des Inn Höhe Hofau Rosenheim
882.Herbarium der Regensburgischen Botanischen Gesellschaft von 1790 (REG): Flora exsiccata Bavarica, 1898-1930
883.Nijmegen Natural History Museum: Nijmegen Natural History Museum (NL) - Entomological Collection
884.Natural History Museum, University of Oslo: SWECO
885.University of Białystok, Institute of Biology: Herbarium of University of Białystok - Vascular Plants

886. Forest Research Institute, European Centre for Natural Forests: Coleoptera of Kozienice Forest
887. Vega Aviña, R. 2000. Catálogo y base de datos preliminar de la flora de Sinaloa. Universidad Autónoma de Sinaloa. Facultad de Agronomía. Bases de datos SNIB2010-CONABIO proyecto No. L057. México, D.F.
888. Masure, Edwige; Mascle, Jean; Lohmann, GP; Shipboard Scientific Party (2005): Range table from dinoflagellates, acritarchs and prasinophytes in ODP Hole 159-959D. doi:10.1594/PANGAEA.314484
889. UK National Biodiversity Network: Derbyshire Wildlife Trust - Derbyshire Wildlife Trust Diptera Records up to Dec 2011
890. GEO-Tag der Artenvielfalt: Tag der Artenvielfalt mit SchülerInnen des Leibniz-Gymnasiums in Neustadt a.d.W.
891. We acknowledge the BrACySol BRC (INRA Ploudaniel, France) that provided us with the plants that were used in this study.
892. GEO-Tag der Artenvielfalt: NABU-Fläche am Bösselhausener Weg
893. GEO-Tag der Artenvielfalt: Wedeler Au
894. Bárcenas Pazos, G. 2000. Banco de información sobre características tecnológicas de maderas mexicanas. Instituto de Ecología A. C. División de Vegetación y Flora. Base de datos SNIB2010-CONABIO proyecto No. K015. México, D.F.
895. Staatliches Museum für Naturkunde Stuttgart
896. GEO-Tag der Artenvielfalt: nazza
897. National Biodiversity Data Centre: Rocky Shore Macroalgae
898. Herbier Louis-Marie (QFA) from Université Laval. http://dx.doi.org/10.5886/3p8ltbg7 (accessed on [date])., doi:10.5886/3p8ltbg7
899. GEO-Tag der Artenvielfalt: BIRDRACE Gätkes Erben
900. Institute of Dendrology, Polish Academy of Sciences: Institute of Dendrology PAS, Flora of Sudety Mountains
901. Armonies, Werner (2010): Macrobenthos in surface sediments off Sylt collected during Heincke cruise HE275. Alfred Wegener Institute for Polar and Marine Research - Wadden Sea Station Sylt, doi:10.1594/PANGAEA.745724
902. National Museum of Nature and Science, Japan: Plant Specimens collections of the Kyushu UNniversity Museum
903. Chaisson, William P; Keigwin, Lloyd D; Rio, Domenico; Acton, Gary D; Shipboard Scientific Party (2005): Range table from planktonic foraminifers in ODP Hole 172-1060A. doi:10.1594/PANGAEA.315514
904. GEO-Tag der Artenvielfalt: Dörnberg
905. Fundacion Miguel Lillo Provider: Fundación Miguel Lillo - Colección Criptogámica
906. See Metadata record for details http://data.aad.gov.au/aadc/metadata/metadata_redirect.cfm?md=AMD/AU/AADC-00090
907. GEO-Tag der Artenvielfalt: Salzwiese Diekskiel

908.UK National Biodiversity Network: Greater Manchester Ecology Unit - Distribution of Species of Conservation Interest in Greater Manchester
909.GEO-Tag der Artenvielfalt: Tag der Artenvielfalt im Taubental
910.UK National Biodiversity Network: Scottish Natural Heritage - Survey of slender naiad, Najas flexilis, in Loch of Butterstone and Loch of Craiglush
911.UK National Biodiversity Network: Sussex Biodiversity Record Centre - Sussex Bryophyte Atlas Data edited by Howard Matcham
912.Naturalis Biodiversity Center: Natural History Museum Rotterdam
913.GEO-Tag der Artenvielfalt: DJH
914.Árboles y Arbustos Nativos para la Restauración Ecológica y Reforestación de México (IE-DF, UNAM)
915.ASTERACEAE, Portal UNIBIO, Instituto de Biología, Universidad Nacional Autónoma de México, http://www.unibio.unam.mx consultada el dd/mm/yy.
916.Hungarian Natural History Museum: Database of invertebrates collected in Mongolia
917.Natural History Museum, University of Oslo: Algae, Norwegian College of Fishery Science
918.Ruiz, T. (2005) Vascular collection herbarium online database in Extremadura
919.National Museum of Nature and Science, Japan: Plant Specimens of The Shimane Nature Museum of Mt. Sanbe
920.Caluff, M., Serguera, M., Sánchez, C., Morejón, R., Regalado, L., Hernández, A. et Daniel A. 2006. Pteridophyte collection online database.
921.Kyung Hee University Natural History Museum: Plant (NHMK-PL)
922.GEO-Tag der Artenvielfalt: Naturschutzstation Schmidsfelden
923.UK National Biodiversity Network: National Trust - Ickworth species data held by The National Trust.
924.GEO-Tag der Artenvielfalt: Schulgelände der Waldorfschule Vordertaunus in Oberursel
925.GEO-Tag der Artenvielfalt: Hainbachtal in Oelsnitz/V.
926.Manum, Svein B; Talwani, Manik; Udintsev, Gleb B (2005): Dinoflagellate abundance of Hole 38-338. doi:10.1594/PANGAEA.250510
927.Watkins, DK (1992): (Table 3) Calcareous nannofossil distribution in the Upper Cretaceous of ODP Site 120-750. doi:10.1594/PANGAEA.758718, In Supplement to: Watkins, David K (1992): Upper Cretaceous nannofossils from Leg 120, Kerguelen Plateau, Southern Ocean. In: Wise, SW; Schlich, R; et al. (eds.), Proceedings of the Ocean Drilling Program, Scientific Results, College Station, TX (Ocean Drilling Program), 120, 343-370, doi:10.2973/odp.proc.sr.120.180.1992
928.MEXUBR, Portal UNIBIO, Instituto de Biología, Universidad Nacional Autónoma de México, http://www.unibio.unam.mx consultada el dd/mm/yy.
929.BeBIF Provider: martius-munchen-infocomp
930.GEO-Tag der Artenvielfalt: Altholzparzelle Eilenriede Hannover
931.GEO-Tag der Artenvielfalt: Bayerische Donau - Blindheim Donaubrücke
932.GEO-Tag der Artenvielfalt: Leben auf einer Sturmfläche

933.University of Oulu: Bryophyta collection of the Botanical Museum of the University of Oulu
934.Senckenberg: Herbarium Senckenbergianum (FR)
935.OMEX Project Members; Lavaleye, Marc (2004): Benthic macrofauna abundance and biomass in surface sediment during cruise PLG95A. doi:10.1594/PANGAEA.207851
936.Centre for Genetic Resources, The Netherlands: Centre for Genetic Resources, the Netherlands, PGR passport data
937.Sturm H., O. Rangel. 1985. Ecologia de los Paramos Andinos: una visión preliminar integrada. Instituto de Ciencias Naturales - Universidad Nacional de Colombia. Bogota. 292p., n/a
938.GEO-Tag der Artenvielfalt: Paul-Gerhardt-Schule Dassel
939.GEO-Tag der Artenvielfalt: Grenzturm Hohen Neuendorf
940.GEO-Tag der Artenvielfalt: Westerwälder Umwelt- und Naturschutztag Limesgemeinde Hillscheid
941.Consortium of California Herbaria: Consortium of California Herbaria
942.GEO-Tag der Artenvielfalt: Trockenhang Greinhartsberg Edelfingen
943.Living marine legacy of Gwaii Haanas. II: Marine invertebrate baseline to 2000 and invertebrate-related management issues.
944.AIT Austrian Institute of Technology GmbH: The DNA and Sample Repository at AIT
945.Müller, S et al. (2008): Fig. 3. Pollen counts of the PG1756 core from Lake Billyakh. doi:10.1594/PANGAEA.717178, In Supplement to: Müller, Stefanie; Tarasov, Pavel E; Andreev, Andrei A; Diekmann, Bernhard (2009): Late Glacial to Holocene environments in the present-day coldest region of the Northern Hemisphere inferred from a pollen record of Lake Billyakh, Verkhoyansk Mts, NE Siberia. Climate of the Past, 5, 73-84, doi:10.5194/cp-5-73-2009
946.GEO-Tag der Artenvielfalt: Lebensraum Walram
947.Martínez, M. 1999. Flora acuática de Querétaro. Universidad Autónoma de Querétaro. Facultad de Ciencias Naturales. Bases de datos SNIB2010-CONABIO proyecto No. H076. México, D.F.
948.Field Museum: Field Museum of Natural History (Botany) Seed Plant Collection
949.GEO-Tag der Artenvielfalt: Schulwiese des Erich Kästner Gymnasiums (Laatzen)
950.GEO-Tag der Artenvielfalt: Besonderer Ort - besondere Natur: Die Mainzer Zitadelle
951.De la Torre Almaráz, R. 2001. Inventario fitopatológico de las especies vegetales dominantes en la región de Zapotitlán de las Salinas, Pue. Universidad Nacional Autónoma de México. Facultad de Estudios Superiores Iztacala. Bases de datos SNIB2010-CONABIO proyecto No. R013. México, D.F.
952.Tidal Creek Database, NOAA Oceans and Human Health Initiative, NOAA Hollings Marine Laboratory
953.Mokpo Museum of Natural History: Plant (MNHM-PL)
954.GEO-Tag der Artenvielfalt: Naturschutzgebiet Bausenberg
955.GEO-Tag der Artenvielfalt: Die Teiche im Britzer Garten

956.Dutch Foundation for Applied Water Research : Dutch Foundation for Applied Water Research (STOWA) - Limnodata Neerlandica
957.UK National Biodiversity Network: Shire Group of Internal Drainage Boards - Shire Group IDB species data 2004 to present
958.Hernández López, L. 1999. Las especies endémicas de plantas en el estado de Jalisco, su distribución y conservación. Universidad de Guadalajara. Centro Universitario de Ciencias Biológicas y Agropecuarias. Bases de datos SNIB2010-CONABIO proyecto No. J021. México, D.F.
959.UK National Biodiversity Network: EcoRecord - Bryophyte records held by EcoRecord for the Birmingham and the Black Country area collated prior to March 2013
960.GEO-Tag der Artenvielfalt: Ehmkendorf
961.Senckenberg: Collection Bernstein - SMF
962.Athlantic Botanical Garden: Jardín Botánico Atlántico, Gijón: JBAG-Laínz
963.MNHN - Museum national d'Histoire naturelle: Phanerogams herbarium specimens
964.GEO-Tag der Artenvielfalt: Aktion - Friedensburg Oberschule
965.UK National Biodiversity Network: Wiltshire and Swindon Biological Records Centre - Wiltshire & Swindon Site-based Survey Records
966.Senckenberg: Collection Pisces SMF
967.Senckenberg: Collection Crustacea SMF
968.GEO-Tag der Artenvielfalt: NSG Leist bei Ziegenhain
969.Natural History Museum, University of Oslo: Red list project inventory, bryophytes
970.GEO-Tag der Artenvielfalt: Schulgarten Paul-Schneider-Schule, Münster
971.Institute of Botany, University of Hohenheim: Visual Plants (144.41.33.158) - Private collection of Thorsten Kroemer
972.Equihua, C. 1999. Brioflora de la Reserva de Montes Azules, Chis.: Diversidad, biogeografía y depauperación por actividad humana. Universidad Nacional Autónoma de México. Instituto de Ecología. Bases de datos SNIB2010-CONABIO proyecto No. H285. México, D.F.
973.Repatriación de datos del Herbario de Arizona (ARIZ)
974.University of Alaska Museum, Earth Sciences Collection
975.Senckenberg Museum für Naturkunde Görlitz 1992 - (continuously updated): Vascular Plant Herbarium.
976.Ghana Biodiversity Information Facility (GhaBIF): University of Ghana - Ghana Herbarium
977.Jagiellonian University, Institute of Zoology: Chrysomelidae of the Carpathians
978.kakpo et Ganglo C.J., 2012. Caractéristiques structurales et écologiques des forêts de Bonou et d’Itchèdè au Sud-est du Bénin. Thèse d'ingénieur agronome, Faculté des Sciences Agronomiques/Université d'Abomey-Calavi. 39p
979.UK National Biodiversity Network: Woodland Condition Survey Group - Plants recorded during the non-SSSI woodland condition survey 2011
980.Lund Botanical Museum (LD): Lund Botanical Museum (LD)

981.UK National Biodiversity Network: Countryside Council for Wales - Welsh Peatland Invertebrate Survey (WPIS)
982.GEO-Tag der Artenvielfalt: Artenvielfalt im Rhein-Neckar-Raum
983.University of Hawaii: Joseph F. Rock Herbarium
984.Ernst-Moritz-Arndt-Universitaet: Floristic Databases of Mecklenburg-Pomerania - Higher Plants
985.Guardia, R. et al. (2011). Bases de dades de l'Herbari BCN http://www.ub.es/cedocbiv/bancdade.htm http://www.ub.es/cedocbiv/bancdade.htm
986.Brassicaceae of Canada
987.GEO-Tag der Artenvielfalt: Artenvielfalt am Schlern
988.GEO-Tag der Artenvielfalt: Landschaftsschutzgebiet Buchhorst2
989.GEO-Tag der Artenvielfalt: Artenvielfalt auf den Elbwiesen (Dessau)
990.GEO-Tag der Artenvielfalt: Töpinger Mischwald
991.GEO-Tag der Artenvielfalt: Flächennaturdenkmal Grubditz
992.González Elizondo, M. S., González Elizondo, M., Herrera Arrieta, Y. López Enriquez y J. A. Tena Flores. 1998. Base de datos sobre la flora de Durango. Instituto Politécnico Nacional. Centro Interdisciplinario de Investigación para el Desarrollo Integral Regional-Durango. Bases de datos SNIB2010-CONABIO proyecto No. P005. México, D.F.
993.GEO-Tag der Artenvielfalt: NABU-Projekt (Osterode am Harz) Südharzer Gipskarst
994.Pfannkuchen, Martin; Godrian, Jelena; Marić Pfannkuchen, Daniela (Ed.) 2012 - (continuously updated): DNA samples of the CIM DNA bank at the CIM (Center for Marine Research, Rovinj, Institute Ruđer Bošković).
995.BeBIF Provider: Central African Plants
996.UK National Biodiversity Network: Thames Valley Environmental Records Centre - Local Wildlife Site Surveys Oxfordshire
997.GEO-Tag der Artenvielfalt: Kinderbauernhof Pinke-Panke
998.GEO-Tag der Artenvielfalt: Lerchenauer Baggersee (München)
999.GEO-Tag der Artenvielfalt: renaturierter Main (Kemmern bei Bamberg)
1000. GEO-Tag der Artenvielfalt: Haslauer Moor
1001. Instituto Amazónico de Investigaciones Científicas - SINCHI (2012 -). Herbario Amazónico Colombiano, 56155 Registros, Aportados por Mantilla-Cárdenas LM (Publicador, Creador del Recurso, Proveedor de los Metadatos), En línea, http://ipt.sibcolombia.net/sinchi/, Versión X.0 (actualizado por última vez el 30/11/2012)., http://ipt.sibcolombia.net/sinchi/
1002. Biologische Anstalt Helgoland in the Foundation Alfred-Wegener Institute for Polar and Marine Research: AWI-Herbarium Marine Macroalgae
1003. Marie-Victorin Herbarium (MT) from University of Montreal Biodiversity Centre. http://dx.doi.org/10.5886/rzav8bu2 (accessed on [date])., doi:10.5886/rzav8bu2
1004. GEO-Tag der Artenvielfalt: Bachpatenschaft Beeke
1005. Eldrett, JS et al. (2004): Distribution of dinoflagellate cysts in Eocene-Oligocene sediments of ODP Hole 104-643A. doi:10.1594/PANGAEA.758803, In Supplement to: Eldrett, James S; Harding, Ian C; Firth, John V; Roberts, Andrew P (2004):

Magnetostratigraphic calibration of Eocene-Oligocene dinoflagellate cyst biostratigraphy from the Norwegian-Greenland Sea. Marine Geology, 204(1-2), 91-127, doi:10.1016/S0025-3227(03)00357-8
1006. GEO-Tag der Artenvielfalt: Wiese und Bach beim Unterbecken (Markersbach)
1007. Guardia, R. et al. (2007). Bases de dades de l'Herbari BCN http://www.ub.es/cedocbiv/bancdade.htm
1008. GEO-Tag der Artenvielfalt: BÜG
1009. Museum für Naturkunde Berlin: Staatliches Naturhistorisches Museum Braunschweig - Coleoptera Collection
1010. Jyväskylä University Museum - The Section of Natural Sciences: Bryophyte collection of Jyväskylä University Museum
1011. National Museum in Prague: Botanical collection of the National Museum
1012. Hernández Sandoval, L. G. 1998. Diversidad florística y endemismo en la Reserva de la Biósfera El Cielo, Tamaulipas, México. Universidad Autónoma de Tamaulipas. Instituto de Ecología Aplicada. Bases de datos SNIB2010-CONABIO proyecto No. P023. México, D.F.
1013. Botanical Research Institute of Texas: Andes to Amazon Biodiversity Program
1014. UK National Biodiversity Network: Dorset Environmental Records Centre - Dorset SSSI Species Records 1952 - 2004 (Natural England)
1015. Pando, F. et al. (2003). MA Cryptogamic collections online databases. http://www.rjb.csic.es/herbario/crypto/crydb.htm. (date when consulted)
1016. UK National Biodiversity Network: Countryside Council for Wales - Rare Flowering Plant and Fern Data
1017. Árboles de la Península de Yucatán, Flora del Distrito de Tehuantepec, Oaxaca y Familia Asteraceae en México (IBUNAM)
1018. UK National Biodiversity Network: Nature Locator - PlantTracker data from 2012 onwards
1019. GEO-Tag der Artenvielfalt: Naturgarten Langenholtensen
1020. Arizona State University, Global Institute for Sustainability: Arizona State University Vascular Plant Herbarium
1021. Wendler, J et al. (2002): Calcareous dinocysts in ODP Hole 80-550B, part 1. doi:10.1594/PANGAEA.66909, In Supplement to: Wendler, Jens; Gräfe, Kai-Uwe; Willems, Helmut (2002): Palaeoecology of calcareous dinoflagellate cysts in the mid-Cenomanian Boreal Realm - Implications for the reconstruction of palaeoceanography of the NW European shelf sea. Cretaceous Research, 23, 213-229, doi:10.1006/cres.2002.0311
1022. Regalado, L., Lóriga J., Morejón, R., Hechavarría, L., Fuentes, I., Hernández A., Daniel, A., Caluff, M., Ventosa, I., Vale, A. et Echevarría R. 2006. Pteridophyte collection online database.
1023. GEO-Tag der Artenvielfalt: Wiesen in der Grünen Mitte
1024. Schiebel, R et al. (2004): Coccolith counts of multinet SO119_MSN1295. doi:10.1594/PANGAEA.128657, In Supplement to: Schiebel, Ralf; Zeltner, A; Treppke,

Ute F; Waniek, Joanna J; Bollmann, Jörg; Rixen, Tim; Hemleben, Christoph (2004): Distribution of diatoms, coccolithophores and planktic foraminifers along a trophic gradient during SW monsoon in the Arabian Sea. Marine Micropaleontology, 51(3-4), 345-371, doi:10.1016/j.marmicro.2004.02.001
1025. Korea Institute of Science and Technology Information: kisti_aquap
1026. University of Colorado Museum of Natural History: Specimen Database of Colorado Vascular Plants
1027. Museum Victoria: Museum Victoria provider for OZCAM
1028. GEO-Tag der Artenvielfalt: Ohemoor (Hamburg/Norderstedt)
1029. GEO-Tag der Artenvielfalt: Bizzenbachtal (Wehrheim/Taunus)
1030. GEO-Tag der Artenvielfalt: BIRDRACE Oostfreesland Bird-Lopers
1031. Abisaí Josué García Mendoza, FLORAOAXACA, Portal UNIBIO, Instituto de Biología, Universidad Nacional Autónoma de México, http://www.unibio.unam.mx consultada el dd/mm/yy.
1032. University of Kansas Biodiversity Institute Invertebrate Zoology Collection
1033. UK National Biodiversity Network: Merseyside BioBank - Merseyside BioBank Active Naturalists (unverified)
1034. GEO-Tag der Artenvielfalt: Geo-Tag der Artenvielfalt Süßen Hornwiesen-Grundschule
1035. GEO-Tag der Artenvielfalt: Artenfülle um das Schalkenmehrener Maar
1036. Masure, E (1988): (Figure 2) Distribution chart of dinoflagellate cysts of Cretaceous age from ODP Hole 103-638C. doi:10.1594/PANGAEA.743448, In Supplement to: Masure, Edwige (1988): Berriasian to Aptian dinoflagellate cysts from the Galicia margin, offshore Spain, Sites 638 and 639, ODP Leg 103. In: Boillot, G; Winterer, EL; et al. (eds.), Proceedings of the Ocean Drilling Program, Scientific Results, College Station, TX (Ocean Drilling Program), 103, 433-444, doi:10.2973/odp.proc.sr.103.183.1988
1037. GEO-Tag der Artenvielfalt: Oschenberg, NO von Bayreuth-Laineck
1038. Museum of Biological Diversity, The Ohio State University: Ohio State Acarology Laboratory (OSAL), Ohio State University
1039. GEO-Tag der Artenvielfalt: Uferzone Wipper (Biesenrode)
1040. GEO-Tag der Artenvielfalt: Schulgelände Städtisches Gymnasium
1041. GEO-Tag der Artenvielfalt: Stadtgebiet (Dannenberg)
1042. GEO-Tag der Artenvielfalt: Landschaftsschutzgebiet Buchhorst 4
1043. Oosting, AM et al. (2006): (Table 3) Distribution chart of dinoflagellate cysts and acritarchs from DSDP Hole 27-263. doi:10.1594/PANGAEA.747719, In Supplement to: Oosting, AM; Leereveld, H; Dickens, Gerald Roy; Henderson, RA; Brinkhuis, Henk (2006): Correlation of Barremian-Aptian (mid-Cretaceous) dinoflagellate cyst assemblages between the Tethyan and Austral realms. Cretaceous Research, 27(6), 792-813, doi:10.1016/j.cretres.2006.03.012
1044. Jagiellonian University, Institute of Zoology: Carabidae of Poland
1045. GEO-Tag der Artenvielfalt: Waldgebiet Todtnau- Sonnhalde
1046. Academy of Natural Sciences: Herpetology

1047. Kraberg, Alexandra C; Asmus, Ragnhild; van Beusekom, Justus (2012): Phytoplankton abundance in the wadden sea off List, Sylt, North Sea in 2008. Alfred Wegener Institute for Polar and Marine Research - Wadden Sea Station Sylt, doi:10.1594/PANGAEA.783265
1048. University of Gdańsk, Dept. of Plant Taxonomy and Nature Conservation: Orchid Herbarium Collection
1049. Willing, E. (Ed.) 1978 - (continuously updated): Herbarium collection of Eckhard Willing at the Herbarium Berolinense (B).
1050. Schiebel, R et al. (2004): Coccolith counts of multinet M32/5_MSN974. doi:10.1594/PANGAEA.128638, In Supplement to: Schiebel, Ralf; Zeltner, A; Treppke, Ute F; Waniek, Joanna J; Bollmann, Jörg; Rixen, Tim; Hemleben, Christoph (2004): Distribution of diatoms, coccolithophores and planktic foraminifers along a trophic gradient during SW monsoon in the Arabian Sea. Marine Micropaleontology, 51(3-4), 345-371, doi:10.1016/j.marmicro.2004.02.001
1051. GEO-Tag der Artenvielfalt: Dierloch, nördlicher Mooswald (Freiburg-Hochdorf)
1052. GEO-Tag der Artenvielfalt: Weidewirtschaft
1053. GEO-Tag der Artenvielfalt: Bäche, Quellen und Teiche im FFH-Gebiet Mühlhauser Halde
1054. Chaisson, William P; Keigwin, Lloyd D; Rio, Domenico; Acton, Gary D; Shipboard Scientific Party (2005): Range table from planktonic foraminifers in ODP Hole 172-1064A. doi:10.1594/PANGAEA.315518
1055. GEO-Tag der Artenvielfalt: Unser kleines Rasenstück/ Dürer-Gymnasium Nürnberg
1056. Senckenberg: Collection Messelpaläobotanik SMB
1057. IRD - Institute of Research for Development: Herbier de la Guyane
1058. GEO-Tag der Artenvielfalt: Höhle am Neuweg / Sächsische Schweiz
1059. GBIF-Sweden: Lund Museum of Zoology (MZLU)
1060. GEO-Tag der Artenvielfalt: Schulhof A.-Lindgren-Schule (Elmshorn)
1061. UK National Biodiversity Network: Central Scotland Forest Trust - South Lanarkshire peatland records 2013
1062. GEO-Tag der Artenvielfalt: Schulgarten der Volksschule
1063. GBIF-Spain: Herbario de la Universidad de Granada: GDA
1064. Senckenberg: Collection Mikropaläobotanik SMB
1065. Florida Museum of Natural History: invertebratezoology
1066. Below, R (1984): (Figure 1) Distribution of dinoflagellate cysts in the mid-Cretaceous of DSDP Hole 79-545. doi:10.1594/PANGAEA.809491, In Supplement to: Below, Raimond (1984): Aptian to Cenomanian dinoflagellate cysts from the Mazagan Plateau, Northwest Africa (Sites 545 and 547, Deep Sea Drilling Project Leg 79). In: Hinz, K; Winterer, EL; et al. (eds.), Initial Reports of the Deep Sea Drilling Project, Washington (U.S. Govt. Printing Office), 79, 621-649, doi:10.2973/dsdp.proc.79.123.1984
1067. UK National Biodiversity Network: Joint Nature Conservation Committee - Vegetation surveys of coastal shingle in Great Britain

1068. GEO-Tag der Artenvielfalt: VFD-RP: Eifel: Orchideenweide Ankly
1069. Chaisson, William P; Keigwin, Lloyd D; Rio, Domenico; Acton, Gary D; Shipboard Scientific Party (2005): Range table from planktonic foraminifers in ODP Hole 172-1063A. doi:10.1594/PANGAEA.315517
1070. UK National Biodiversity Network: Hertfordshire Biological Records Centre - Roadside Verge Survey 1986 (Herts data from Cambs WT survey)
1071. Cleef. A.M. 1981. The vegetation of the paramos of the Colombian Cordillera Oriental. PhD Thesis. State University of Utrecht. 320pp. Also published as Dissertationes Botanicae, Baud 61, J. Cramer, Vadyz and "The Cuaternary of Colombia", Vol.9., n/a
1072. Real Jardín Botánico (CSIC): Real Jardin Botanico (Madrid), Vascular Plant Herbarium (MA)
1073. Institute of Botany, University of Hohenheim: Visual Plants (144.41.33.158) - Botanical garden, University of Hohenheim, Germany
1074. GEO-Tag der Artenvielfalt: NABU-Auerochsenweide
1075. GBIF-Sweden: SBT-Living
1076. Armonies, Werner (2010): Macrobenthos in surface sediments off Sylt collected during Heincke cruise HE218. Alfred Wegener Institute for Polar and Marine Research - Wadden Sea Station Sylt, doi:10.1594/PANGAEA.745716
1077. Plant Breeding and Acclimatization Institute (IHAR) - National Research Institute: Polish seed gene bank – historical passport data of accessions
1078. National Biodiversity Data Centre: Moths Ireland
1079. ACOI - Coimbra Collection of Algae - University of Coimbra: Coimbra Collection of Algae
1080. UK National Biodiversity Network: Biodiversity Information Service for Powys and Brecon Beacons National Park - Distribution of <i>Impatiens glandulifera</i> Royle along the river Irfon during June 2010
1081. UK National Biodiversity Network: John Muir Trust - Knoydart Bryophytes, 1990
1082. GBIF-Sweden: Herbarium of Oskarshamn (OHN)
1083. GEO-Tag der Artenvielfalt: Birdrace_Schräge_Vögel-Herford
1084. Bye Boettler, R. y M. Mendoza Cruz. 1998. Biodiversidad de Datura (Solanaceae) en México. Universidad Nacional Autónoma de México. Instituto de Biología. Bases de datos SNIB2010-CONABIO proyecto No.P088. México, D.F.
1085. Fernández Nava, R., Reyes Toledo, B. y M. Casales Gómez. 2007. Computarización del Herbario ENCB, IPN. Fase IV. Base de datos de la familia Pinaceae y de distintas familias de la clase Magnoliopsida depositadas en el Herbario de la Escuela Nacional de Ciencias Biológicas, IPN. Instituto Politécnico Nacional. Escuela Nacional de Ciencias Biológicas. Bases de datos SNIB2010-CONABIO proyectos No. BC007. México, D.F.
1086. Ortiz-Pulido, R. 2006. Estudio de la avifauna y de las interacciones ave-planta en la Reserva de la Biosfera de la Barranca de Metztitlán Hidalgo, México. Universidad Autónoma del Estado de Hidalgo. Bases de datos SNIB2010-CONABIO proyecto No. AS010. México D. F.

1087. GEO-Tag der Artenvielfalt: Naturschutzgebiet Lippeaue (Marl) - Pfadis in Sickingmühle
1088. California State University, Chico Herbarium
1089. GEO-Tag der Artenvielfalt: Von Elf bis Elf" Der Botanische Garten Wuppertal
1090. GEO-Tag der Artenvielfalt: Schwanseepark (87645 Schwangau)
1091. Chavan, VIshwas and C. T. Achuthankutty (editors), IndOBIS Catalogue of Life, Available at http://www.indobis.org/, Retrived day, date, year
1092. To be done
1093. Toledo Manzur, V. M. 2005. Potencial económico de la flora útil de los cafetales de la Sierra Norte de Puebla. Universidad Nacional Autónoma de México. Centro de Investigaciones en Ecosistemas. Bases de datos SNIB2010-CONABIO proyecto No. AE019. México, D.F.
1094. GEO-Tag der Artenvielfalt: Varnhalt
1095. National Museum of Nature and Science, Japan: Kochi Prefectural Makino Botanical Garden
1096. GEO-Tag der Artenvielfalt: Pflanzen und Tiere im Burgwald
1097. Basov, IA; Krasheninnikov, VA (1983): (Figure 7) Distribution of benthic foraminifers in the Oligocene to upper Miocene DSDP of Hole 71-513A. doi:10.1594/PANGAEA.232394, In Supplement to: Basov, Ivan A; Krasheninnikov, Valery A (1983): Benthic foraminifers in Mesozoic and Cenozoic sediments of the southwestern Atlantic as an indicator of paleoenvironment, Deep Sea Drilling Project Leg 71. In: Ludwig, WF; Krasheninnikov, VA; et al. (eds.), Initial Reports of the Deep Sea Drilling Project, Washington (U.S. Government Print Office), 394, 739-787, doi:10.2973/dsdp.proc.71.128.1983
1098. Museum für Naturkunde Berlin: MfN - Trace fossils
1099. Chávez Rendón, C. 2006. Actualización e incremento del banco de datos de la colección de herbario del Jardín Etnobotánico de Oaxaca. Centro Cultural Santo Domingo. Bases de datos SNIB2010-CONABIO proyecto No. BC003. México, D.F.
1100. GEO-Tag der Artenvielfalt: Kaisertal
1101. UK National Biodiversity Network: Suffolk Biological Records Centre - Suffolk Biological Records Centre (SBRC) dataset
1102. UK National Biodiversity Network: Countryside Council for Wales - Bullhead Survey Data in Wales
1103. GEO-Tag der Artenvielfalt: NSG Karwendel
1104. National Museum of Nature and Science, Japan: Seaweed (Plantae) Collection of the Seto Marine Biological Laboratory, Kyoto University
1105. NSW Department of Environment, Climate Change, and Water representing the State of New South Wales: OEH Atlas of NSW Wildlife
1106. Botanischer Garten und Botanisches Museum Berlin-Dahlem, Epiphytic Lichens of G. Lettau at the Botanical Museum Berlin-Dahlem
1107. Mammal Research Institute, Polish Academy of Sciences: Mammal Collection

1108. UK National Biodiversity Network: Biological Records Centre - Derek Lott Coleoptera Dataset
1109. GEO-Tag der Artenvielfalt: NABU Naturschutzhof Netttetal (Sassenfeld) e.V.
1110. GEO-Tag der Artenvielfalt: Biotope im Schulumfeld
1111. Department of Organisms and Systems Biology. University of Oviedo: Universidad de Oviedo. Departamento de Biología de Organismos y Sistemas: FCO-Briof
1112. Galkin, SV et al. (2010): (Table 2c) Percentages of energy flow of macrobenthos species in samples from Cruise AMK54 stations in the Novaya Zemlya Trough. doi:10.1594/PANGAEA.769941, In Supplement to: Galkin, Sergey V; Savilova, Tatyana A; Moskalev, Lev I; Kucheruk, Nikita V (2010): Macrobenthos of the Novaya Zemlya Trough. Translated from Okeanologiya, 2010, 50(6), 982-993, Oceanology, 50(6), 933-944, doi:10.1134/S0001437010060135
1113. UK National Biodiversity Network: Sussex Biodiversity Record Centre - Sussex Moth data for 2011 and 2012 DEFRA FUNDED
1114. GEO-Tag der Artenvielfalt: Willersalpe
1115. Korea Institutie of Water and Enviroment: Alga (KIWE-AG)
1116. GEO-Tag der Artenvielfalt: Tongrube bei Hettstedt
1117. GEO-Tag der Artenvielfalt: "Schlechteberg" Ebersbach/Sa.
1118. Data provided by the participants of the Consortium of California Herbaria (ucjeps.berkeley.edu/consortium/)
1119. GEO-Tag der Artenvielfalt: FND "Weißer Berg" Leißling
1120. Field Museum: Field Museum of Natural History (Botany) Fungi Collection
1121. Wrocław University, Fac. Natural Sciences: Flora of Słowiński National Park, Poland
1122. University of Amsterdam / IBED: University of Amsterdam (NL) - Páramo vegetation research, Talamanca Cordillera, Costa Rica
1123. GEO-Tag der Artenvielfalt: Insektenvielfalt Ahe/Weichelsee
1124. GEO-Tag der Artenvielfalt: Artenvielfalt im Umfeld der Burgwegschule
1125. Martínez, M. 1999. Flora y vegetación de la Sierra de San Carlos en el municipio de San Nicolás, Tamaulipas. Universidad Autónoma de Querétaro. Facultad de Ciencias Naturales. Bases de datos SNIB2010-CONABIO proyecto No. L029. México, D.F.
1126. GEO-Tag der Artenvielfalt: Silbertor + Wasserbachtal (Rutesheim / Renningen)
1127. GEO-Tag der Artenvielfalt: Feriendorf des Kreises Groß-Gerau Ober-Seemen/Gedern
1128. Renker, C. (Ed.) 2010+ (continuously updated): Digitised specimen data at Naturhistorisches Museum Mainz / Landessammlung für Naturkunde Rheinland-Pfalz (MNHM).
1129. GEO-Tag der Artenvielfalt: Halbwilde Weidehaltung zwischen Kamp-Bornhofen und Kestert sowie Umland
1130. Proto Decima, Franka; Bolli, Hans M; Ryan, William B F (2005): Benthic foraminifera and nannofossil abundance of Hole 40-360. doi:10.1594/PANGAEA.250840

1131. GEO-Tag der Artenvielfalt: Artenvielfalt der Nordsee - Helgoland
1132. GEO-Tag der Artenvielfalt: Selz-Renaturierung (Hahnheim/Sörgenloch)
1133. GEO-Tag der Artenvielfalt: Streuobstwiese
1134. GEO-Tag der Artenvielfalt: Waldsee
1135. GEO-Tag der Artenvielfalt: Vogelwelt im Europa-Rosarium
1136. GEO-Tag der Artenvielfalt: 10. GEO - Tag der Artenvielfalt 2008 - LSG "Pfarrhübel" Chemnitz
1137. Cleef A. 1977, 1983, 1984, 1989. Field Data.
