# Supplementary material for: Diversification of flowering plants in space and time
Source: Nat Commun. 2023 Nov 22;14:7609. doi: 10.1038/s41467-023-43396-8 (PMC10665465; doi:10.1038/s41467-023-43396-8)
Supplement: Supplementary file 4 — Supplementary Data 2 [file 41467_2023_43396_MOESM4_ESM.pdf]

Supplementary Data 2. List of fossils used in the dating analyses and the corresponding references.

1. Acrogymnospermae (crown min age 290Ma)- Mapes, G. & Rothwell, G. W. Permineralized ovulate cones of *Lebachia* from late Palaeozoic limestones of Kansas. Palaeontology 27, 69–94 (1984).
Mapes, G. & Rothwell G. W. Structure and relationship of primitive conifers. N. Jb. Geol. Paläont. 183, 269–287 (1991).

2. Magnoliales (stem min age 108.8Ma)- Crane, P. R., Friis, E. M. & Pedersen, K. R. Palaeobotanical evidence on the early radiation of magnoliid angiosperms. Plant Syst. Evol. 8, 51–72 (1994).

3. Pandanales (crown min age 65)- Jarzen, D. M. The terrestrial palynoflora from the Cretaceous-Tertiary transition, Alabama, U.S.A. Pollen and Spores 20, 535–553 (1978).
Muller, J. Fossil pollen records of extant angiosperms. Bot. Rev. 47, 1–142 (1981).

4. Arecales (crown min age 65) - Pan, A. D., Jacobs, B. F., Dransfield, J. & Baker, W. J. The fossil history of palms (Arecaceae) in Africa and new records from the Late Oligocene (28‑27 Mya) of north ‑ western Ethiopia. Bot. J. Linn. Soc. 151, 69‑81 (2006).

5. Eudicotyledonae (crown min age 125Ma)- Hughes, N. F. & McDougall, A. B. Barremian-Aptian angiospermid pollen records from southern England. Rev. Palaeobot. Palynol. 65, 145–151 (1990).
Doyle, J. A. Revised palynological correlations of the lower Potomac group (USA) and the cocobeach sequence of Gabon (Barremian-Aptian). Cretaceous Res. 13, 337–349 (1992).
Brenner G. J. in Flowering Plant Origin, Evolution, and Phylogeny (Taylor, D. W. & Hickey, L. J.) 91–115 (Chapman & Hall, New York, 1996)
Friis, E. M., Pedersen, K. R. & Crane, P. R. Cretaceous angiosperm flowers: Innovation and evolution in plant reproduction. Palaeogeogr., Palaeoclimatol., Palaeoecol. 232, 251–293 (2006).

6. Proteales (crown min age 108.8Ma)- Crane, P. R. & Herendeen, P. S. Cretaceous floras containing angiosperm flowers and fruits from eastern North America. Rev. Palaeobot. Palynol. 90, 319–337 (1996).

7. Buxales (stem min age 112Ma)- Anderson, C. L., Bremer, K. & Friis, E. M. Dating phylogenetically basal eudicots using rbcL sequences and multiple fossil reference points. Am. J. Bot. 92, 1737–1748 (2005).

8. Gunnerales (crown min age 88.2Ma)- Muller, J. Fossil pollen records of extant angiosperms. Bot. Rev. 47, 1–142 (1981).

9. Caryophyllales (crown min age 83.5Ma) - Collinson, M. E., Boulter, M. C. & Holmes, P. L. in The Fossil Record 2 (Benton, M. J.) 809–841 (Chapman and Hall, London, 1993).

10. Cornales (crown min age 86.3Ma) - Takahashi M, Crane PR, Manchester SR. 2002. Hironoia fusiformis gen. et sp. nov.; a cornalean fruit from the Kamikitaba locality (Upper Cretaceous, Lower Coniacian) in northeastern Japan. Journal of Plant Research 115: 463–473.

11. Ericales (crown min age 91.2Ma) - Nixon, K. C. & Crepet, W. L. Late Cretaceous fossil flowers of ericalean affinity. Am. J. Bot. 80, 616–623 (1993).

12. Saxifragales (crown min age 89.3Ma) - Hermsen, E. J. et al. Divisestylus gen. nov. (aff. Iteaceae), a fossil saxifrage from the Late Cretaceous of New Jersey, USA. Am. J. Bot. 90, 1373–1388 (2003).

13. Myrtales (crown min age 88.2Ma) - Takahashi, M., Crane, P. R. & Ando, H. Esgueiria futabensis sp. nov., a new angiosperm flower from the Upper Cretaceous (lower Coniacian) of northeastern Honshu, Japan. Paleontol. Res. 3, 81–87 (1999).

14. Sapindales (crown min age 65Ma) - Knobloch, E. D. & Mai, D. H. Monograph of the fruits and seeds in the Cretaceous of Central Europe. Rozpravy Ústreõdního Ústavu Geologického 47, 1–219 (1986).

15. Fabales (crown min age 59.9Ma) - Herendeen, P. S. & Crane, P. R. in Advances in Legume systematics, part 4. The fossil record. (Herendeen, P. S. & Dilcher, D. L.) 57–68 (Royal Botanic Gardens, Kew, 1992).

16. Fagales (stem min age 96Ma) - Friis, E. M., Pedersen, K. R. & Schönenberger, J. Normapolles plants: a prominent component of the Cretaceous rosid diversification. Plant Syst. Evol. 260, 107–140 (2006).

17. Austrobaileyales (crown min age 113Ma) - Upchurch GR. 1984. Cuticular Anatomy of Angiosperm Leaves from the Lower Cretaceous Potomac Group. I. Zone I Leaves. American Journal of Botany 71: 192–202.

18. Cabombaceae (Nymphaeales) (stem min age 100.5Ma) - Taylor DW, Brenner GJ, Basha SH. 2008. Scutifolium jordanicum gen. et sp. nov. (Cabombaceae), an aquatic fossil plant from the Lower Cretaceous of Jordan, and the relationships of related leaf fossils to living genera. American Journal of Botany 95: 340–352.

19. Ceratophyllum (Ceratophyllaceae, Ceratophyllales) (stem min age 127.2Ma) - Gomez B, Daviero-Gomez V, Coiffard C, Martín-Closas C, Dilcher DL. 2015. Montsechia, an ancient aquatic angiosperm. Proceedings of the National Academy of Sciences 112: 10985–10988.

20. Hedyosmum (Chloranthaceae, Chloranthales) (stem min age 123Ma) - Friis EM, Pedersen KR, Crane PR. 1994. Angiosperm floral structures from the Early Cretaceous of Portugal (PK Endress and EM Friis, Eds.). Pl. Syst. Evol. Suppl. 8: 31–49.

21. Winteraceae (Canellales) (stem min age 125Ma) - Doyle JA, Hotton CL, Ward J V. 1990. Early Cretaceous tetrads, zonasulculate pollen, and Winteraceae. I. Taxonomy, morphology, and ultrastructure. American Journal of Botany 77: 1544–1557.

22. Calycanthoideae (Calycanthaceae, Laurales) (crown min age 86.3Ma) - Crepet WL, Nixon KC, Gandolfo MA. 2005. An extinct calycanthoid taxon, Jerseyanthus calycanthoides , from the Late Cretaceous of New Jersey. American Journal of Botany 92: 1475–1485.

23. Hernandiaceae (Laurales) (crown min age 41.2Ma) - Manchester SR, O'Leary EL. 2010. Phylogenetic distribution and identification of fin-winged fruits. Botanical Review 76: 1–82.

24. Magnoliaceae (Magnoliales) (stem min age 96.5Ma) - Dilcher DL, Crane PR. 1984. Archaenthus: An Early Angiosperm From the Cenomanian of the Western Interior of North America. Annals of the Missouri Botanical Garden 71: 351–383.

25. Hydrocharitaceae (Alismatales) (crown min age 55.9Ma) - Bone D. 1986. The stratigraphy of the Reading Beds (Palaeocene), at Felpham, West Sussex. Tertiary Research 8: 17–32.

26. Posidoniaceae (Alismatales) (stem min age 66Ma) - van der Ham RWJM, van Konijnenburg-van Cittert JHA, Indeherberge L. 2007. Seagrass foliage from the Maastrichtian type area (Maastrichtian, Danian, NE Belgium, SE Netherlands). Review of Palaeobotany and Palynology 144: 301–321.

27. Asteliaceae (Asparagales) (crown min age 23.2Ma) - Maciunas E, Conran JG, Bannister JM, Paull R, Lee DE. 2011. Miocene Astelia (Asparagales: Asteliaceae) macrofossils from southern New Zealand. Australian Systematic Botany 24: 19–31.

28. Alstroemeriaceae (Liliales) (crown min age 23.2Ma) - Conran JG, Bannister JM, Mildenhall DC, Lee DE, Chacón J, Renner SS. 2014. Leaf fossils of Luzuriaga and a monocot flower with in situ pollen of Liliacidites contortus Mildenh. & Bannister sp. nov. (Alstroemeriaceae) from the Early Miocene. American Journal of Botany 101: 141–155.

29. Ripogonaceae (Liliales) (stem min age 51Ma) - Conran JG, Carpenter RJ, Jordan GJ. 2009. Early Eocene Ripogonum (Liliales: Ripogonaceae) leaf macrofossils from southern Australia. Australian Systematic Botany 22: 219–228.

30. Cyclanthaceae (Pandanales) (crown min age 47Ma) - Smith SY, Collinson ME, Rudall PJ. 2008. Fossil Cyclanthus (Cyclanthaceae, Pandanales) from the eocene of Germany and England. American Journal of Botany 95: 688–699.

31. Cyperaceae+Juncaceae (stem min age 44.3Ma) - Erwin DM, Stockey RA. 1992. Vegetative body of a permineralized monocotyledon from the Middle Eocene Princeton Chert of British Columbia. Courier Forschungsinstitut Senckenberg 147: 309–327.

32. Bromeliaceae (Poales) (crown min age 41.2Ma) - Graham A. 1985. Studies in Neotropical Paleobotany. IV. The Eocene Communities of Panama. Annals of the Missouri Botanical Garden 72: 504–534.

33. Cyperaceae (Poales) (crown min age 47Ma) - Smith SY, Collinson ME, Simpson DA, Rudall PJ, Marone F, Stampanoni M. 2009. Elucidating the affinities and habitat of ancient, widespread Cyperaceae: Volkeria messelensis gen. et sp. nov., a fossil mapanioid sedge from the Eocene of Europe. American Journal of Botany 96: 1506–1518.

34. Restionaceae (Poales) (stem min age 27.7Ma) - Dettmann ME, Clifford HT. 2000. Monocotyledon fruits and seeds, and an associated palynoflora from Eocene–

Oligocene sediments of coastal central Queensland, Australia. Review of Palaeobotany and Palynology 110: 141–173.

35. **Typhaceae** (Poales) (crown min age 51.66Ma) - Grande L. 1984. Paleontology of the Green River Formation, with a review of the fish fauna, second edition. Geological Survey of Wyoming Bulletin 63: 1–333.

36. **Zingiberaceae** (Zingiberales) (stem min age 66Ma) - Knowlton F. 1917. Fossil floras of the Vermejo and Raton formations of Colorado and New Mexico. U.S. Geological Survey Professional Paper 101: 223–435.

37. **Buxaceae** (Buxales) (stem min age 100.5Ma) - Drinnan AN, Crane PR, Friis EM, Pedersen KR. 1991. Angiosperm flowers and tricolpate pollen of buxaceous affinity from the Potomac Group (Mid-Cretaceous) of Eastern North America. American Journal of Botany 78: 153–176.

38. **Platanaceae** (Proteales) (stem min age 107.7Ma) - Crane PR, Pedersen KR, Friis EM, Drinnan AN. 1993. Early Cretaceous (early to middle Albian) platanoid inflorescences associated with Sapindopsis leaves from the Potomac Group of Eastern North America. Systematic Botany 18: 328–344.

39. **Proteaceae** (Proteales) (crown min age 72.1Ma) - Carpenter RJ, Macphail MK, Jordan GJ, Hill RS. 2015. Fossil evidence for open, proteaceae-dominated heathlands and fire in the late cretaceous of Australia. American Journal of Botany 102: 2092–2107.

40. **Menispermaceae** (Ranunculales) (stem min age 89.8Ma) - Knobloch E, Mai DH. 1984. Neue Gattungen nach Früchten und Samen aus dem Cenoman bis Maastricht (Kreide) von Mitteleuropa. Feddes Repertorium 95: 3–41.

41. **Dilleniaceae** (Dilleniales) (crown min age 47.8Ma) - Reid EM, Chandler MEJ. 1933. The London Clay flora. British Museum (Natural History), London, UK.

42. **Haloragaceae** (Saxifragales) (stem min age 72.1Ma) - Hernandez-Castillo GR, Cevallos-Ferriz SRS. 1999. Reproductive and vegetative organs with affinities to Haloragaceae from the Upper Cretaceous Huepac Chert Locality of Sonora, Mexico. American Journal of Botany 86: 1717.

43. **Hamamelidaceae** (Saxifragales) (crown min age 83.6Ma) - Magallón-Puebla S, Herendeen PS, Endress PK. 1996. Allonia decandra: Floral remains of the tribe

Hamamelideae (Hamamelidaceae) from Campanian strata of southeastern USA. Plant Systematics and Evolution 202: 177–198.

44. **Iteaceae** (Saxifragales) (crown min age 48Ma) - Wolfe JA, Wehr WC. 1987. Middle Eocene dicotyledonous plants from Republic, northeastern Washington. United States Geological Survey Bulletin 1597: 1–25.

45. **Staphyleaceae** (Crossosomatales) (crown min age 56Ma) - Knowlton F. 1917. Fossil floras of the Vermejo and Raton formations of Colorado and New Mexico. U.S. Geological Survey Professional Paper 101: 223–435.

46. **Francoaceae** (Geraniales) (crown min age 15.97Ma) - Palazzesi L, Gottschling M, Barreda V, Weigend M. 2012. First Miocene fossils of Vivianiaceae shed new light on phylogeny, divergence times, and historical biogeography of Geraniales. Biological Journal of the Linnean Society 107: 67–85.

47. **Combretaceae** (Myrtales) (stem min age 86.3Ma) - Takahashi M, Crane PR, Ando H. 1999. Esgueiria futabensis sp. nov., a new angiosperm flower from the Upper Cretaceous (Lower Coniacian) of northeastern Honshu, Japan. Paleontological Research 3: 81–87.

48. **Lythraceae** (Myrtales) (crown min age 72.1Ma) - Estrada-Ruiz E, Calvillo-Canadell L, Cevallos-Ferriz SRS. 2009. Upper Cretaceous aquatic plants from Northern Mexico. Aquatic Botany 90: 282–288.

49. **Melastomataceae** (Myrtales) (stem min age 56Ma) - Brown RW 1962. Paleocene flora of the Rocky Mountains and Great Plains. Geological Survey Professional Paper 375: 1-119.

50. **Vitaceae** (Vitales) (crown min age 61.6Ma) - Manchester SR, Kapgate DK, Wen J. 2013. Oldest fruits of the grape family (Vitaceae) from the Late Cretaceous Deccan Cherts of India. American Journal of Botany 100: 1849–1859.

51. **Akaniaceae** (Brassicales) (crown min age 61.6Ma) - Iglesias A, Wilf P, Johnson KR, Zamuner AB, Cúneo NR, Matheos SD, Singer BS. 2007. A Paleocene lowland macroflora from Patagonia reveals significantly greater richness than North American analogs. Geology 35: 947–950.

52. Tapisciaceae (Huerteales) (crown min age 41.2Ma) - Manchester SR. 1988. Fruits and seeds of Tapiscia (Staphylaceae) from the Middle Eocene of Oregon U.S.A. Tertiary Research 9: 59-66.

53. Dipterocarpaceae (Malvales) (crown min age 56Ma) - Mandal J, Rao MR. 2001. Taxonomic revision of tricolpate pollen from Indian Tertiary. Palaeobotanist 50: 341–368.

54. Malvaceae (Malvales) (stem min age 72.1Ma) - Estrada-Ruiz E, Martínez-Cabrera HI, Cevallos-Ferriz SRS. 2007. Fossil woods from the late Campanian-early Maastrichtian Olmos Formation, Coahuila, Mexico. Review of Palaeobotany and Palynology 145: 123–133.

55. Meliaceae (Sapindales) (crown min age 47.8Ma) - Reid EM, Chandler MEJ. 1933. The London Clay flora. British Museum (Natural History), London, UK.

56. Simaroubaceae (Sapindales) (crown min age 41.2Ma) - Collinson ME, Manchester SR, Wilde V. 2012. Fossil fruits and seeds of the Middle Eocene Messel biota, Germany. Abh. Senckenberg Ges. Naturforsch. 570: 1–251.

57. Celastraceae (Celastrales) (crown min age 37.8Ma) - Hollick A. 1936. The Tertiary floras of Alaska. U.S. Geological Survey Professional Paper 182: 1–185.

58. Celastraceae (Celastrales) (stem min age 46.3Ma) - Mehrotra RC, Prakash U, Bande MB. 1984. Fossil woods of Lophopetalum and Artocarpus from the Deccan Intertrappean Beds of Mandla district, Madhya Pradesh, India. Palaeobotanist 32: 310–320.

59. Anisophylleaceae (Cucurbitales) (crown min age 11.63Ma) - Anderson JAR, Muller J. 1975. Palynological study of a holocene peat and a miocene coal deposit from NW Borneo. Review of Palaeobotany and Palynology 19: 291–351.

60. Cucurbitaceae (Cucurbitales) (stem min age 47.8Ma) - Chandler MEJ. 1961. The Lower Tertiary floras of southern England. I. Paleocene Floras. London Clay Flora (Supplement). Text and Atlas. London: British Museum (Natural History).

61. Polygalaceae (Fabales) (crown min age 56Ma) - Pigg KB, DeVore ML, Wojciechowski MF. 2008. Paleosecuridaca curtisii gen. et sp. nov., Securidaca-Like Samaras (Polygalaceae) from the Late Paleocene of North Dakota and Their Significance to

the Divergence of Families within the Fabales. International Journal of Plant Sciences 169: 1304–1313.

62. **Surianaceae** (Fabales) (crown min age 48.5Ma) - Kruse H. 1954. Some Eocene dicotyledonous Woods from Eden Valley, Wyoming. Ohio Journal of Science 54: 243–268.

63. **Fagaceae** (Fagales) (crown min age 37.8Ma) - Crepet WL, Daghlian CP. 1980. Castaneoid inflorescences from the Middle Eocene of Tennessee and the diagnostic value of pollen (at the subfamily level) in Fagaceae. American Journal of Botany 67: 739–757.

64. **Juglandaceae** (Fagales) (crown min age 64.4Ma) - Manchester SR, Dilcher DL. 1982. Pterocaryoid fruits (Juglandaceae) in the Paleogene of North America and their evolutionary and biogeographic significance. American Journal of Botany 69: 275–286.

65. **Nothofagaceae** (Fagales) (stem min age 83.5Ma) - Dettmann ME, Playford G. 1968. Taxonomy of some Cretaceous spores and pollen grains from Eastern Australia. Proceedings of the Royal Society of Victoria 81: 69–93.

66. **Chrysobalanaceae** (Malpighiales) (crown min age 48.5Ma) - Brown RW. 1929. Additions to the flora of the Green River formation. U.S. Geological Survey Professional Paper 154: 279–292.

67. **Clusiaceae** (Malpighiales) (stem min age 86.3Ma) - Crepet WL, Nixon KC. 1998. Fossil Clusiaceae from the Late Cretaceous (Turonian) of New Jersey and implications regarding the history of bee pollination. American Journal of Botany 85: 1122–1133.

68. **Ctenolophonaceae** (Malpighiales) (stem min age 66Ma) - van Hoeken-Klinkenberg P. 1964. A palynological investigation of some Upper Cretaceous sediments in Nigeria. Pollen et Spores 6: 209–231.

69. **Humiriaceae** (Malpighiales) (crown min age 33.9Ma) - Herrera F, Manchester SR, Vélez-Juarbe J, Jaramillo C. 2014. Phytogeographic history of the Humiriaceae (part 2). International Journal of Plant Sciences 175: 828–840.

70. **Rhizophoraceae** (Malpighiales) (crown min age 33.9Ma) - Germeraad JH, Hopping CA, Muller J. 1968. Palynology of Tertiary sediments from tropical areas. Review of Palaeobotany and Palynology 6: 189–348.

71. **Cunoniaceae** (Oxalidales) (crown min age 56Ma) - Hill RS. 1991. Leaves of Eucryphia (Eucryphiaceae) from Tertiary Sediments in South-eastern Australia. Australian Systematic Botany 4: 481-497.

72. **Cunoniaceae** (Oxalidales) (stem min age 80.7Ma) - Schönenberger J, Friis EM, Matthews ML, Endress PK 2001 Cunoniaceae in the Cretaceous of Europe: Evidence from fossil flowers. Annals of Botany 88: 423-437.

73. **Elaeocarpaceae** (Oxalidales) (crown min age 61.6Ma) - Manchester SR, Kvacek Z. 2009. Fruits of Sloanea (Elaeocarpaceae) in the Paleogene of North America and Greenland. International Journal of Plant Sciences 170: 941–950.

74. **Moraceae** (Rosales) (crown min age 47.8Ma) - Chandler MEJ. 1963. The Lower Tertiary floras of Southern England. III. Flora of the Bournemouth Beds; the Boscombe, and the Highcliff Sands. London: British Museum (Natural History).

75. **Rhamnaceae** (Rosales) (stem min age 72.1Ma) - Calvillo-Canadell L, Cevallos-Ferriz SRS. 2007. Reproductive structures of Rhamnaceae from the Cerro del Pueblo (Late Cretaceous, Coahuila) and Coatzingo (Oligocene, Puebla) Formations, Mexico. American Journal of Botany 94: 1658–1669.

76. **Polygonaceae** (Caryophyllales) (stem min age 66Ma) - Manchester SR, O'Leary EL. 2010. Phylogenetic distribution and identification of fin-winged fruits. Botanical Review 76: 1–82.

77. **Olacaceae** (Santalales) (stem min age 66Ma) - Chmura CA. 1973. Upper Cretaceous (Campanian-Maastrichtian) angiosperm pollen from the western San Joaquin Valley, California, U.S.A. Palaeontographica Abteilung B 141: 89–171.

78. **Schoepfiaceae** (Santalales) (crown min age 48Ma) - Wolfe JA, Wehr WC. 1987. Middle Eocene dicotyledonous plants from Republic, northeastern Washington. United States Geological Survey Bulletin 1597: 1–25.

79. **Hydrangeaceae** (Cornales) (crown min age 56Ma) - Brown RW 1962. Paleocene flora of the Rocky Mountains and Great Plains. Geological Survey Professional Paper 375: 1-119.

80. Actinidiaceae (Ericales) (crown min age 83.6Ma) - Keller JA, Herendeen PS, Crane PR. 1996. Fossil flowers and fruits of the Actinidiaceae from the Campanian (Late Cretaceous) of Georgia. American Journal of Botany 83: 528–541.

81. Vaccinioideae (Ericaceae, Ericales) (crown min age 66Ma) - Knobloch E, Mai DH. 1986. Monographie der Früchte und Samen in der Kreide von Mitteleuropa. Rozpravy ústredního ústavu geologichénho. Praha 47: 1-219.

82. Pentaphylacaceae (Ericales) (stem min age 66Ma) - Knobloch E, Mai DH. 1986. Monographie der Früchte und Samen in der Kreide von Mitteleuropa. Rozpravy ústredního ústavu geologichénho. Praha 47: 1-219.

83. Polemoniaceae (Ericales) (crown min age 48.5Ma) - Lott TA, Manchester SR, Dilcher DL. 1998. A unique and complete polemoniaceous plant from the middle Eocene of Utah, USA. Review of Palaeobotany and Palynology 104: 39–49.

84. Roridulaceae (Ericales) (crown min age 33.9Ma) - Sadowski E-M, Seyfullah LJ, Sadowski F, Fleischmann A, Behling H, Schmidt AR. 2015. Carnivorous leaves from Baltic amber. Proceedings of the National Academy of Sciences 112: 190–195.

85. Sapotaceae (Ericales) (crown min age56Ma) - Mehrotra, R. C. 2000. Study of plant megafossils from the Tura Formation of Nangwalbibra, Garo Hills, Meghalaya, India. The Palaeobotanist 49(2): 255–237.

86. Styracaceae (Ericales) (crown min age 48.5Ma) - MacGinitie 1969. The Eocene Green River flora of northwestern Colorado and northeastern Utah. Univ. Calif. Publ. Geo. Sci. 83: 1-140

87. Symplocaceae (Ericales) (crown min age 47.8Ma) - Tiffney B H. 1999. Fossil fruit and seed flora from the Early Eocene Fisher/Sullivan Site. Pages 139–159 in RE Weems & GJ Grimsley (eds.), Early Eocene vertebrates and plants from the Fisher/Sullivan Site (Nanjemoy Formation) Stafford County, Virginia. Virginia Division of Mineral Resources Publication.

88. Tetrameristaceae (Ericales) (crown min age 23.03Ma) - Van der Hammen T, Wymstra TA. 1964. A palynological study on the tertiary and Upper Cretaceous of British Guiana. Leidse Geologische Mededelingen 30: 183–241.

89. Theaceae (Ericales) (crown min age 37.8Ma) - Grote PJ, Dilcher DL. 1989. Investigations of Angiosperms from the Eocene of North America: A New Genus of Theaceae Based on Fruit and Seed Remains. Botanical Gazette 150: 190-206

90. Aquifoliaceae (Aquifoliales) (stem min age 61.6Ma) - Mai DH. 1970. Subtropische Elemente im europäischen Tertiär I. Die Gattungen Gironniera, Sarcococca, Illicium, Evodia, Ilex, Mastixia, Alangium, Symplocos und Rehderodendron. Paläontologische Abhandlungen Abt. B 3: 441–503, pls. 58–69.

91. Goodeniaceae (Asterales) (crown min age 23.03Ma) - Pocknall DT. 1982. Palynology of late Oligocene Pomahaka Estuarine Bed sediments, Waikoikoi, Southland, New Zealand, New Zealand Journal of Botany, 20:3, 263-287

92. Menyanthaceae (Asterales) (crown min age 5.333Ma) - Lancucka-Srodoniowa M. 1979. Macroscopical plant remains from the freshwater Miocene of the Nowy Sacz Basin (West Carpathians, Poland). Acta Palaeobotanica 20: 3–117.

93. Ehretiaceae (Boraginales) (crown min age 47.8Ma) - Chandler MEJ. 1961. The Lower Tertiary floras of southern England. I. Paleocene Floras. London Clay Flora (Supplement). Text and Atlas. London: British Museum (Natural History).

94. Eucommiaceae (Garryales) (stem min age 47.8Ma) - Brown RW. 1940. New species and changes of name in some American fossil floras. Journal of the Washington Academy of Sciences 30: 344–356.

95. Icacinaceae (Icacinales) (crown min age 47.8Ma) - Del Rio C, Haevermans T, De Franceschi D. 2017. First record of an Icacinaceae Miers fossil flower from le Quesnoy (Ypresian, France) amber. Scientific Reports 7: 1–8.

96. Byblidaceae (Lamiales) (crown min age 41.2Ma) - Conran JG, Christophel DC. 2004. A Fossil Byblidaceae Seed from Eocene South Australia. International Journal of Plant Sciences 165: 691–694.

97. Oleaceae (Lamiales) (crown min age 27.82Ma) - Becker, HF. 1961. Oligocene plants from the Upper Ruby River Basin, South-Western Montana. The Geological Society of America Memoir 82. Waverly Press, New York.

98. Paulowniaceae (Lamiales) (stem min age 11.63Ma) - Butzmann R, Fischer T. 1997. Description of the fossil fruit Paulownia inopinata nov. sp. from the Middle Miocene

of Unterwohlbach (Bavaria) and other possible occurrences of the genus in the Tertiary. Documenta Naturae 115: 1–13.

99. Convolvulaceae (Solanales) (crown min age 47.8Ma) - Salard-Cheboldaeff M. 1975. Quelques grains de pollen peripores Tertiaires du Cameroun. Rev. Micropaleontol. 17: 182–190.

100. Solanaceae (Solanales) (crown min age 52.22Ma) - Wilf P, Carvalho MR, Gandolfo MA, Cúneo NRC. 2017. Eocene lantern fruits from Gondwanan Patagonia and the early origins of Solanaceae. Science 355: 71-75.
