# Supplementary material for: Diversification of flowering plants in space and time
Source: Nat Commun. 2023 Nov 22;14:7609. doi: 10.1038/s41467-023-43396-8 (PMC10665465; doi:10.1038/s41467-023-43396-8)
Supplement: Supplementary file 8 — Reporting Summary [file 41467_2023_43396_MOESM8_ESM.pdf]

nature portfolio

Corresponding author(s): Zhiheng Wang

Last updated by author(s): Oct 20, 2023

Reporting Summary

Nature Portfolio wishes to improve the reproducibility of the work that we publish. This form provides structure for consistency and transparency in reporting. For further information on Nature Portfolio policies, see our Editorial Policies and the Editorial Policy Checklist.

Statistics

For all statistical analyses, confirm that the following items are present in the figure legend, table legend, main text, or Methods section.

n/a	Confirmed	
☐	☒	The exact sample size (*n*) for each experimental group/condition, given as a discrete number and unit of measurement
☒	☐	A statement on whether measurements were taken from distinct samples or whether the same sample was measured repeatedly
☐	☒	The statistical test(s) used AND whether they are one- or two-sided<br>*Only common tests should be described solely by name; describe more complex techniques in the Methods section.*
☐	☒	A description of all covariates tested
☐	☒	A description of any assumptions or corrections, such as tests of normality and adjustment for multiple comparisons
☐	☒	A full description of the statistical parameters including central tendency (e.g. means) or other basic estimates (e.g. regression coefficient) AND variation (e.g. standard deviation) or associated estimates of uncertainty (e.g. confidence intervals)
☐	☒	For null hypothesis testing, the test statistic (e.g. *F*, *t*, *r*) with confidence intervals, effect sizes, degrees of freedom and *P* value noted<br>*Give P values as exact values whenever suitable.*
☐	☒	For Bayesian analysis, information on the choice of priors and Markov chain Monte Carlo settings
☒	☐	For hierarchical and complex designs, identification of the appropriate level for tests and full reporting of outcomes
☒	☐	Estimates of effect sizes (e.g. Cohen's *d*, Pearson's *r*), indicating how they were calculated

Our web collection on statistics for biologists contains articles on many of the points above.

Software and code

Policy information about availability of computer code

Data collection	DNA data was extracted from GenBank using NCBIminer v. 4.0. Other data was directly downloaded from repositories or recorded from the literature.
Data analysis	During the work on this manuscript, we did not develop custom code, novel computation algorithms or mathematical approaches. We used the software packages which are listed here. NCBIminer v4.0, MAFFT v7.4, RAxML v8.0.26, treePL v1.0, BEAST v1.8.0, Tracer v1.6.0, ArcGIS v10, ArcGIS v10.1, MATLAB v2017a, MATLAB v2013b, the MATLAB "fitlm" and "lsqfitgm" functions downloaded from https://www.mbari.org/index-of-downloadable-files, SQL server v2008 R2, BAMM v2.3.0, R package package "BAMMtools" v2.0.6, RevBayes v1.0.10, R package "RPANDA" v1.5, R package "RevGadgets" v1.0.0, R package “multcompView” v0.1-7, R package “rcompanion” v2.3.0, R v3.6.0

For manuscripts utilizing custom algorithms or software that are central to the research but not yet described in published literature, software must be made available to editors and reviewers. We strongly encourage code deposition in a community repository (e.g. GitHub). See the Nature Portfolio guidelines for submitting code & software for further information.

Data

Policy information about availability of data

All manuscripts must include a data availability statement. This statement should provide the following information, where applicable:

- Accession codes, unique identifiers, or web links for publicly available datasets
- A description of any restrictions on data availability
- For clinical datasets or third party data, please ensure that the statement adheres to our policy

All information needed to evaluate the results and conclusions presented in this study is provided in the manuscript and/or supplementary materials. Phylogenies are publicly available at https://en.geodata.pku.edu.cn/index.php?c=content&a=list&catid=200. Distributional data and diversification rates estimates are provided in Supplementary Data 5. Distribution data was obtained from both on-line databases and directly from the literature and the complete list of distributional data sources is provided in Supplementary Data 1. Species distribution data recorded as locality names were searched in the global geographical names service http://www.geonames.org. Global Administrative Areas boundaries were downloaded from http://www.gadm.org and were used as a base to develop the geographical units used in our spatial analyses. The shape file of the geographical units used in the analyses is included in Supplementary Data 5. Family level evolutionary rate estimates are provided in Supplementary Data 3. All sequences used in the phylogenetic analyses are available in GenBank and accession numbers for all sequences used in the analyses are provided in Supplementary Data 4. Sampling fractions used in the BAMM analyses are available in Supplementary Data 4. The information on fossil calibrations used in the analyses along with a complete list of the relevant references is provided in Supplementary Data 2. The taxonomic status and accepted names of species were standardized using the Catalogue of Life (http://www.catalogueoflife.org/col/, accessed: May, 2018), the plant list (TPL; http://www.theplantlist.org/), World Checklist of Selected Plant Families (http://apps.kew.org/wcsp/) and Tropicos (http://www.tropicos.org/). Misspelled taxonomic names were corrected using the Taxonomic Name Resolution Service 4.0 (TNRS, http://tnrs.iplantcollaborative.org/TNRSapp.html, accessed: May, 2018). The final data set was also compared to the Plant of The World on-line (PTW, http://plantsoftheworldonline.org/, accessed: August, 2023). Climate data was downloaded from the WorldClim database (v2.0) and Chelsea (v2.0) https://chelsa-climate.org/ and climatic data used in the analyses are provided in Supplementary Data 5. Photosynthetic pathway data was collected directly from the literature (Smith & Winter 1996, Silvera 2010, Winter et al., 2015 and Sage 2016). Growth form data was obtained from the Plant Trait Database https://www.try-db.org/ and from published databases (Zanne at al. 2014 and Engemann et al. 2016)

Research involving human participants, their data, or biological material

Policy information about studies with human participants or human data. See also policy information about sex, gender (identity/presentation), and sexual orientation and race, ethnicity and racism.

Reporting on sex and gender	Not applicable to this study
Reporting on race, ethnicity, or other socially relevant groupings	Not applicable to this study
Population characteristics	Not applicable to this study
Recruitment	Not applicable to this study
Ethics oversight	Not applicable to this study

Note that full information on the approval of the study protocol must also be provided in the manuscript.

Field-specific reporting

Please select the one below that is the best fit for your research. If you are not sure, read the appropriate sections before making your selection.

☐ Life sciences ☐ Behavioural & social sciences ☒ Ecological, evolutionary & environmental sciences

For a reference copy of the document with all sections, see nature.com/documents/nr-reporting-summary-flat.pdf

Ecological, evolutionary & environmental sciences study design

All studies must disclose on these points even when the disclosure is negative.

Study description	We study the temporal and spatial variation in diversification rates and ages of angiosperms. We also investigate the current global spatial patterns of plant diversity, age and diversification and evaluate the relationships of tip diversification rates and genus age with genus diversity and temperature and precipitation.
Research sample	We compiled a molecular dataset for 12,539 flowering plant genera based on DNA sequence data of angiosperm species from GenBank. In order to place angiosperm genera in our phylogeny we added taxa for which DNA data was not available using current taxonomy. We also build a global distributional dataset for 13,719 angiosperm genera with a spatial resolution of about 4x4 degrees.
Sampling strategy	We aimed at including data for all described angiosperm genera in our analyses. We sampled DNA data in order to maximize both coverage at the genus level and gene matrix occupancy. Genera for which DNA data was not available were added to the phylogeny

	as polytomies based on the current taxonomy and these porlytomies were then resolved using polytomy resolver approach with BEAST v1.8.0. Distributional data for all angiosperm genera was collected.
Data collection	All data for the present analyses was retrieved from public repositories (e.g. GenBank, eFloras.org, the African Plant Database, TRY, etc.) and from published datasets.
Timing and spatial scale	DNA data was downloaded from GenBank on May 19, 2018. The Catalogue of Life was accessed on May, 2018. The plant list data was obtained on Jan 3, 2015. The Taxonomic Name Resolution Service was accessed on May 18 2019 and the Plant of The World online was accessed in May 2019. Data screening of distributional data from the literature was carried out in the period 2013-2020.
Data exclusions	No data were excluded from the analyses
Reproducibility	Bayesian analyses included two independent runs and were checked for convergence and effective sampling size. We also provide information on the settings and algorithms as well as the datasets used so that analyses can be repeated by other researchers.
Randomization	Randomization was not relevant to our study as we tired to maximize the number of angiosperm genera included in the analyses in order to avoid potential biases due to phylogenetically or spatially biased sampling. However, because taxa without DNA data were aded to the phylogeny based on taxonomy and with the help of polytomy resolver, we performed all analyses using both the tree based on taxa with DNA data and the tree with added genera for which DNA data was not available. In both cases results were highly congruent
Blinding	Blinding is not relevant to our study.

Did the study involve field work? ☐ Yes ☒ No

Reporting for specific materials, systems and methods

We require information from authors about some types of materials, experimental systems and methods used in many studies. Here, indicate whether each material, system or method listed is relevant to your study. If you are not sure if a list item applies to your research, read the appropriate section before selecting a response.

Materials & experimental systems

n/a	Involved in the study	
☒	☐	Antibodies
☒	☐	Eukaryotic cell lines
☒	☐	Palaeontology and archaeology
☒	☐	Animals and other organisms
☒	☐	Clinical data
☒	☐	Dual use research of concern
☒	☐	Plants

Methods

n/a	Involved in the study	
☒	☐	ChIP-seq
☒	☐	Flow cytometry
☒	☐	MRI-based neuroimaging
